# Extended shortwave infrared absorbing antiaromatic fluorenium-indolizine chromophores†

**DOI:** 10.1039/d4sc00733f

**Published:** 2024-07-12

**Authors:** William E. Meador, Matthew A. Saucier, Max R. Tucker, Nicholas A. Kruse, Alexander J. Mobley, Connor R. Brower, Sean R. Parkin, Kensha M. Clark, Nathan I. Hammer, Gregory S. Tschumper, Jared H. Delcamp

**Affiliations:** a University of Mississippi, Department of Chemistry and Biochemistry Coulter Hall, University MS 38677 USA delcamp@olemiss.edu; b Department of Chemistry, University of Kentucky Lexington Kentucky 40506 USA

## Abstract

Shortwave infrared (SWIR, 1000–1700 nm) and extended SWIR (ESWIR, 1700–2700 nm) absorbing materials are valuable for applications including fluorescence based biological imaging, photodetectors, and light emitting diodes. Currently, ESWIR absorbing materials are largely dominated by inorganic semiconductors which are often costly both in raw materials and manufacturing processes used to produce them. The development of ESWIR absorbing organic molecules is thus of interest due to the tunability, solution processability, and low cost of organic materials compared to their inorganic counterparts. Herein, through the combination of heterocyclic indolizine donors and an antiaromatic fluorene core, a series of organic chromophores with absorption maxima ranging from 1470–2088 nm (0.84–0.59 eV) and absorption onsets ranging from 1693–2596 nm (0.73–0.48 eV) are designed and synthesized. The photophysical and electrochemical properties of these chromophores, referred to as FluIndz herein, are described *via* absorption spectroscopy in 17 solvents, cyclic voltammetry, solution photostability, and transient absorption spectroscopy. Molecular orbital energies, predicted electronic transitions, and antiaromaticity are compared to higher energy absorbing chromophores using density functional theory. The presence of thermally accessible diradical states is demonstrated using density functional theory and EPR spectroscopy, while XRD crystallography confirms structural connectivity and existence as a single molecule. Overall, the FluIndz chromophore scaffold exhibits a rational means to access organic chromophores with extremely narrow optical gaps.

## Abstract 2

Shortwave infrared (SWIR, 1000–1700 nm) and extended SWIR (ESWIR, 1700–2700 nm) absorbing materials are valuable for applications including fluorescence based biological imaging, photodetectors, and light emitting diodes.

## Graphical abstract

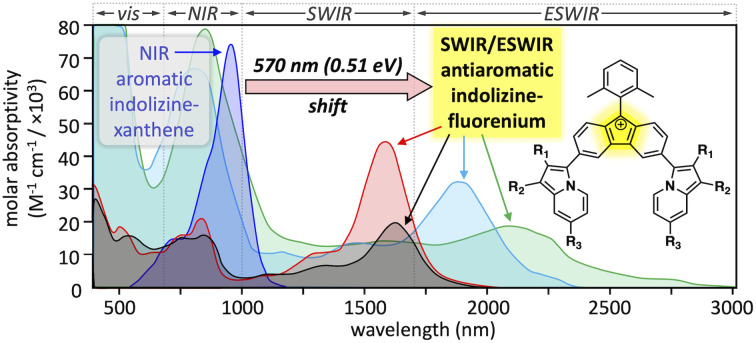

## Introduction

Materials that absorb low energy light in the near infrared (NIR, 700–1000 nm),^1^ shortwave infrared (SWIR, 1000–1700 nm),^2–4^ and extended SWIR (ESWIR, 1700–2700 nm)^5–7^ are valuable for a plethora of optoelectronic applications including but not limited to fluorescence based biological imaging, photodetectors, and light emitting diodes. The field of ESWIR absorbing materials is currently dominated by semiconductors like InGaAs, GaInAsSb, GeSn, and HgCdTe^8,9^ and quantum dots (QDs).^10–12^ These semiconductors are narrow band gap materials commonly used in ESWIR photodetectors whose properties are tunable *via* modification of the nanomaterials size with QDs, and doping in bulk semiconductors. Overall, these ESWIR absorbing semiconductors are heavily inhibited by cost related to both the raw materials and the complex manufacturing processes to make them.^13^ Semiconductor based photodetectors also require cooling to eliminate dark current. While semiconductors dominate commercial applications of SWIR photodetectors, there is active research into the use of organic materials in SWIR photodetectors, especially with regards to organic polymers.^14–18^ Organic photodetectors exhibit low cost manufacturing through solution processability that promises lower costs compared to inorganic materials. These organic photodetectors also commonly work at non-cryogenic temperatures. However, to date the photocurrent response wavelengths do not compete with inorganic semiconducting materials, limiting their application. In this way, the development of novel chromophores for applications in lower energy organic photodetectors is of importance moving forward (Table S3†).

**Fig. 1:**
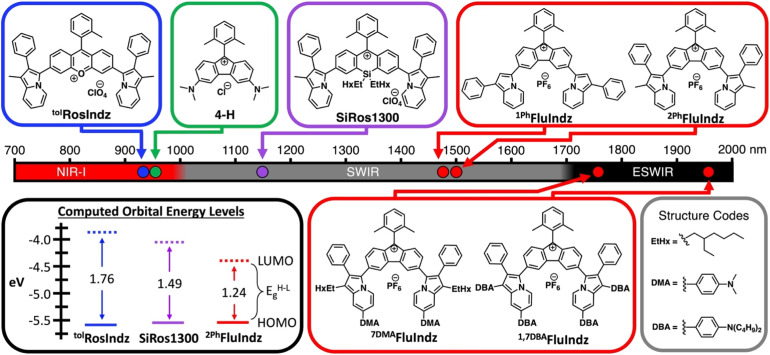
Absorption maxima of indolizine xanthene dyes containing oxygen-based cores (blue), silicon-based cores (purple), and fluorene-based cores (red), and alkyl amine donor-based fluorene dye (green), along with computed orbital energy levels of the highest occupied and lowest unoccupied molecular orbitals (HOMO and LUMO) with the HOMO–LUMO energy gap (*E*^H−L^_g_). Energy levels obtained from density functional theory (DFT) calculations at the B3LYP/6-311G(d,p) level of theory with dichloromethane (DCM) implicit solvation.

There are several classes of small molecule organic chromophores that absorb light in the NIR and SWIR including cyanines,^19–26^ BODIPYs,^27,28^ squaraines,^29–31^ benzothiadiazoles,^32–34^ and xanthenes.^35–38^ While there is an abundance of chromophores from these classes that absorb in the NIR and SWIR, to the best of our knowledge none exhibit the lowest energy absorption maxima (*λ*_abs_) in the ESWIR. In the past few years, xanthene dyes containing heterocyclic donors in place of traditional alkyl amine donors have emerged as a viable means to access SWIR absorption. Both indolizine heterocycles and styryl based donors have been utilized in this respect and have proven to be successful as SWIR fluorescence contrast agents for *in vivo* imaging applications. Along with the use of heterocyclic donors to induce bathochromic shifts in *λ*_abs_ of xanthene chromophores, modifications to the central atom of the xanthene core have also been explored in the literature, with derivatives including nitrogen,^39^ oxygen,^40^ carbon,^41^ silicon,^41,42^ phosphorous,^40^ sulfur,^40^ boron,^43^ and others.^44^ In general, electron donating atoms like nitrogen and oxygen exhibit shorter wavelength *λ*_abs_ and electron withdrawing groups like phosphorous oxides, sulfur oxides, ketones, and trivalent boron tend to shift *λ*_abs_ towards longer wavelengths. Recently, a unique observation was made upon the synthesis of a xanthene analogue containing a fluorene core. By deleting the central atom altogether, generating a five membered central ring as opposed to the traditional six membered central ring, Grzybowski and co-workers were able to induce a dramatic shift in *λ*_abs_ of the rhodamine derivative to 943 nm in dye 4-H (Fig. 1),^36^ a nearly 400 nm (0.95 eV) longer wavelength *λ*_abs_ compared to the traditional oxygen containing rhodamine dye (absorption maxima ∼548 nm). This observation was attributed to antiaromaticity which was observed computationally *via* nucleus-independent chemical shift (NICS) analysis. In the analysis, the central five membered ring of the fluorenium dyes demonstrated a paratropic ring current, resulting in a positive chemical shift indicative of antiaromaticity. Conversely, the six membered central ring of classic oxygen-containing xanthene dyes demonstrated a diatropic ring current, resulting in a negative chemical shift indicative of aromaticity. This was an intriguing use of antiaromaticity which has been shown to induce low energy electronic transitions through a “Jahn–Teller-like” reorganization of molecular orbitals in antiaromatic heterocycles.^45–47^ With this knowledge in hand, a heterocyclic indolizine donor-based fluorene dye was pursued in an attempt to produce a series of low energy absorbing small molecule organic chromophores, deemed FluIndz (Fig. 1).

## Results and discussion

### Synthesis

**Scheme 1:**
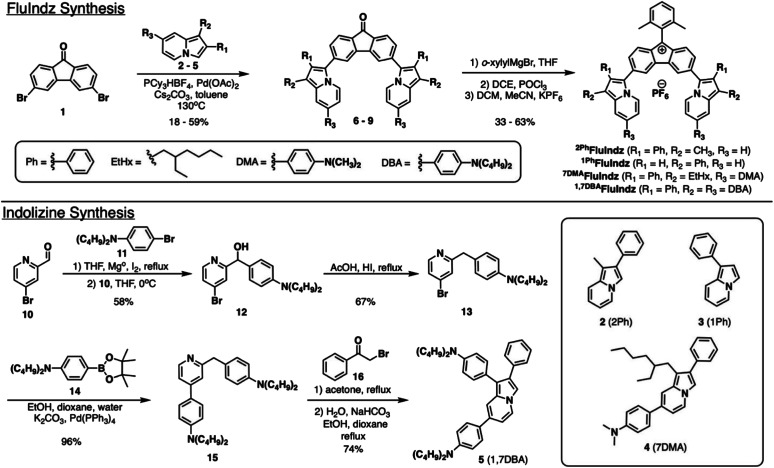
Synthesis of FluIndz dyes (top) and 1,7DBA indolizine donor (bottom).

Synthesis of the FluIndz dyes began with making the indolizine donors. 2Ph,^48^ 1Ph,^49^ and 7DMA^23^ indolizine donors (2–4, Scheme 1) were synthesized as previously described, and 1,7DBA indolizine donor (5) was synthesized as shown in Scheme 1. To make 5, a Grignard reaction was run *via* the formation of the Grignard reagent from bromide 11 and subsequent reaction with aldehyde 10 to yield the alcohol 12 in 58% yield. The alcohol was deoxygenated using acetic acid and hydroiodic acid to yield 13 in 67% yield. 13 was obtained as a mixture after multiple chromatographic separation attempts and was used in the following reaction as a mixture. A Suzuki reaction using 13 and 14 (synthesized as previously described^50^) yielded 15 in 96% yield, which was subsequently reacted with 2-bromoacetophenone, 16, to yield 1,7DBA indolizine donor, 5, in 74% yield. With the indolizine donors in hand, a palladium catalyzed C–H activation reaction was run utilizing the commercially available dibromofluorenone core (1) and the respective indolizine donor (2–5) to produce the coupled fluorenone products in yields ranging from 18–59% (Scheme 1). From the coupled fluorenones, 6–9, Grignard reactions were run with *ortho*-xylylmagnesium bromide to yield the intermediate alcohols. Several acidic conditions were evaluated using the 2Ph derivative to remove the alcohol and form the final dye without success. Aqueous acids like HClO_4_ and HCl commonly used for alcohol removal in xanthene dyes^35,36,51^ were observed to yield no product *via* SWIR absorption monitoring. An anhydrous condition using trifluoroacetic acid in DCM solution^44,52^ was observed to produce the desired product; however, the product was observed to rapidly decompose during isolation. Alternative anhydrous acidic conditions employing triflic acid and hydrofluoroboric acid in DCM solution were also observed to produce the desired product, but at very low yields (<10%) with the predominant product being a green chromophore absorbing near ∼800 nm. Finally, a non-Brønsted acid based condition using POCl_3_, as used previously for the RhodIndz and VIX series,^35,37,53^ was observed to work well with a yield of 63% across both the Grignard reaction and alcohol removal step for the 2Ph derivative. The POCl_3_ conditions yielded the final dyes but with poor solubility and difficult purification, so an anion exchange reaction to give the PF_6_^−^ salt was performed following alcohol removal to aid in purification and solubility of the FluIndz dyes. The POCl_3_ conditions were used for the rest of the series and produced the final products in yields ranging from 33–63%.

### Photophysical properties

**Fig. 2:**
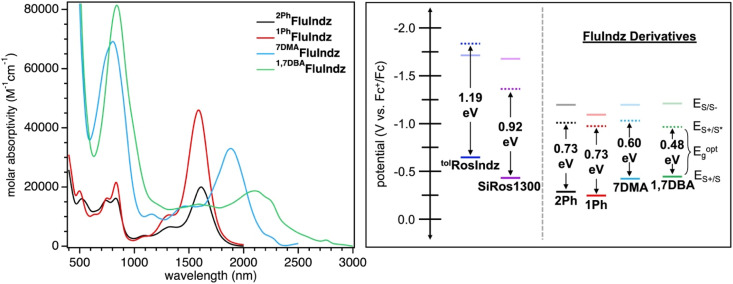
Molar absorptivity of FluIndz dyes in CS_2_ (left) and electrochemical potentials (*E*_S+/S_, *E*_S/S−_, and *E*_S+/S*_) with optical energy gaps in DCM (right). Electrochemical potentials shown are referenced to Fc^+^/Fc at 0.00 V in DCM with a 0.1 M Bu_4_NPF_6_ supporting electrolyte.

With the chromophores in hand, the FluIndz dyes were studied for their solution absorption profiles in a variety of solvents of varying polarity from dioxane (dielectric constant = 2.21) to dimethyl sulfoxide (DMSO, dielectric constant = 47) to understand how solvent selection affects the photophysical properties of these dyes (Fig. S1–S4†). The absorption spectrum of the FluIndz dyes were observed to be heavily solvent dependent, with some solvents retaining the characteristic “cyanine-like” π → π* electronic features, wherein the lowest energy transition was relatively sharp and intense, while other solvents instead induced broadened features indicative of charge transfer behavior (n → π*) with more intense higher energy transitions. In general, the FluIndz dyes tended to maintain their cyanine-like electronic features in less polar, non-coordinating solvents like toluene, carbon disulfide (CS_2_), chloroform (CHCl_3_), chlorobenzene (PhCl), dichloromethane (DCM), and 1,2-dichloroethane (DCE). Exceptions to this include: acetic acid (AcOH), which worked well for ^2Ph^FluIndz and ^1Ph^FluIndz but not ^7DMA^FluIndz and ^1,7DBA^FluIndz (likely due to protonation of the aryl amines) and ethanol (EtOH), which worked well for ^7DMA^FluIndz and ^1,7DBA^FluIndz, but not ^2Ph^FluIndz and ^1Ph^FluIndz. Ethereal solvents like dioxane and tetrahydrofuran (THF) resulted in broadened features in all dyes except ^1Ph^FluIndz, and despite its low polarity (dielectric constant = 6), ethyl acetate (EtOAc) also resulted in broadened features in all the FluIndz dyes studied. Overall, CS_2_ was observed to be the best solvent for absorption spectroscopy considering the minimal amount of solvent absorption interference in the SWIR and ESWIR, the sharp features observed, and the lowest energy *λ*_abs_ across all solvents. Therefore it was selected alongside DCM (used for comparison across the literature, Fig. S5†) to collect further photophysical data (Fig. 2). While the absorption bands in CS_2_ and DCM appear broad for these materials when plotted in nm (especially for the longer wavelength absorbing derivatives ^7DMA^FluIndz and ^1,7DBA^FluIndz), when plotted in a scale that is linear with respect to energy, like eV, these compounds exhibit comparable absorption peak widths as similar compounds that absorb at higher energies (Fig. S6†).

The FluIndz dyes all demonstrated longer wavelength *λ*_abs_ in CS_2_ compared to DCM, with *λ*_abs_ values of 1590, 1620, 1882, and 2088 nm in CS_2_ and *λ*_abs_ of 1470, 1500, 1756, and 1956 nm in DCM for ^1Ph^FluIndz, ^2Ph^FluIndz, ^7DMA^FluIndz, and ^1,7DBA^FluIndz, respectively (Fig. 2 and S5,†Table 1). The absorption onset values (*λ*_onset_, determined using the onset program^54^) were also lower in energy for most of the FluIndz dyes in CS_2_ compared to DCM. Overall, *λ*_abs_ of the FluIndz dyes are substantially lower in energy than previous indolizine donor-based xanthene derivatives. A shift of *λ*_abs_ to longer wavelengths can be observed in the structurally comparable series (same donor and varied cores) with a 570 nm (0.51 eV) shift from ^tol^RosIndz^38^ (oxygen-containing core, *λ*_abs_ = 930 nm) to ^2Ph^FluIndz (fluorene core, *λ*_abs_ = 1500 nm) and a 360 nm (0.26 eV) shift from SiRos1300 (ref. 51) (silicon-containing core, *λ*_abs_ = 1140 nm) to ^2Ph^FluIndz (fluorene core, *λ*_abs_ = 1500 nm) (Fig. 1). This demonstrates the value of the antiaromatic cyclopentadienyl cationic core as a means to shift *λ*_abs_ of the xanthene scaffold towards lower energies. The use of indolizine donors in ^2Ph^FluIndz (*λ*_abs_ = 1500 nm) also results in a sizeable shift of 557 nm (0.49 eV) in *λ*_abs_ towards longer wavelengths compared to the alkyl amine donor-based 4-H (ref. 36) (*λ*_abs_ = 943 nm).

**Table 1:** Photophysical and electrochemical properties of the FluIndz dyes in DCM and CS_2_ solution. Electrochemical potentials shown are referenced to Fc^+^/Fc at 0.00 V in DCM with a 0.1 M Bu_4_NPF_6_ supporting electrolyte. Photophysical data reported for the lowest energy transition

Dye	Solvent	*λ*_abs_ (nm)	*λ*_onset_ (nm)	*ε* (M^−1^ cm^−1^)	*E*_S+/S_ (V)	*E*_S/S−_ (V)	*E* ^opt^_g_ (eV)	*E*_S+/S*_ (V)
^2Ph^FluIndz	DCM	1500	1696	23 400	−0.28	−1.21	0.73	−1.01
	CS_2_	1620	1794	20 000	—	—	—	—
^1Ph^FluIndz	DCM	1470	1693	39 900	−0.25	−1.15	0.73	−0.98
	CS_2_	1590	1761	46 000	—	—	—	—
^7DMA^FluIndz	DCM	1756	2076	27 500	−0.43	−1.21	0.60	−1.03
	CS_2_	1882	2166	33 000	—	—	—	—
^1,7DBA^FluIndz	DCM	1956	2596	23 000	−0.47	−1.22	0.48	−0.95
	CS_2_	2088	2547	18 700	—	—	—	—
4-H^a^	H_2_O	943	—	14 700	—	—	—	—
^tol^RosIndz^b^	Toluene/DCM	930	1042	79 500	−0.65	−1.70	1.19	−1.84
SiRos1300 (ref. 51)	DCM	1140	1350	115 000	−0.45	−1.68	0.92	−1.37

a Reference dye in H_2_O with ∼5% DMSO cosolvent.^36^

b Photophysical measurements originally taken in toluene,^38^ electrochemical measurements taken in DCM herein.

^1,7DBA^FluIndz was observed to have the lowest molar absorptivity (*ε*) for its lowest energy peak in both DCM and CS_2_, with *ε* of 23 000 and 18 700 M^−1^ cm^−1^, respectively. This observation is likely due to the steric interaction of the *N*,*N*-dimethylaniline (DMA) group at the 1-position of the indolizine donor and the phenyl group at the 2-position of the indolizine donor resulting in a lowering of the *ε* as observed previously.^23,51^^2Ph^FluIndz had the next lowest *ε* values of 23 400 and 20 000 M^−1^ cm^−1^ in DCM and CS_2_, respectively. ^1Ph^FluIndz and ^7DMA^FluIndz both had higher *ε* values in CS_2_ compared to DCM, where ^1Ph^FluIndz had a *ε* of 39 900 and 46 000 M^−1^ cm^−1^ in DCM and CS_2_, respectively, and ^7DMA^FluIndz had a *ε* of 27 500 and 33 000 M^−1^ cm^−1^ in DCM and CS_2_, respectively. The removal of the phenyl group at the 2-position is thus observed to result in a sizeable increase in *ε* of ^1Ph^FluIndz, with a *ε* in CS_2_ more than double that of ^2Ph^FluIndz. Removal of the phenyl group at the 2-position likely allows for increased planarization across the chromophore π-system, resulting in better orbital overlap of the frontier molecular orbitals leading to a higher *ε*. Overall, the FluIndz chromophores are observed to have lower *ε* than both ^tol^RosIndz (*ε* = 79 500 M^−1^ cm^−1^) and SiRos1300 (*ε* = 115 000 M^−1^ cm^−1^) which is expected given the typically low *ε* values antiaromatic chromophores display in the literature.^36,43^ The trend observed across the series (antiaromatic < oxygen < silicon), with respect to *ε*, is consistent with previous reports.^43,44^ Higher energy absorption peaks and associated *ε* values are given in Tables S1 and S2.† Emission spectroscopy was not collected herein due to the predicted emission range of the chromophores (1640–2320 nm based on a stokes shift of 0.10 eV) requiring highly optimized spectrometer setups as described previously.^51^ However, transient absorption spectroscopy (TAS) was obtained for ^1Ph^FluIndz in DCM. The dye shows excited state absorption peaks at 443 and 1050 nm within 5 ps after excitation with 1500 nm light (Fig. S7†). Excited state lifetime values were not obtained due to poor fitting to exponential decay equations.

### Electrochemical properties

The FluIndz derivatives were also studied *via* electrochemistry in DCM solution to better understand the redox properties of these materials (Tables 1 and S4,†Fig. 2 and S8–S12†). The ground state oxidation potentials (*E*_S+/S_) of the FluIndz derivatives were observed to exhibit a trend based on indolizine donor strength. Added peripheral amine donors shifted potentials more negative *versus* ferrocene, where ^1Ph^FluIndz was the most positively shifted and ^1,7DBA^FluIndz was the most negatively shifted. *E*_S+/S_ was thus observed to be −0.25, −0.28, −0.43, and −0.47 V *vs.* Fc^+^/Fc^55–57^ for ^1Ph^FluIndz, ^2Ph^FluIndz, ^7DMA^FluIndz, and ^1,7DBA^FluIndz, respectively. In comparing ^2Ph^FluIndz to ^tol^RosIndz and SiRos1300, the *E*_S+/S_ was observed to be most negative for ^tol^RosIndz at −0.65 V (Fig. S12†), second most negative for SiRos1300 at −0.45 V,^51^ and least negative for ^2Ph^FluIndz at −0.28 V. In this way, switching the core from the donating oxygen to the neutral silicon to the antiaromatic fluorene results in a positive shift in *E*_S+/S_. For the ground state reduction potentials (*E*_S/S−_), ^2Ph^FluIndz, ^7DMA^FluIndz, and ^1,7DBA^FluIndz were all grouped within 10 mV of one another (−1.21 to −1.22 V), with ^1Ph^FluIndz being shifted towards a more positive potential at −1.15 V. ^2Ph^FluIndz values are considerably more positive than *E*_S/S−_ of both ^tol^RosIndz and SiRos1300, which have *E*_S/S−_ values of −1.70 and −1.68 V, respectively. The optical energy gap of the materials (*E*^opt^_g_) was determined from *λ*_onset_ in DCM solution *via* the equation 1240/*λ*_onset_ = *E*^opt^_g_. *E*^opt^_g_ was thus determined to be 0.73, 0.73, 0.60, and 0.48 eV for ^2Ph^FluIndz, ^1Ph^FluIndz, ^7DMA^FluIndz, and ^1,7DBA^FluIndz, respectively. The excited state oxidation potential (*E*_S+/S*_) could then be calculated for the FluIndz dyes *via* the equation *E*_S+/S*_ = *E*_S+/S_ − *E*^opt^_g_. *E*_S+/S*_ does not follow a trend based on the indolizine donor and is observed to be −0.95, −0.98, −1.01, and −1.03 for ^1,7DBA^FluIndz, ^1Ph^FluIndz^2Ph^FluIndz, and ^7DMA^FluIndz, respectively. The large shift in *E*_S/S−_ and *E*_S+/S*_ towards more positive potentials observed in the antiaromatic FluIndz dyes compared to previous indolizine xanthenes is consistent with previous computational predictions, which indicate that the primary source of the lower energy absorption comes from a decrease in the lowest unoccupied molecular orbital (LUMO) energy.^36,44^

### Computational analysis

**Fig. 3:**
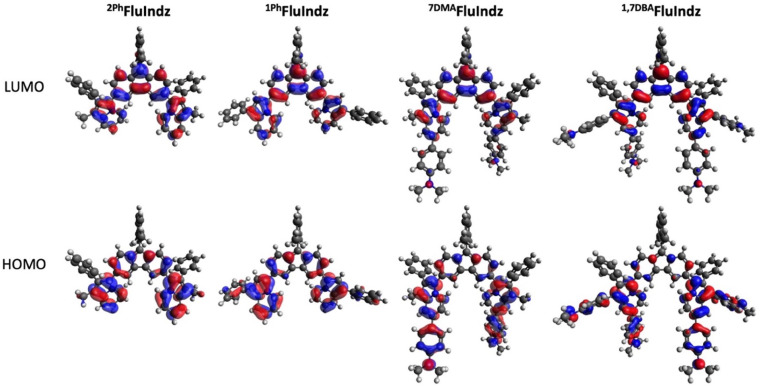
Frontier molecular orbitals including HOMO (bottom) and LUMO (top) of FluIndz dyes ^2Ph^FluIndz (left), ^1Ph^FluIndz (center left), ^7DMA^FluIndz (center right), and ^1,7DBA^FluIndz (right).

Consistent with previous studies of NIR and SWIR dyes,^23,38,48,51,58^ all computational calculations were conducted at the B3LYP/6-311G(d,p)^59–61^ level of theory using a DCM polarizable continuum model^62–65^ as implicit solvent with Gaussian16 (ref. 66) software. Visual analysis of the frontier molecular orbitals (Fig. 3), derived from density functional theory (DFT), demonstrates the HOMO is primarily located on the indolizine donors, with some distribution on the core, whereas the LUMO is heavily distributed across the core of the chromophore as well as the indolizine heterocycle. The DMA groups are observed to have significant contribution to the HOMO, but little contribution to the LUMO. This coincides with the electrochemical measurements wherein the addition of the DMA groups shifts *E*_S+/S_ towards more negative potentials but has little effect on *E*_S/S−_ or *E*_S+/S*_. Time dependent density functional theory (TD-DFT) corroborates the similar maximum *λ*_abs_ of ^2Ph^FluIndz and ^1Ph^FluIndz at 1500 and 1470 nm (0.83 and 0.84 eV), respectively, with vertical transitions (VT) observed at 1131 and 1151 nm (1.10 and 1.08 eV), respectively (Table S5†). ^7DMA^FluIndz is accurately predicted to be the second lowest energy absorbing chromophore, with a VT observed at 1405 nm (0.88 eV), and ^1,7DBA^FluIndz is accurately predicted to be the lowest energy absorbing chromophore, with a VT observed at 1635 nm (0.76 eV). TD-DFT predicts the lowest energy absorbing dye ^1,7DBA^FluIndz to have the highest oscillator strength in the FluIndz series (0.68), followed by ^1Ph^FluIndz (0.63), then ^7DMA^FluIndz (0.55), and finally ^2Ph^FluIndz (0.52) having the lowest. TD-DFT also predicts higher energy transitions with significant oscillator strengths (>0.2) that correspond to higher energy features from 700–1000 nm in the solution absorption spectra (Table S5†).

Calculations were also performed on the previous oxygen (^tol^RosIndz) and silicon (SiRos1300) derivatives to compare with ^2Ph^FluIndz (Fig. 1 and Table S5†). The trend in VT energy correlates to the decrease in maximum *λ*_abs_ energy according to the trend ^tol^RosIndz > SiRos1300 > ^2Ph^FluIndz. The oscillator strength values follow the trend SiRos1300 > ^tol^RosIndz > ^2Ph^FluIndz. Frontier molecular orbital energies of these three dyes show the HOMOs being close in energy within 64 mV (Fig. 1 and Table S5†). The primary contribution to the decreasing HOMO–LUMO energy gap (*E*^H−L^_g_) is the decreasing LUMO energy according to the trend: ^tol^RosIndz > SiRos1300 > ^2Ph^FluIndz (455 mV difference, Fig. 1 and Table S5†).

The adiabatic singlet–triplet energy gaps (Δ*E*^ad^_ST_) were determined as the difference between the total energies of the optimized singlet ground state (S_0_) and first triplet state (T_1_) for each dye (Table S6†). Interestingly, the FluIndz series displayed relatively small Δ*E*^ad^_ST_ gaps (3.6–9.7 kcal mol^−1^) compared to ^tol^RosIndz (22.6 kcal mol^−1^) and SiRos1300 (16.2 kcal mol^−1^), implying possible thermally accessible triplet states for the FluIndz dyes. Materials with thermally accessible diradical triplet states in the literature commonly exhibit an experimentally determined Δ*E*_ST_ of ∼4–5 kcal mol^−1^, while a Δ*E*_ST_ > 15 kcal mol^−1^ is commonly observed to exist exclusively as a paired singlet.^67^ Although Δ*E*_ST_ is likely overestimated herein since the B3LYP functional often overestimates this energy gap,^68,69^ the data suggest that the FluIndz chromophores do not exist exclusively as a paired singlet while ^tol^RosIndz and SiRos1300 have lower diradical triplet populations.

**Fig. 4:**
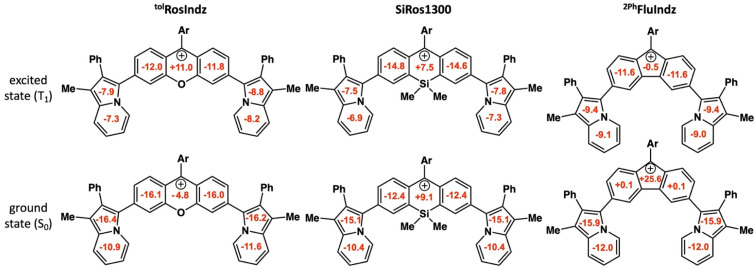
NICS_ZZ_(avg) (in ppm) for both the singlet ground state (S_0_) and first triplet state (T_1_) as the excited state. Ar = *o*-tolyl (^tol^RosIndz) or *o*-xylyl (SiRos1300 and ^2Ph^FluIndz).

NICS calculations were performed using the optimized geometries for the singlet ground state (S_0_) and first triplet state (T_1_) for ^tol^RosIndz, SiRos1300, and ^2Ph^FluIndz (Fig. 4). Negative NICS_ZZ_ values at 1 Å above/below a ring correspond to aromaticity in that ring while positive values correspond to antiaromaticity and values near zero signify that the ring is nonaromatic. NICS_ZZ_(avg) values are reported as the average of the values at 1 Å above (NICS_ZZ_(+1)) and below (NICS_ZZ_(−1)) each ring for the 7 rings of interest. Similar to previous reports,^58,70^ the T_1_ state is used as an approximation of the excited state since the magnetic shielding tensors are not available *via* TD-DFT for the S_1_ state with our computational software. The central ring (Ring D, see Fig. S13† for ring naming scheme, which proceeds alphabetically starting from the bottom left ring and moving clockwise) is the primary site of aromaticity or antiaromaticity induced by the core. In the ground state, ^tol^RosIndz is aromatic in all 7 rings. In the excited state, ring D flips from being aromatic (−4.8 ppm) to being antiaromatic (+11.0 ppm), while the adjacent rings (rings C and E) remain aromatic, albeit less so (S_0_ = −16.1 and −16.0 ppm, T_1_ = −12.0 and −11.8 ppm). Interestingly, ring D in SiRos1300 is antiaromatic (+9.1 ppm) with all the other rings being aromatic in the ground state. In the excited state, ring D remains antiaromatic (+7.5 ppm) and the adjacent rings (rings C and E) become slightly more aromatic than in the ground state (S_0_ = −12.4 and −12.4 ppm, T_1_ = −14.8 and 14.6 ppm). Lastly, ^2Ph^FluIndz is antiaromatic in ring D (+25.6 ppm) with nonaromatic adjacent rings (rings C and E). In the excited state, ring D becomes nonaromatic (−0.5 ppm) and rings C and E both become more aromatic than in the ground state (S_0_ = +0.1 ppm each, T_1_ = −11.6 ppm each). The indolizine rings (rings A, B, F, and G) in all three dyes behave nearly the same: aromatic in the ground state and less aromatic in the excited state. The trend observed in aromaticity of ring D between the ground and excited states is similar to the trend expected from Baird's rule^71^ for closely related systems where a 4*n* π-electron antiaromatic ground state becomes more aromatic in the excited state, and *vice versa*. Here, the aromatic ground state of ^tol^RosIndz flips to being antiaromatic in the excited state, SiRos1300 remains antiaromatic in both states, and the antiaromatic ground state of ^2Ph^FluIndz becomes nonaromatic in the excited state. NICS_ZZ_(avg) values and profiles can be found in the ESI (Tables S7–S9, Fig. S14–S20).†

### Paramagnetic properties

Upon investigation into other fluorene-based materials in the literature, it was observed that some fluorene materials exhibit broad ^1^H-NMR spectra with no discernible peaks at room temperature^72^ or elevated temperatures^73^ due to thermally accessible diradical triplet states. Unlike previous indolizine–xanthene^35,38,51^ and fluorenium^36^ dyes with well resolved ^1^H-NMR spectra, the FluIndz ^1^H-NMR spectra are similarly broad and featureless in the aromatic region. Both this NMR observation and the computational evidence of possible thermally accessible triplet states led to the investigation of possible paramagnetic properties in these dyes.

First, the ^1^H-NMR spectra of the FluIndz dyes were taken in DCM at both room temperature (25 °C) and at −30 °C (the lower limit of the available instrumentation) to see if the resolution of the spectrum could be improved as seen in the literature. However, little to no change was observed in the ^1^H-NMR spectra, possibly due to not reaching low enough temperatures (Fig. S63, S65, S67, and S69†). Next, electron paramagnetic resonance (EPR) spectroscopic measurements were conducted for the FluIndz dyes in DCM (Fig. S25–S28†). All four FluIndz dyes demonstrated an EPR signal, exhibiting unpaired electrons indicative of diradical behavior. EPR measurements were also conducted for SiRos1300 and ^tol^RosIndz to see if these materials exhibited diradical nature (Fig. S29 and S30†). Interestingly, SiRos1300 was observed to be EPR active, indicating the presence of unpaired electrons, while ^tol^RosIndz was not.

**Fig. 5:**
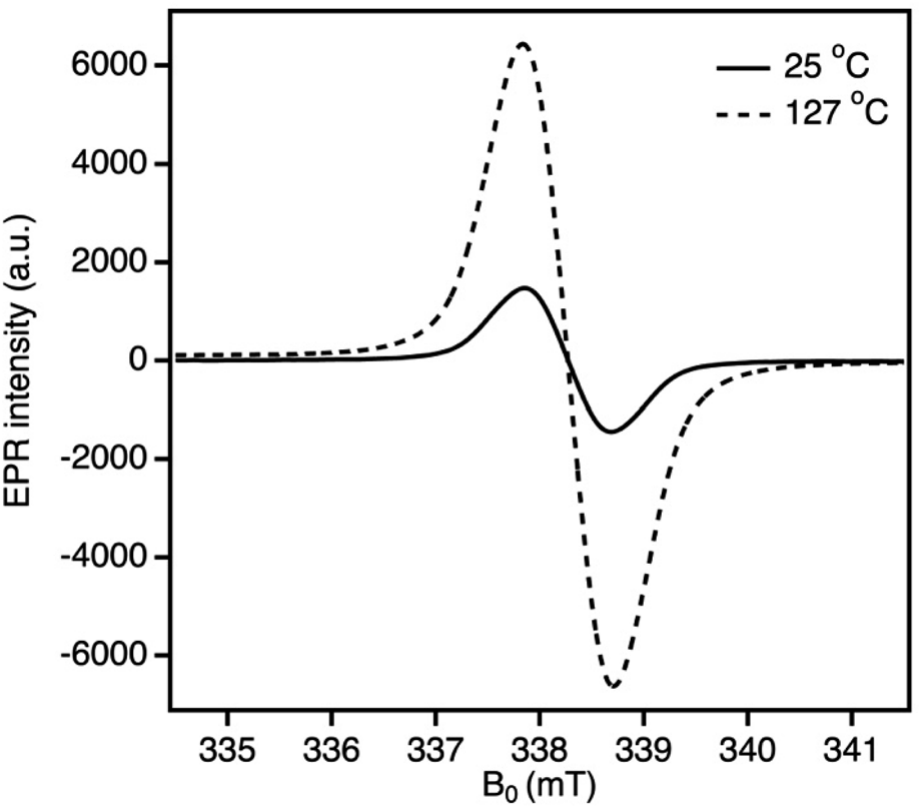
Variable temperature EPR experiment: spectrum of the first derivative absorption of ^2Ph^FluIndz in PhCl solution from 25 °C (*g* = 2.0034) up to 127 °C (*g* = 2.0035).

Variable temperature (VT) EPR was conducted for ^2Ph^FluIndz from −95 to 127 °C. As the temperature decreased to −95 °C (which is 65° lower than the VT ^1^H-NMR experiment), the integrated absorption intensity decreased to 65% of the signal at room temperature (25 °C, Fig. S35†). When ^2Ph^FluIndz was heated to 127 °C in PhCl solution, the integrated absorption intensity significantly increased (500%) compared to the signal at room temperature (Fig. 5 and S36†). This type of behavior is common for molecules with an open-shell singlet ground state and a thermally accessible triplet state.^74,75^ A radical control sample of TEMPO was observed to have a nearly identical integrated absorption intensity for both the elevated and room temperature spectra indicating no temperature dependence (Fig. S37†).

Evans method NMR measurements were also performed on all four dyes to determine their respective spin counts (Fig. S31–S34†). The effective magnetic moments (*μ*_eff_) at room temperature were calculated in the same way as previous literature^76–79^ and are 0.624 *μ*_B_ for ^2Ph^FluIndz, 0.726 *μ*_B_ for ^1Ph^FluIndz, 0.405 *μ*_B_ for ^7DMA^FluIndz, and 0.792 *μ*_B_ for ^1,7DBA^FluIndz. These small *μ*_eff_ values indicate that the dyes do not exist as purely diradical single molecules (*μ*_eff_ = ≥2.0 *μ*_B_), which is a phenomenon referred to as the “biradical paradox” and is commonly observed in conjugated organic biradical systems.^67,73,78,80^ The reason for this observation is most likely either aggregation leading to antiferromagnetic intermolecular spin pairing^80,81^ or thermal population of the diradical triplet state.^67,72,73^ Characterization of the FluIndz dyes including XRD crystallography, mass spectroscopy (MS), and elemental analysis (EA) suggest that the dyes exist as single molecules and rules out radical polymerization leading to the lower than expected *μ*_eff_ values (as seen for Tschitschibabin's hydrocarbon which forms oligomers in solution).^67^

Importantly, the trend observed in computational Δ*E*_ST_ is consistent with the paramagnetic data. As the optical gap decreases, Δ*E*_ST_ decreases in magnitude, and the population of thermally accessed diradical states becomes more prevalent. We have previously observed lower energy fluorophores exhibiting longer excited state lifetimes.^51^ This inverse phenomenon was suggested to be due to mixing of singlet and triplet states, which these EPR measurements strongly support.

### X-ray crystallography

**Fig. 6:**
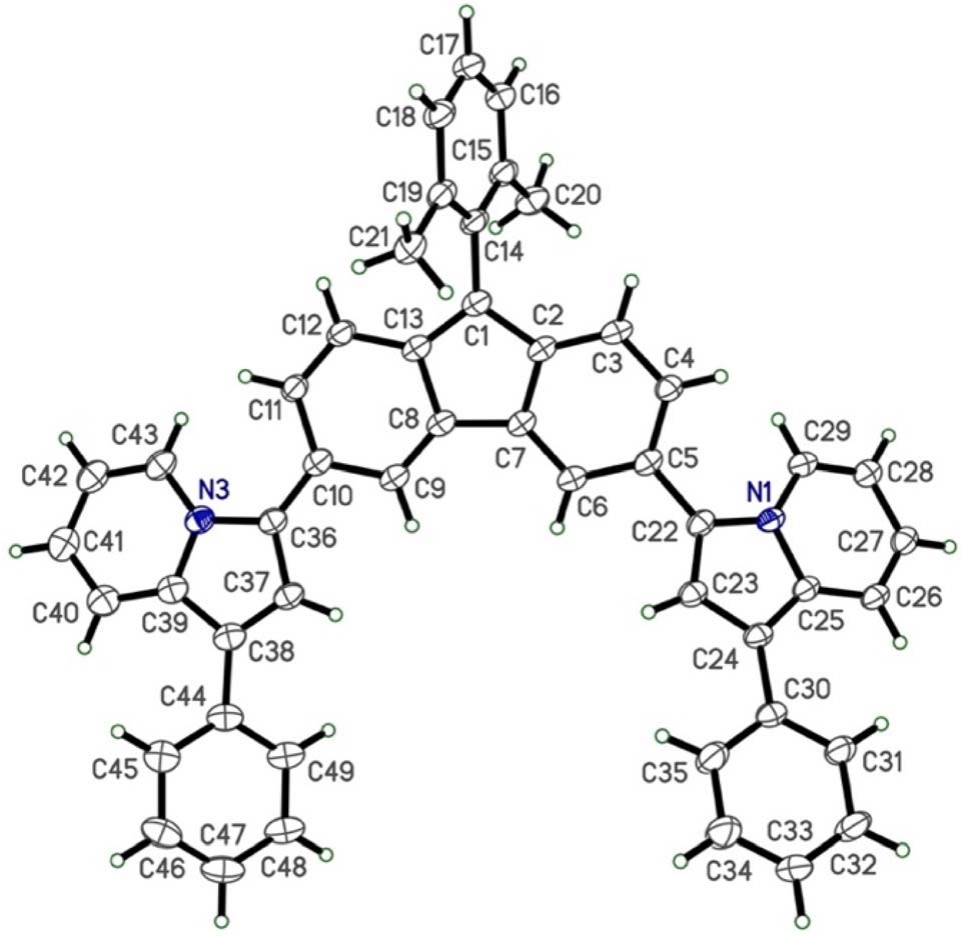
XRD crystal structure of ^1Ph^FluIndz dye (the counteranion and solvent molecules have been omitted to enhance clarity).

X-ray diffraction (XRD) crystallography was collected for ^1Ph^FluIndz to better understand the geometry of the antiaromatic chromophore (Fig. 6), especially due to the lack of discernible NMR signals observed. Herein, XRD crystallography was only successfully gathered for ^1Ph^FluIndz due to ^2Ph^FluIndz growing in very small, thin needles that were not suitable for adequate diffraction and ^7DMA^FluIndz and ^1,7DBA^FluIndz not preferentially forming crystals due their long alkyl chains. Crystals of ^1Ph^FluIndz could be grown *via* vapor diffusion of anhydrous diethyl ether into 1.0 mL of anhydrous acetonitrile containing ∼10 mg of the dye (with the vials being backfilled with N_2_ prior to sealing as a precaution). The chromophore formed dark colored, shiny crystals. For ^1Ph^FluIndz, the dye molecules were observed to crystallize in an orientation where the indolizine donors were face to face on top of one another orienting in a head-to-tail direction (Fig. S21–S24†). The result of this packing was a non-symmetric orientation of the indolizine donors with the core (39.1° dihedral angle for C6, C5, C22, and C23 and a 13.8° dihedral angle for C9, C10, C36, and C37) resulting in differing bond lengths between the two donors (0.01–0.05 Å difference between the same bonds of each donor). Prior crystals of indolizine donor-based fluorophores have exhibited greater symmetry in their molecular orientation, with the indolizine donors having nearly identical dihedral angles and bond lengths.^35,48,82^ The average bond length of the indolizine donors in the ^1Ph^FluIndz was still observed to be nearly identical to previously reported values for indolizine donor-based fluorophores. The bond lengths within the core were in good agreeance with previously observed fluorenium cation crystals,^36,72,83^ and exhibited the same variation in bond length on the two sides of the fluorenium core. Atomic coordinates and bond lengths and angles can be found in Tables S10–S12 in the ESI.†

### Photostability

**Fig. 7:**
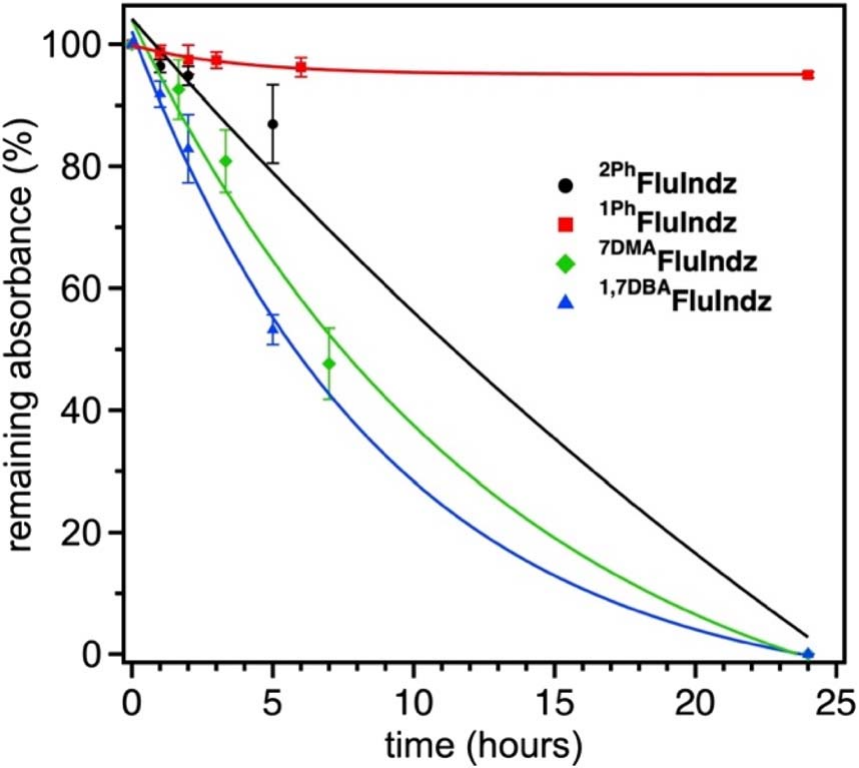
Photostability of FluIndz dyes under 1 sun irradiation in DCM solution. Trials were done in triplicate (error bars given as the standard deviation in % absorbance remaining at each time point) and each data set is fit with an exponential function.

The FluIndz dyes were studied for their relative photostability in DCM solution under ambient atmosphere and 1 sun irradiation using a Pico Solar Simulator from G2V Optics Inc. The photostability was determined by taking the absorption of the samples (done in triplicate) at regular time intervals over a 24 hour period (Fig. 7). ^1Ph^FluIndz was observed to have the greatest photostability of all the derivatives tested herein, maintaining ∼95% of its initial absorbance over the 24 hour period. Once the 24 hour period was over, the samples were left on the benchtop under ambient conditions for an additional week and observed to maintain >90% of their initial absorbance. The other FluIndz derivatives were observed to have no detectable absorbance signal after 24 hours, and their half-lives are estimated from exponential fits of the data. ^2Ph^FluIndz was observed to have the second greatest photostability with a half-life of ∼11.5 hours, followed by ^7DMA^FluIndz with a half-life of ∼7.5 hours, and lastly ^1,7DBA^FluIndz with a half-life of ∼6 hours. Interestingly, the trend in photo stability aligns with the trends in both *λ*_abs_ and *E*_S+/S_, wherein the lower energy absorbing and more negatively shifted *E*_S+/S_ have the shortest half-lives. While this relative stability study gives a comparison of the chromophore stability based on structural changes, there is no practical threshold described herein which would support or prevent these dyes' use in a practical application. Stability is highly environmentally dependent, and many chromophores are used in high performing devices for photodetectors and encapsulated in nanoemulsions for biological imaging that are not stable in ambient environments for extended periods. The improved relative stability of ^1Ph^FluIndz does suggests this molecular design will impart greater stability in ambient environments, but this does not imply the other chromophores are unsuitable for specialized environments such as inert environments where most IR photodetectors are prepared for example. In an inert environment, these dyes are likely indefinitely stable since they have been stored without degradation for over a year.

## Experimental

### General information

Reagents and solvents used in this study were purchased from Ambeed, TCI, Sigma Aldrich, Acros Organics, VWR, and Thermo Fischer Scientific and were used as received without further purification. Thin-layer chromatography (TLC) was conducted with Sorbtech Silica XHL TLC Plates (Support: Glass backed and Thickness: 250 μm) and visualized with a UV lamp. Flash column chromatography was performed using a Teledyne CombiFlash Rf + system. The silica gel cartridges were purchased from Luknova SuperSep (FC003012, 50 μm). ^1^H and ^13^C NMR spectra were recorded on a Bruker Ascend-300 (300 MHz) and a Bruker Ascend-400 (400 MHz) spectrometer using deuterated solvents. *J* values are expressed in Hz and chemical shifts are in ppm using residual solvent as a reference (CDCl_3_ at 7.26 ppm, DMSO-d_6_ at 2.50 ppm, CD_3_CN at 1.94 ppm, and C_6_D_6_ at 7.16 ppm). Singlet (s), doublet (d), doublet of doublets (dd), triplet (t), multiplet (m), multiple signals (ms), broad (br), and apparent (ap) are designated as ^1^H-NMR multiplicity patterns. EPR measurements were collected using X-band Magnettech ESR5000 spectrometer. The hyperfine coupling was not resolved and *g*-values are calculated according to the apparent isotropic signals observed. For electrospray ionization (ESI) high-resolution mass spectrometry (HRMS), quadruple-TOF was used to obtain the data, both in positive and negative modes, with a Waters Synapt HDMS or Orbitrap Exploris 240 to obtain the data in positive mode with a spray voltage of 3600 V, a resolution of 240 000, the ion transfer tube temperature set at 300 °C, and the mass analyzer set to the 200–2000 Da range. Infrared spectra were recorded with an Agilent Cary 660 attenuated total reflection-Fourier transform infrared (ATR-FTIR) spectrometer. All the absorption profiles were recorded on a Cary 5000 UV-Vis-NIR spectrophotometer set to double beam mode. All spectra were taken with the dyes at a concentration of 1.8 × 10^−5^ M in a 1.0 cm pathlength cuvette and are smoothed with a LOESS functional to bring clarity to some of the noise arising from solvent absorption in the SWIR and ESWIR regions. The same experimental setup used for transient absorption spectroscopy has been described previously.^84^ Briefly, the 800 nm, 100 fs fundamental output from a femtosecond amplifier (Coherent Astrella, Santa Clara, California) was split with an 85–15 beamsplitter to generate pump and probe beams. To generate the pump excitation wavelength for these samples (*λ*_exc_ = 1500 nm), the reflected portion of the fundamental output was routed through an optical parametric amplifier (Light Conversion Topas OPerA Solo, Vilnius, Lithuania). The excitation wavelength of 1500 nm was selected based on the ground state absorption of ^1Ph^FluIndz. Both the output of the OPerA Solo and the residual fundamental output were directed into commercial transient absorption spectrometers (Ultrafast Systems Helios, Sarasota, Florida), where the pump pulse was routed through an 850 nm long pass filter (Thorlabs, Newton, NJ) and focused with a 350 mm focal length lens to the sample position. To generate the probe pulse, the residual fundamental output was routed through mechanical delay lines and OPAs (Ultrafast Systems, Sarasota, Florida) that created continua with ranges of 300 to 600 nm and 800 to 1300 nm, respectively. Samples were held in 2 mm or 1 mm quartz cuvettes (FireflySci, Inc., Staten Island, New York), and ultrafast data were corrected with a polynomial to account for temporal chirp. Cyclic voltammetry curves were measured with a C–H Instruments Electrochemical Analyzer (Model CHI602E). All measurements were conducted under an argon atmosphere and taken using a platinum counter electrode, silver pseudo reference electrode, and glassy carbon working electrode. The electrolyte solution used was 0.1 M Bu_4_NPF_6_ in DCM. Ferrocene was used as the reference, taken as 0.00 V and oxidation and reduction potentials of the materials are reported *versus* Fc^+^/Fc in DCM. For all computations, all molecules were first drawn in ChemDraw (20.0.0.41) with all alkyl chains truncated to methyl groups to minimize computational expense, then saved as MDL Molfile. The geometries of those molecules were then optimized with the MMFF94 force field using Avogadro (1.2.0). All single bonds between rings were confirmed to have a dihedral angle between 0 and 90° to avoid parallel or perpendicular relative orientations of adjacent rings (thereby avoiding local minima conformations). Then, sequential geometry optimizations were performed by DFT using Gaussian 16 (ref. 66) employing the default convergence criteria, numerical integrations grids, and implicit solvation parameters with the B3LYP^60,61^ functional and the following basis sets: first 3-21G, then 6-31G(d,p),^85,86^ and finally 6-311G(d,p)^59^ with a dichloromethane polarizable continuum model.^62–65^ Harmonic vibrational frequency computations confirmed that all optimized structures correspond to minima. After generating the optimized geometries, TD-DFT was performed with the B3LYP functional and 6-311G(d,p) basis set to compute the vertical transition energies and oscillator strengths. The aromatic character associated with each optimized structure was investigated using Nucleus-Independent Chemical Shifts (NICS^87^). The gauge-independent atomic orbital (GIAO^88–91^) method was used to compute the B3LYP/6-311G(d,p) magnetic shielding tensors at points 1.0 Å above and below the geometric center of each ring A–G (NICS_ZZ_(±1)). See ESI† for further NICS & crystallographic information. The Crystallographic Information File (CIF) for the crystal reported in this manuscript has been deposited with the Cambridge Crystallographic Data Centre (CCDC) with deposition number 2329750. Stability studies were performed using a G2V Optics Inc. Pico Solar Simulator calibrated to 1 sun intensity as the light source. Dye concentration was adjusted so the absorbance value of the lowest energy feature was measured to be 1.0 in DCM solution in a 1 cm path length cuvette. The samples were monitored at regular time intervals over 24 hours and each study was performed in triplicate. Error bars are given as standard deviation of the remaining absorbance at each time point over the three trials.

## Conclusions

A series of four FluIndz dyes varying in the indolizine donor used were designed, synthesized, and their molecular properties studied. The FluIndz dyes have *λ*_abs_ located in the SWIR and ESWIR and behave most “cyanine-like” in CS_2_ with *λ*_abs_ ranging from 1590–2088 nm. ^1Ph^FluIndz has the greatest *ε* of the series, more than doubling that of ^2Ph^FluIndz (*ε* = 46 000 *versus* 20 000 M^−1^ cm^−1^ in CS_2_), while ^1,7DBA^FluIndz has the lowest *ε* of the series at 18 700 M^−1^ cm^−1^ in CS_2_. The large increase in *ε* observed for ^1Ph^FluIndz is important since antiaromatic dyes have characteristically low *ε* values. Cyclic voltammetry measurements and DFT calculations reveal that donor modifications within the FluIndz series primarily impact *E*_S+/S_ and HOMO energy levels. As electron density increases in the indolizine donor from 1Ph ≈ 2Ph < 7DMA < 1,7DBA, *E*_S+/S_ shifts to more negative potentials and the HOMO energy level increases. Core modifications primarily impact *E*_S+/S*_ and *E*_S/S−_ and LUMO energy levels, where both *E*_S+/S*_ and *E*_S/S−_ shift to more positive potentials and LUMO energy levels decrease from ^tol^RosIndz > SiRos1300 > ^2Ph^FluIndz. These trends in electrochemical and molecular orbital energy levels lead to smaller *E*^opt^_g_ and *E*^H−L^_g_ energy gaps producing the longer wavelength absorptions observed. TD-DFT computational analysis of vertical transitions corroborates the trends observed in maximum *λ*_abs_ where the lowest energy feature shifts to lower energy with increased electron density in the indolizine donor across the FluIndz series 1Ph ≈ 2Ph > 7DMA > 1,7DBA. Core modifications also shift VT and maximum *λ*_abs_ to lower energies from ^tol^RosIndz > SiRos1300 > ^2Ph^FluIndz. NICS analysis demonstrates how core modifications affect aromaticity in the central ring of the chromophore core. In the ground state, ^2Ph^FluIndz is antiaromatic while ^tol^RosIndz is aromatic. In the excited state, Baird's rule is observed where ^2Ph^FluIndz becomes more aromatic while ^tol^RosIndz becomes more antiaromatic. XRD crystallography of ^1Ph^FluIndz reveals unequivocal determination of the chromophore structure and existence as a single molecule. Photostability studies of the FluIndz dyes in DCM solution reveal that ^1Ph^FluIndz is remarkably more photostable than the other analogues which contain a phenyl group at the 2-position of the indolizine, with ∼95% absorption remaining after 24 hours of 1 sun exposure and >90% absorption over an additional week of ambient exposure. EPR spectroscopy reveals that the FluIndz dyes exist to some extent as diradicals and demonstrates how the diradical triplet population in ^2Ph^FluIndz increases with increased temperature. Furthermore, these dyes exhibit lower-than-expected spin counts that are attributed to the “biradical paradox.” Overall, these materials are exceptionally low energy absorbing small molecule organic chromophores and exhibit unique properties that could be exploited in infrared optoelectronic sensing applications. This study provides a rational design framework to influence the future development of ESWIR absorbing organic molecules.

## Data availability

The data underlying this study are available in the published article and its ESI.† Crystallographic data reported herein was deposited with the Cambridge Crystallographic Data Centre (CCDC) with deposition number 2329750.

## Author contributions

Conceptualization: WEM, MAS, JHD; formal analysis: WEM, MAS, MRT, AJM, NAK, CRB, KMC, SRP, GST, and JHD; funding acquisition: JHD, NIH, and GST; investigation: WEM, MAS, MRT, AJM, NAK, CRB, KMC, and SRP; methodology: WEM, MAS, MRT, NAK, KMC, and SRP; project administration: JHD, NIH, and GST; writing – original draft: WEM; writing – review & editing: WEM, MAS, and JHD. All authors have read and agreed to the final version of this manuscript.

## Conflicts of interest

There are no conflicts to declare.

## Supplementary Material

SC-015-D4SC00733F-s001

SC-015-D4SC00733F-s002
