# Supplementary material for: Extended shortwave infrared absorbing antiaromatic fluorenium-indolizine chromophores
Source: Chem Sci. 2024 Jul 12;15(31):12349–60. doi: 10.1039/d4sc00733f (PMC11304523; doi:10.1039/d4sc00733f)
Supplement: SC-015-D4SC00733F-s001 [file SC-015-D4SC00733F-s001.pdf]

Electronic Supplementary Material (ESI) for Chemical Science.
This journal is © The Royal Society of Chemistry 2024

Supplementary Information

Extended Shortwave Infrared Absorbing Antiaromatic Fluorenium-Indolizine Chromophores

William E. Meador,[a‡] Matthew A. Saucier,[a‡] Max R. Tucker,[a] Nicholas A. Kruse,[a] Alexander J. Mobley, [a] Connor R. Brower,[a] Sean R. Parkin,[b] Kensha M. Clark,[a] Nathan I. Hammer,[a] Gregory S. Tschumper,[a] and Jared H. Delcamp[a,c]*

[a] University of Mississippi, Department of Chemistry and Biochemistry, Coulter Hall, University, MS 38677, USA
[b] Department of Chemistry, University of Kentucky, Lexington, Kentucky 40506, United States
[c] Present Address: Air Force Research Laboratory, Materials and Manufacturing Directorate (RXNC), 2230 Tenth Street B655, Wright-Patterson AFB, OH 45433, USA
[‡] These authors contributed equally to this work

Corresponding author email: delcamp@olemiss.edu

TABLE OF CONTENTS

Photophysical Measurements 3
Electrochemical Measurements. 10
Computational Data. 13
Crystallographic Data. 58
Paramagnetic Experiments. 73
Synthesis (procedures and characterization data) 80
NMR & HRMS Spectra. 87
References 120

PHOTOPHYSICAL MEASUREMENTS

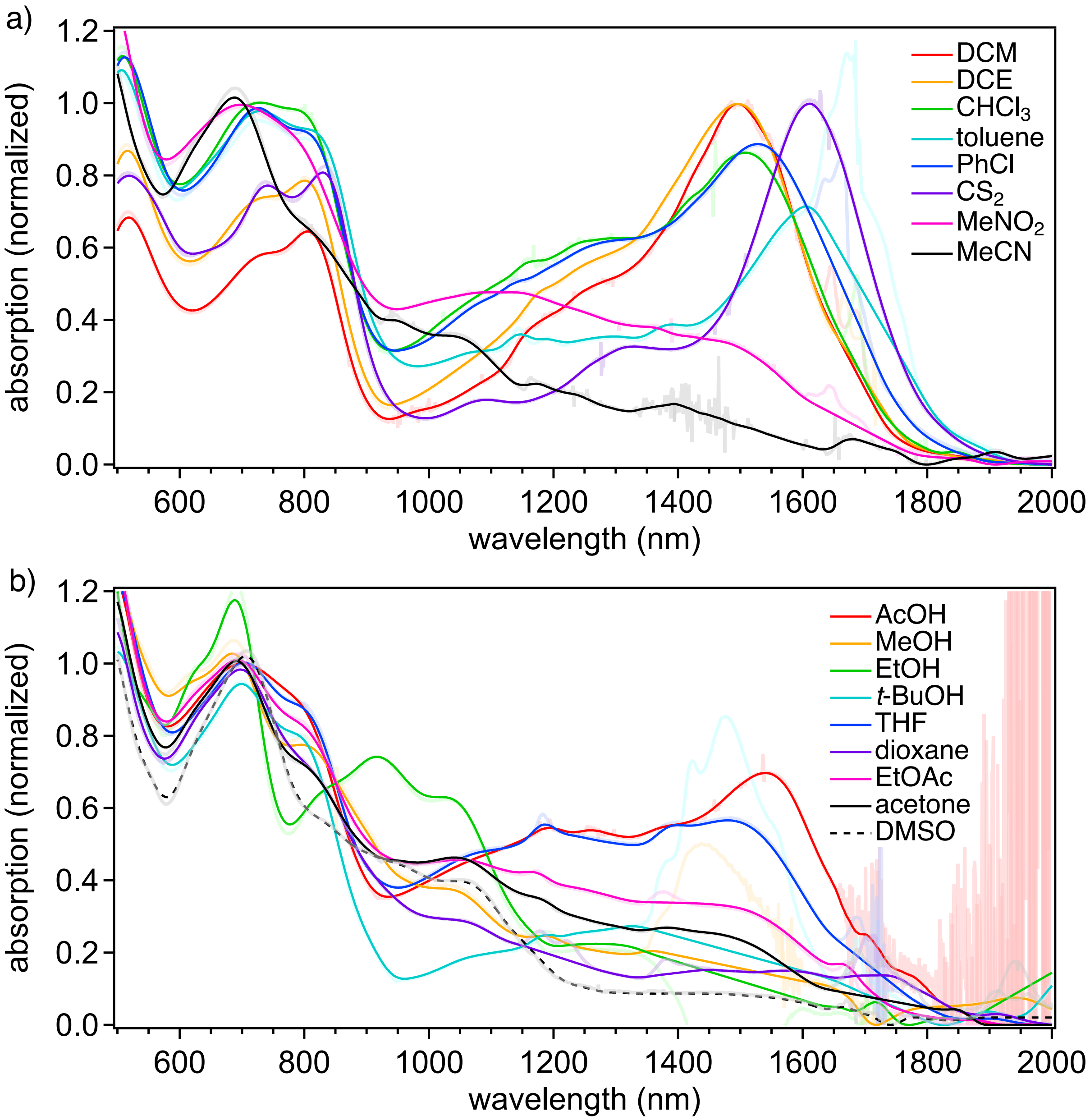

Figure S1. Absorption spectra of **[2Ph]FluIndz** in various solvents. The absorption spectra are normalized to most intense NIR peak (>700 nm) in each spectrum. Raw spectra are included as the corresponding faded color spectrum behind each smoothed/solvent subtracted spectrum.

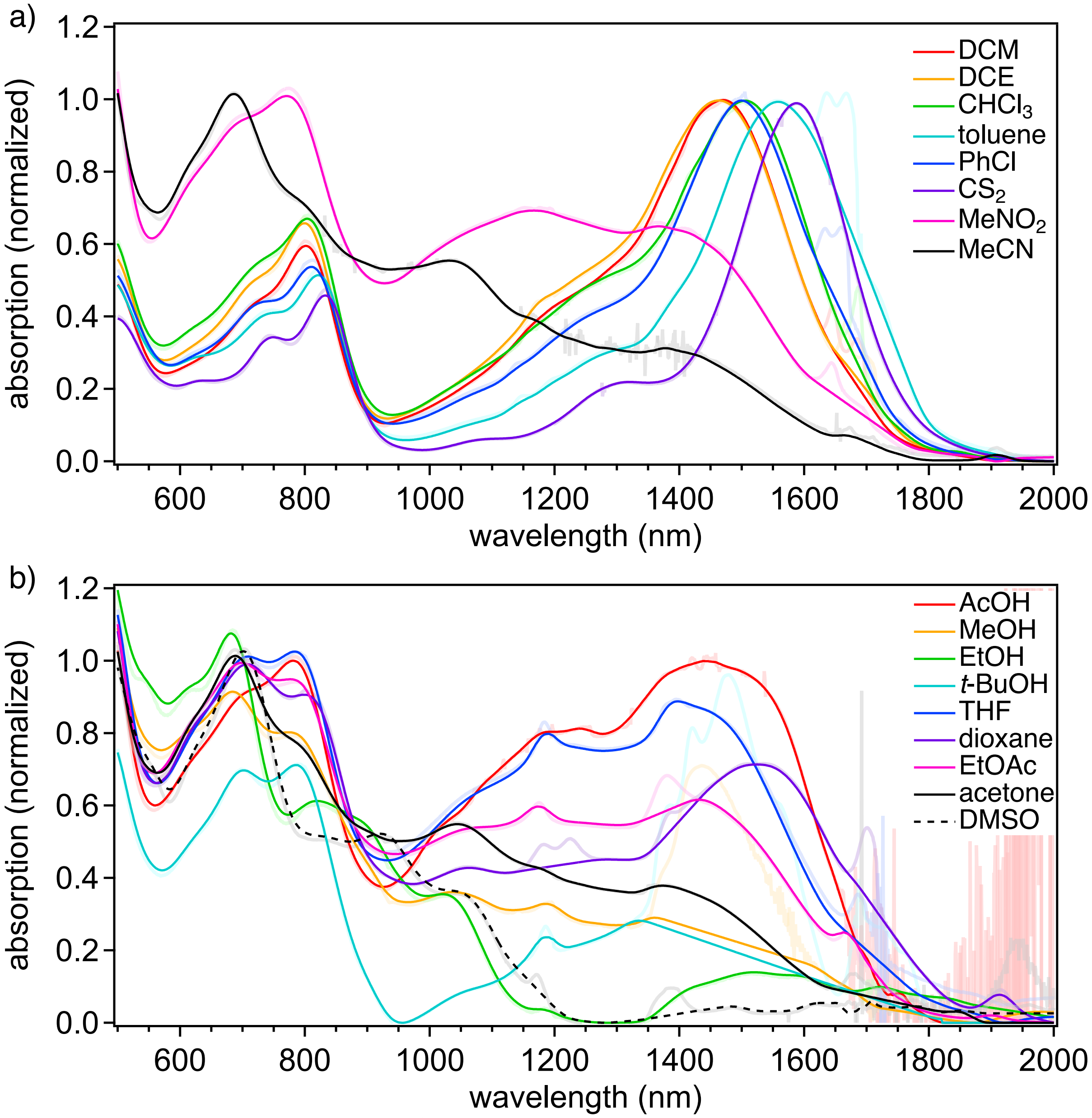

Figure S2. Absorption spectra of **^{1Ph}FluIndz** in various solvents. The absorption spectra are normalized to most intense NIR peak (>700 nm) in each spectrum. Raw spectra are included as the corresponding faded color spectrum behind each smoothed/solvent subtracted spectrum.

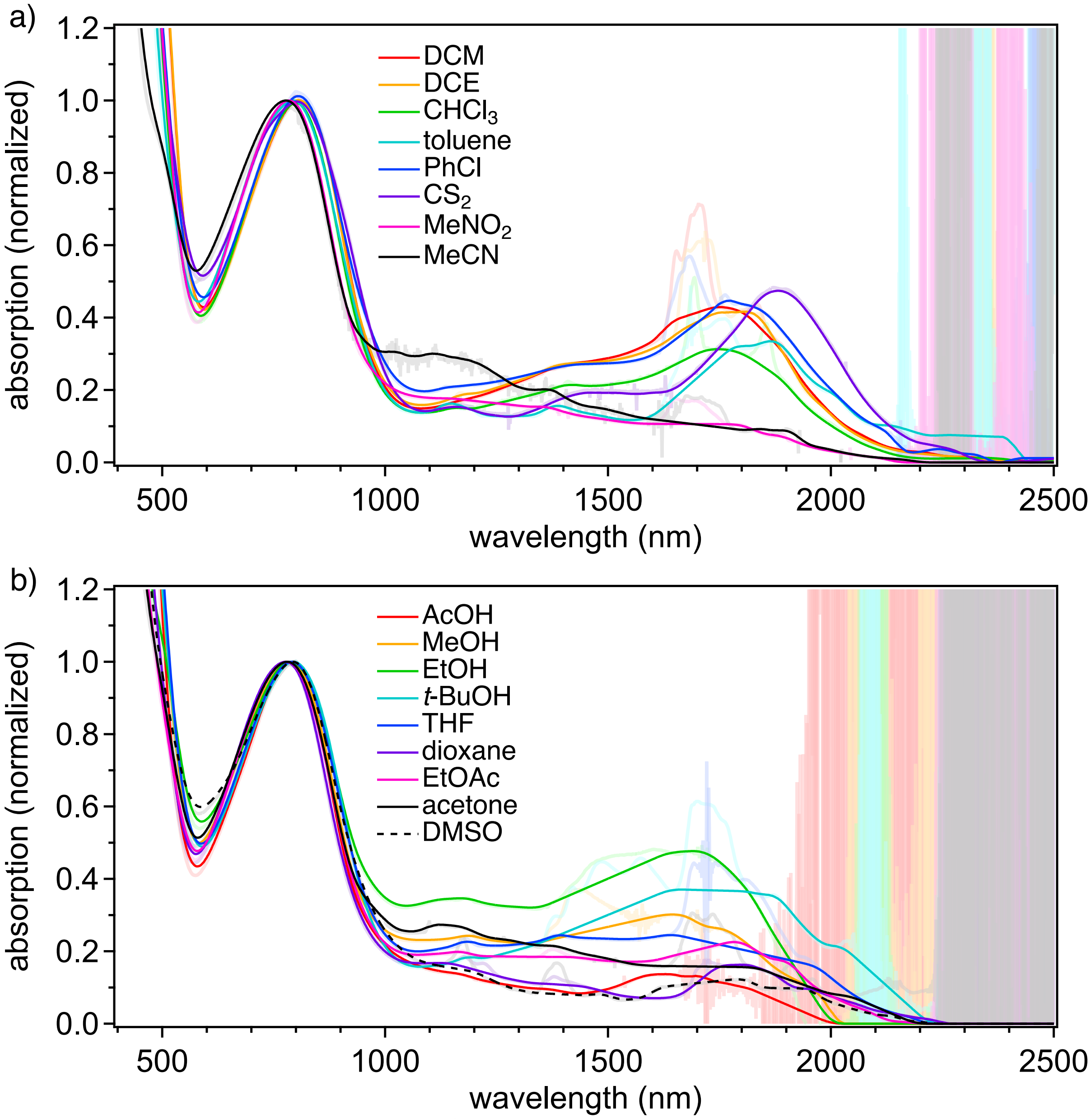

Figure S3. Absorption spectra of [7DMA]**FluIndz** in various solvents. The absorption spectra are normalized to most intense NIR peak (>700 nm) in each spectrum. Raw spectra are included as the corresponding faded color spectrum behind each smoothed/solvent subtracted spectrum.

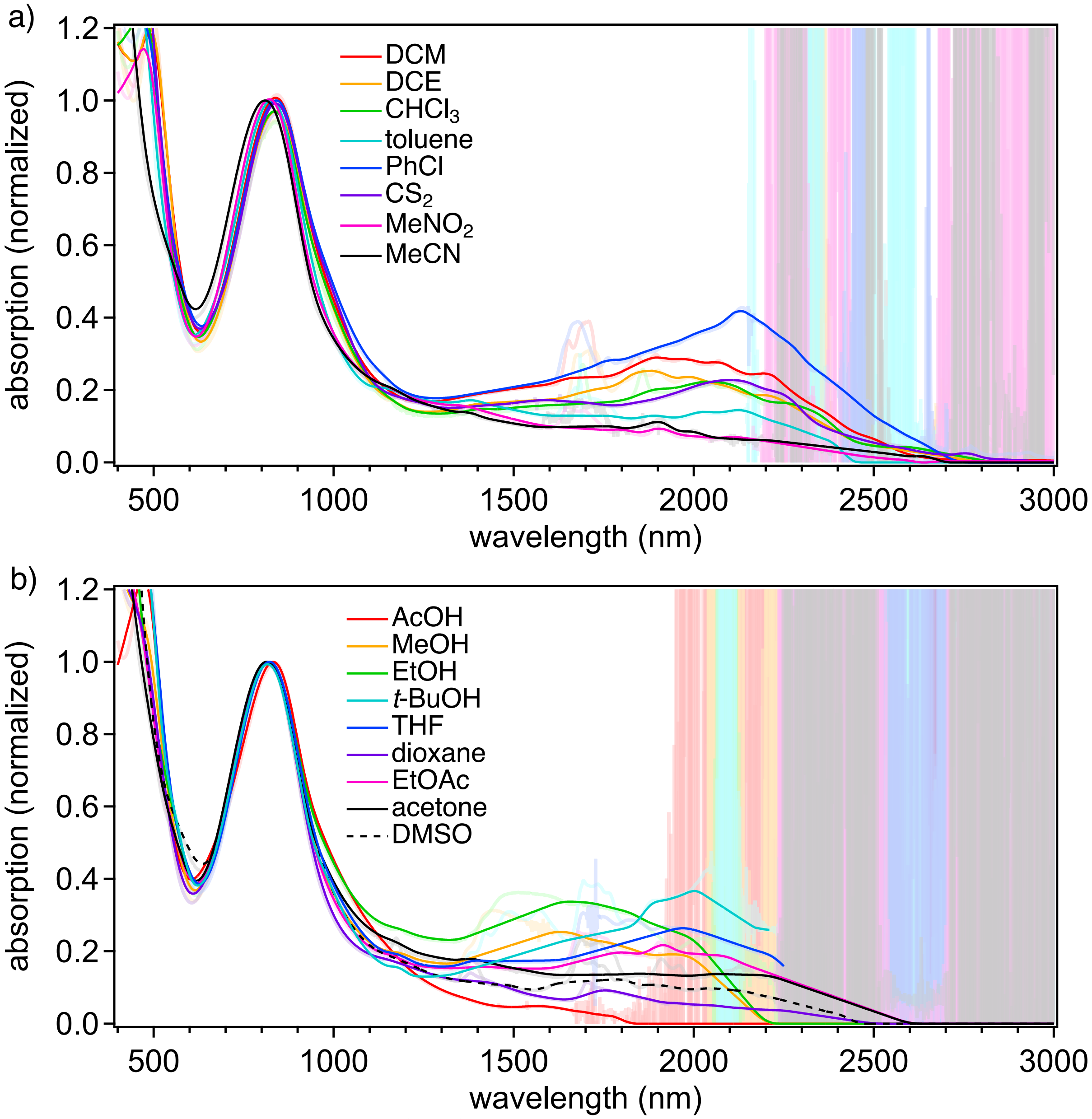

Figure S4. Absorption spectra of [1,7DBA]**FluIndz** in various solvents. The absorption spectra are normalized to most intense NIR peak (>700 nm) in each spectrum. Raw spectra are included as the corresponding faded color spectrum behind each smoothed/solvent subtracted spectrum. Some spectra are truncated at the longest wavelength where dye absorption could be distinguished from solvent absorption.

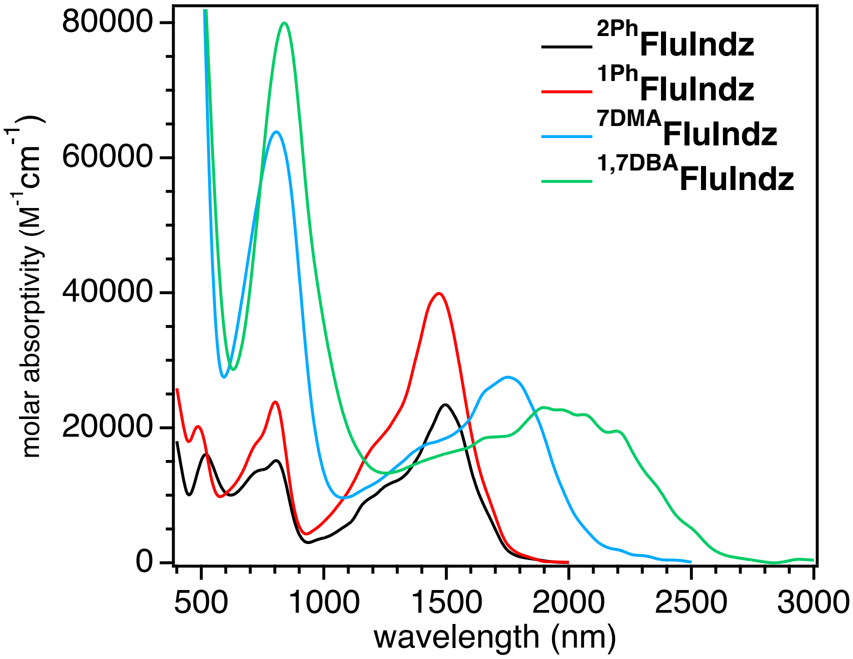

Figure S5. Molar absorptivity of FluIndz dyes in DCM.

Table S1. Molar absorptivity (in M^{-1} cm^{-1}) by peak wavelength in DCM.

Dye	highest energy peak	middle peak	lowest energy peak
^{2Ph}**FluIndz**	15,400 (λ = 524 nm)	14,900 (λ = 816 nm)	23,400 (λ = 1500 nm)
^{1Ph}**FluIndz**	19,700 (λ = 498 nm)	23,500 (λ = 810 nm)	39,900 (λ = 1470 nm)
^{7DMA}**FluIndz**	112,300 (λ = 475 nm)	63,400 (λ = 819 nm)	27,500 (λ = 1756 nm)
^{1,7DBA}**FluIndz**	93,300 (λ = 495 nm)	79,600 (λ = 849 nm)	23,000 (λ = 1956 nm)

Table S2. Molar absorptivity (in M^{-1} cm^{-1}) by peak wavelength in CS_2.

Dye	highest energy peak	middle peak	lowest energy peak
^{2Ph}**FluIndz**	14,900 (λ = 534 nm)	15,700 (λ = 733 nm)<br>17,400 (λ = 796 nm)	20,000 (λ = 1620 nm)
^{1Ph}**FluIndz**	18,300 (λ = 508 nm)	21,200 (λ = 840 nm)	46,000 (λ = 1590 nm)
^{7DMA}**FluIndz**	139,900 (λ = 443 nm)	68,900 (λ = 777 nm)	33,000 (λ = 1882 nm)
^{1,7DBA}**FluIndz**	120,200 (λ = 465 nm)	81,300 (λ = 845 nm)	18,700 (λ = 2088 nm)

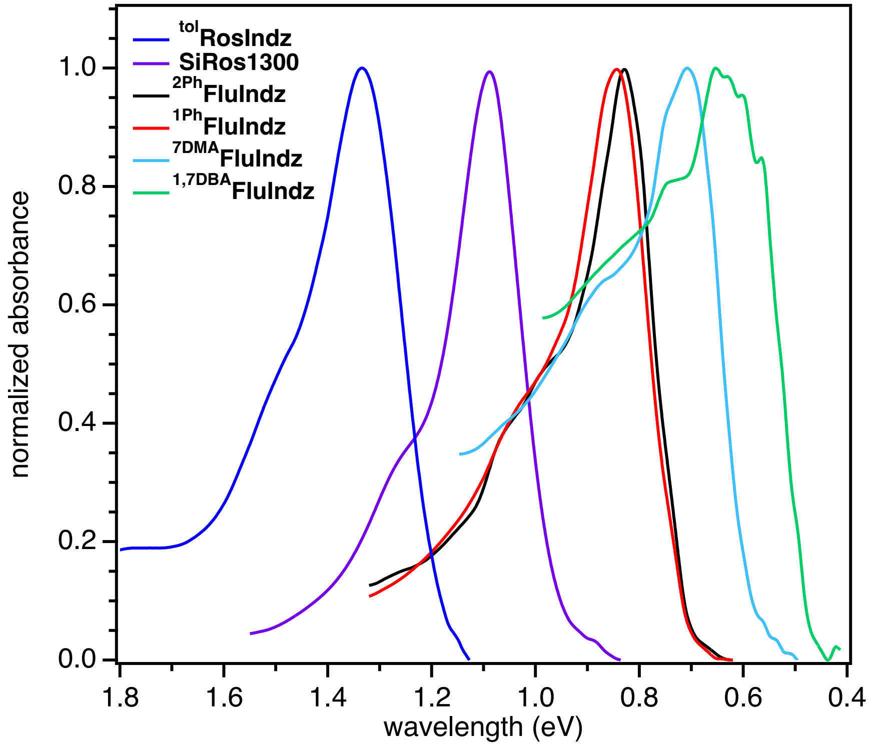

Figure S6. Normalized absorption spectrum of the lowest energy peak of **^{tol}RosIndz**, **SiRos1300**, and all 4 FluIndz derivatives in DCM plotted in a linear energy scale (eV).

Table S3. Comparison of existing inorganic and organic SWIR/ESWIR materials. Note: none of the previous work cited in the category "organic small molecules" (the category that the FluIndz dyes fall under) have reported use in infrared photodetector devices.

material	*intended application*	*comparison to FluIndz dyes*	*references*
$NaErF_4$ nanoparticles	biological imaging	no SWIR/ESWIR absorption, rare and expensive Er	4
InGaAs	SWIR camera	expensive precious metal In, toxic As ions	5, 6, 7, 8
HgCdTe	ESWIR photodetector	costly and difficult manufacturing process, toxic Hg and Cd ions	6, 7, 8, 10, 11
PbS colloidal quantum dots	SWIR photodetector, biological imaging	toxic Pb ions	9, 10, 11
organic polymers	SWIR photodetector, biological imaging	minimal absorption at wavelengths >1700 nm (ESWIR), generally not air-stable	19, 20, 21, 22
organic small molecules	biological imaging, or none given / fundamental studies	minimal absorption at wavelengths >1400 nm, longer wavelength molecules are generally not air-stable	12, 13, 14, 15, 16, 17, 18

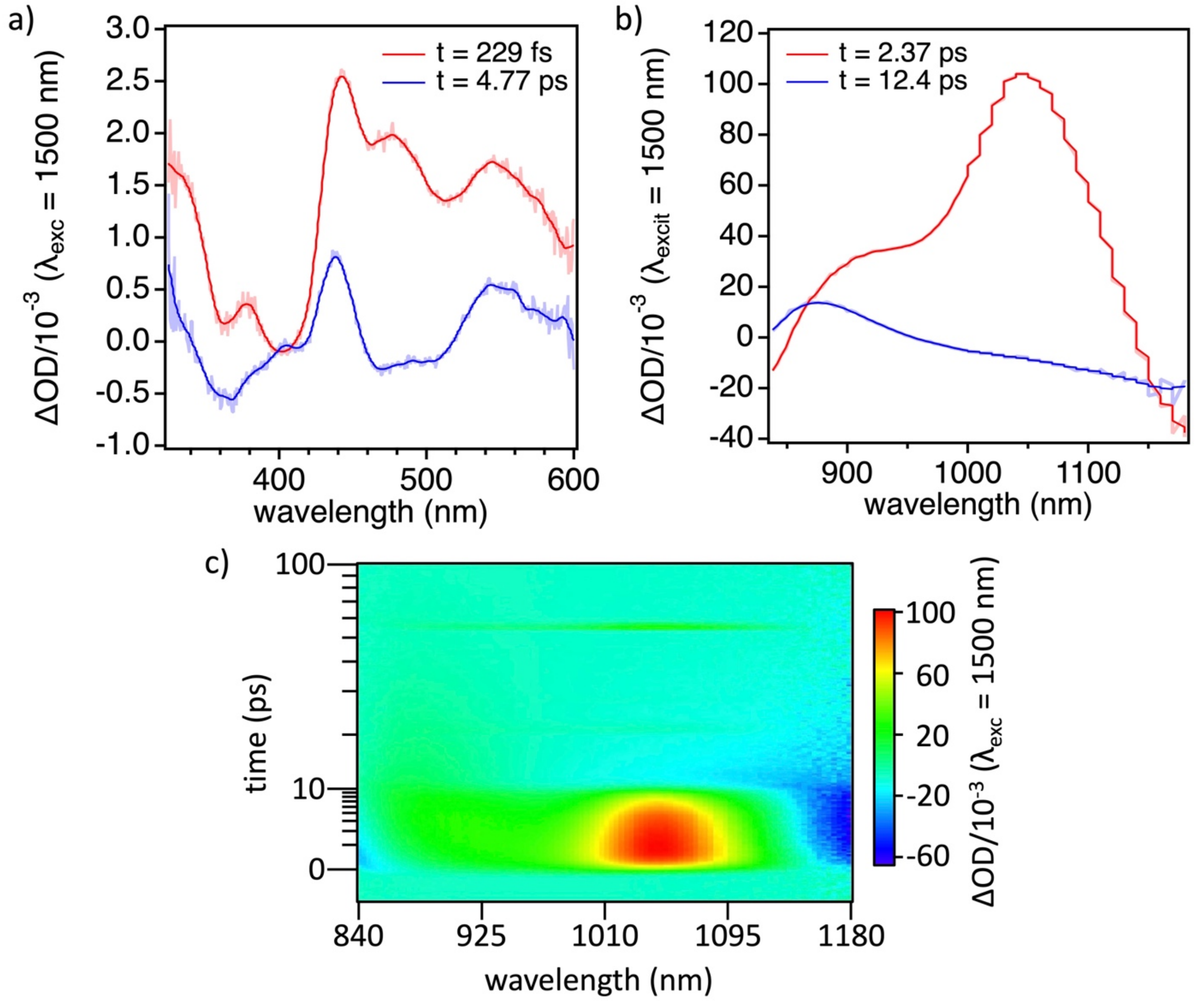

Figure S7. Excited state absorption spectra of **^{1Ph}FluIndz** in the (a) UV-vis and (b) NIR and SWIR regions at the maximum signals in time and (c) three-dimensional differential absorption spectrum in the NIR region. UV-vis transient absorption spectra taken in DCM with 0.1 mM dye using a 2 mm path-length cuvette. NIR transient absorption spectra taken in DCM with 1 mM dye using a 1 mm path-length cuvette.

ELECTROCHEMICAL MEASUREMENTS

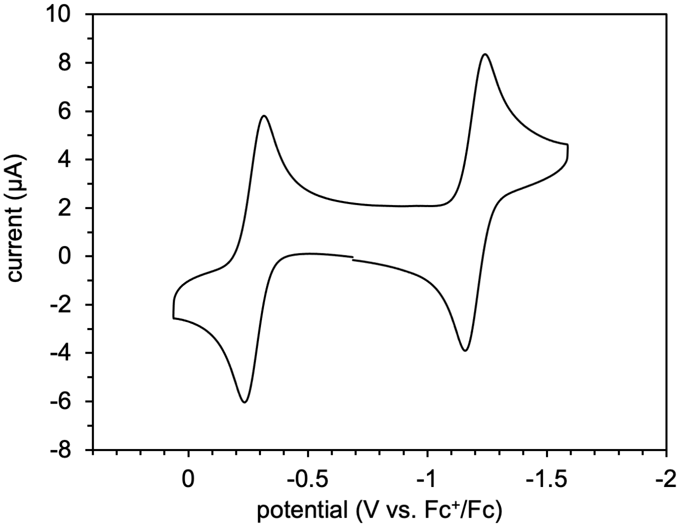

Figure S8. Cyclic voltammogram of **^{2Ph}FluIndz** in DCM referenced to Fc^{+}/Fc. Conditions were DCM with a 0.1 M NBu_4PF_6 supporting electrolyte, glassy carbon working electrode, Pt counter electrode, and Ag pseudo reference electrode. Positive initial scan direction.

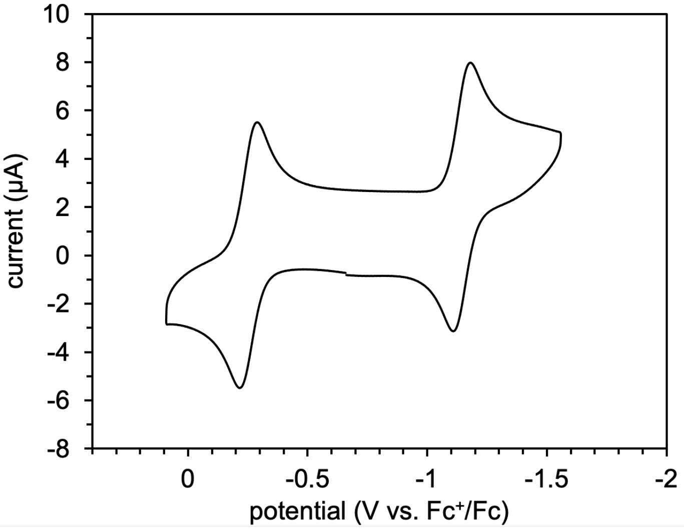

Figure S9. Cyclic voltammogram of **^{1Ph}FluIndz** in DCM referenced to Fc^{+}/Fc. Conditions were DCM with a 0.1 M NBu_4PF_6 supporting electrolyte, glassy carbon working electrode, Pt counter electrode, and Ag pseudo reference electrode. Positive initial scan direction.

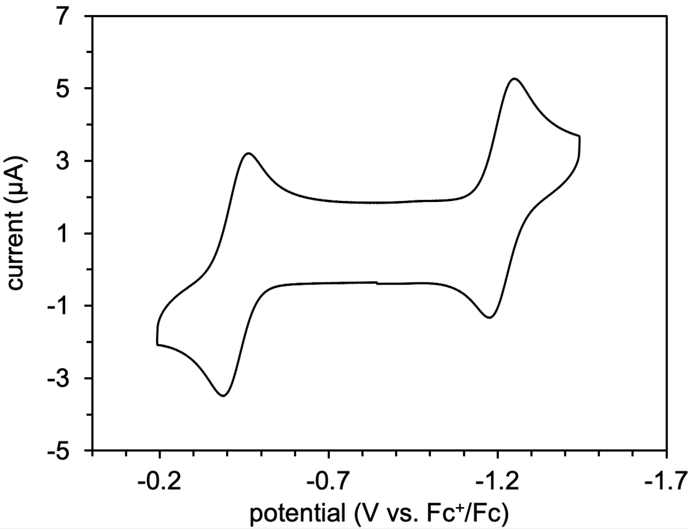

Figure S10. Cyclic voltammogram of [7DMA]**FluIndz** in DCM referenced to Fc^+/Fc. Conditions were DCM with a 0.1 M NBu_4PF_6 supporting electrolyte, glassy carbon working electrode, Pt counter electrode, and Ag pseudo reference electrode. Positive initial scan direction.

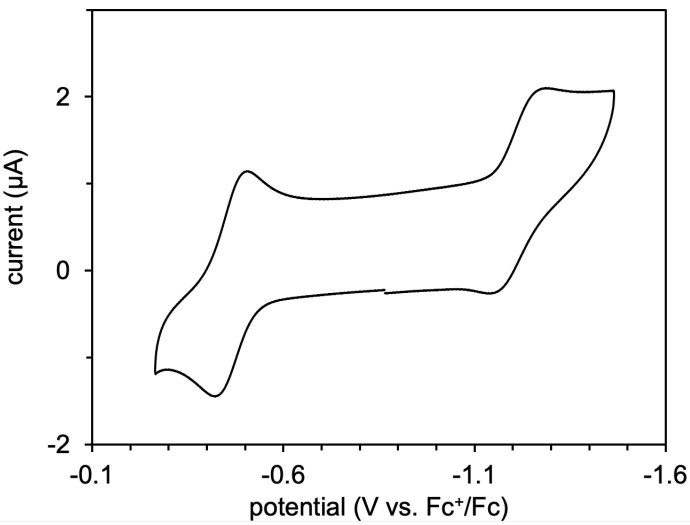

Figure S11. Cyclic voltammogram of [1,7DBA]**FluIndz** in DCM referenced to Fc^+/Fc. Conditions were DCM with a 0.1 M NBu_4PF_6 supporting electrolyte, glassy carbon working electrode, Pt counter electrode, and Ag pseudo reference electrode. Positive initial scan direction.

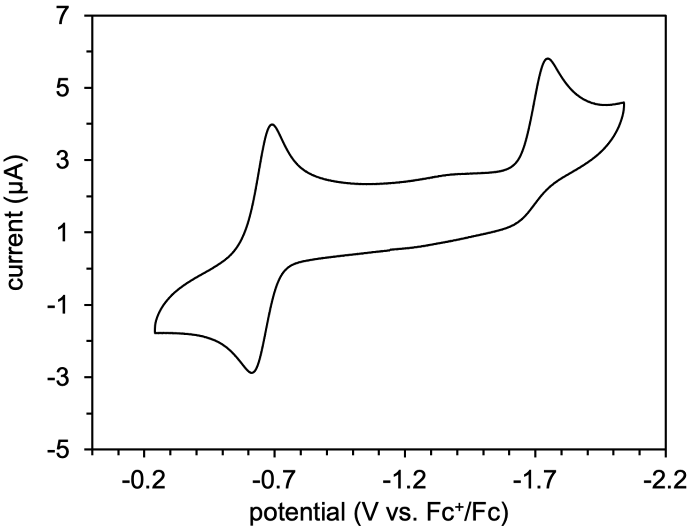

Figure S12. Cyclic voltammogram of **tolRosIndz** in DCM referenced to Fc^+/Fc. Conditions were DCM with a 0.1 M NBu_4PF_6 supporting electrolyte, glassy carbon working electrode, Pt counter electrode, and Ag pseudo reference electrode. Positive initial scan direction.

Table S4. Electrochemical potentials referenced versus Fc^+/Fc at 0.00 V, versus the absolute potential of Fc^+/Fc at 4.8 V,[3] and versus the normal hydrogen electrode (NHE, where Fc^+/Fc = 0.7 V vs. NHE).[1,2]

Dye	Potential	V vs. Fc^+/Fc (at 0.00 V)	V vs. Fc^+/Fc (absolute)	V vs. NHE
**^{tol}RosIndz**	$E_{S+/S}$	-0.65	4.15	0.05
	$E_{S/S-}$	-1.70	3.10	-1.00
	$E_{S+/S*}$	-1.84	2.96	-1.14
**SiRos1300**	$E_{S+/S}$	-0.45	4.35	0.25
	$E_{S/S-}$	-1.68	3.12	-0.98
	$E_{S+/S*}$	-1.37	3.43	-0.67
**^{2Ph}FluIndz**	$E_{S+/S}$	-0.28	4.52	0.42
	$E_{S/S-}$	-1.21	3.59	-0.51
	$E_{S+/S*}$	-1.01	3.79	-0.31
**^{1Ph}FluIndz**	$E_{S+/S}$	-0.25	4.55	0.45
	$E_{S/S-}$	-1.15	3.65	-0.45
	$E_{S+/S*}$	-0.98	3.82	-0.28
**^{7DMA}FluIndz**	$E_{S+/S}$	-0.43	4.37	0.27
	$E_{S/S-}$	-1.21	3.59	-0.51
	$E_{S+/S*}$	-1.03	3.77	-0.33
**^{1,7DBA}FluIndz**	$E_{S+/S}$	-0.47	4.33	0.23
	$E_{S/S-}$	-1.22	3.58	-0.52
	$E_{S+/S*}$	-0.95	3.85	-0.25

COMPUTATIONAL DATA

Table S5. Density functional theory (DFT) and time-dependent DFT (TD-DFT) computational data computed at the B3LYP/6-311G(*d*,*p*) level of theory with dichloromethane implicit solvation. Significant vertical transitions reported with oscillator strength >0.2.

Dye	HOMO (eV)	LUMO (eV)	$E_g^{H\text{-}L}$ (eV)	transit.	orbitals	% Cont.	Vert. Trans. (nm | eV)	Osc. Str.	Dipole (D)
**^{tol}RosIndz**	-5.597	-3.834	1.764	$S_0 \rightarrow S_1$	H → L	100	806 | 1.54	1.023	4.627
				$S_0 \rightarrow S_3$	$H^{-3} \rightarrow L$	99.1	510 | 2.43	0.202	
**SiRos1300**	-5.533	-4.040	1.492	$S_0 \rightarrow S_1$	H → L	99.1	896 | 1.38	1.340	5.857
**^{2Ph}FluIndz**	-5.533	-4.289	1.244	$S_0 \rightarrow S_1$	H → L	96.5	1131 | 1.10	0.521	7.043
				$S_0 \rightarrow S_2$	$H^{-1} \rightarrow L$<br>$H^{-3} \rightarrow L$	94.7<br>4.4	719 | 1.73	0.338	
**^{1Ph}FluIndz**	-5.566	-4.336	1.230	$S_0 \rightarrow S_1$	H → L	97.4	1151 | 1.08	0.631	4.471
				$S_0 \rightarrow S_2$	$H^{-1} \rightarrow L$	97.9	744 | 1.67	0.362	
**^{7DMA}FluIndz**	-5.069	-4.071	0.999	$S_0 \rightarrow S_1$	H → L	98.8	1405 | 0.88	0.553	11.509
				$S_0 \rightarrow S_2$	$H^{-1} \rightarrow L$	98.4	911 | 1.36	0.691	
**^{1,7DBA}FluIndz**	-4.929	-4.067	0.862	$S_0 \rightarrow S_1$	H → L	99.4	1635 | 0.76	0.681	13.287
				$S_0 \rightarrow S_4$	$H^{-3} \rightarrow L$<br>$H^{-1} \rightarrow L$	84.1<br>15.7	918 | 1.35	0.532	

Table S6. Singlet-triplet splitting energies (ΔE_{ST}) described as the adiabatic energy difference (ΔE_{ST}^{ad}) between the total energies of the optimized singlet ground state (S_0) and first triplet state (T_1) and as the vertical transition (ΔE_{ST}^{vert}) obtained via TD-DFT using the S_0 optimized geometry with unrestricted formalism and triplet multiplicity. These data were computed at the B3LYP/6-311G(*d*,*p*) level of theory with dichloromethane implicit solvation. All transitions are given as absolute values resulting in positive values—in all cases, the singlet state is lower in energy than the triplet state. Minimal spin-contamination was observed with $\langle S^2 \rangle$ values ≤2.03 before final annihilation.

Dye	ΔE_{ST}^{ad} (eV | kcal/mol)	ΔE_{ST}^{vert} (eV | kcal/mol)
**^{tol}RosIndz**	0.98 | 22.6	1.05 | 24.3
**SiRos1300**	0.70 | 16.2	0.75 | 17.2
**^{2Ph}FluIndz**	0.40 | 9.2	0.44 | 10.1
**^{1Ph}FluIndz**	0.42 | 9.7	0.46 | 10.6
**^{7DMA}FluIndz**	0.26 | 6.1	0.31 | 7.2
**^{1,7DBA}FluIndz**	0.16 | 3.6	0.23 | 5.2

***tol*RosIndz** *(S_0) Geometry and Frequency Data*
Total Energy: -2112.6946911 Hartrees
The optimized structure did not have any imaginary frequencies ($n_i = 0$).
XYZ Coordinates (in Å):

	Atom	x	y	z
1	C	-1.212333	1.190942	0.035724
2	C	-1.181516	-0.229687	0.094579
3	O	-0.008643	-0.903729	-0.028413
4	C	1.165616	-0.241928	-0.196726
5	C	1.200002	1.179285	-0.228652
6	C	-0.004793	1.903997	-0.115702
7	C	-2.495461	1.800439	0.141471
8	C	-3.628989	1.051519	0.291174
9	C	-3.581285	-0.376654	0.365748
10	C	-2.321614	-0.994453	0.255890
11	C	2.303774	-1.018389	-0.309436
12	C	3.564923	-0.411672	-0.456683
13	C	3.615983	1.018200	-0.476178
14	C	2.484074	1.777774	-0.376723
15	C	4.784037	-1.169662	-0.574112
16	C	-4.801495	-1.122606	0.536314
17	C	-0.003443	3.395873	-0.168861
18	C	0.175786	4.161382	1.000203
19	C	0.166707	5.554764	0.878546
20	C	-0.011512	6.179077	-0.353553
21	C	-0.188042	5.412415	-1.501896
22	C	-0.184749	4.023630	-1.406224
23	C	0.368780	3.512861	2.350258
24	N	4.882430	-2.404493	-1.225366
25	C	6.201567	-2.868111	-1.119335
26	C	6.944943	-1.925626	-0.384525
27	C	6.075611	-0.876114	-0.061355
28	C	3.966963	-3.079104	-2.000708
29	C	4.308362	-4.255636	-2.600829
30	C	5.614722	-4.787047	-2.452608
31	C	6.549775	-4.088076	-1.730295
32	C	-6.094987	-0.859030	0.011776
33	C	-6.965579	-1.882462	0.406560
34	C	-6.221255	-2.777500	1.197719
35	N	-4.900213	-2.312482	1.266988
36	C	-6.570796	-3.953568	1.888601
37	C	-5.634494	-4.607736	2.650261
38	C	-4.325458	-4.073166	2.756448
39	C	-3.982993	-2.939414	2.079451
40	C	-6.454444	0.249104	-0.904421

41	C	6.434075	0.287879	0.782976
42	C	-5.820753	0.388129	-2.147784
43	C	-6.177887	1.416551	-3.015676
44	C	-7.174274	2.323760	-2.656636
45	C	-7.814791	2.192755	-1.425693
46	C	-7.460094	1.162122	-0.557466
47	C	5.791431	0.511946	2.009268
48	C	6.147755	1.593395	2.810488
49	C	7.152243	2.469703	2.400730
50	C	7.801744	2.254343	1.186492
51	C	7.447904	1.170583	0.385176
52	C	8.384530	-2.089157	0.000277
53	C	-8.407098	-2.067600	0.039215
54	H	-2.560011	2.880135	0.114673
55	H	-4.582795	1.548910	0.391600
56	H	-2.212437	-2.069406	0.235296
57	H	2.191961	-2.089754	-0.221548
58	H	4.570908	1.505546	-0.610011
59	H	2.550302	2.856709	-0.426338
60	H	0.301579	6.158072	1.769757
61	H	-0.013108	7.261273	-0.414279
62	H	-0.328252	5.887322	-2.465666
63	H	-0.322085	3.418740	-2.295310
64	H	1.268343	2.890786	2.373036
65	H	-0.475717	2.868209	2.610561
66	H	0.465782	4.268669	3.130276
67	H	2.996722	-2.625652	-2.119304
68	H	3.565907	-4.767007	-3.198340
69	H	5.873468	-5.725566	-2.925790
70	H	7.566203	-4.445734	-1.628899
71	H	-7.589071	-4.312997	1.815927
72	H	-5.894168	-5.512282	3.185084
73	H	-3.581396	-4.547806	3.381627
74	H	-3.010172	-2.483661	2.163149
75	H	-5.052769	-0.319930	-2.437336
76	H	-5.681051	1.506530	-3.975059
77	H	-7.451285	3.124543	-3.332631
78	H	-8.590852	2.893321	-1.139006
79	H	-7.955730	1.070374	0.402305
80	H	5.017305	-0.171569	2.338681
81	H	5.644222	1.748914	3.757916
82	H	7.428721	3.311774	3.024761
83	H	8.584274	2.930225	0.860781
84	H	7.950808	1.012772	-0.562104
85	H	8.589586	-3.106138	0.345734
86	H	8.654596	-1.401671	0.802361

87	H	9.055077	-1.891340	-0.842936
88	H	-8.686955	-1.411618	-0.785430
89	H	-9.072028	-1.842495	0.879986
90	H	-8.609296	-3.097728	-0.267194

***tol*RosIndz** *(T1) Geometry and Frequency Data*
Total Energy: -2112.6587071 Hartrees
The optimized structure did not have any imaginary frequencies (n_i = 0).
XYZ Coordinates (in Å):

	Atom	x	y	z
1	C	-1.219222	1.242485	0.063101
2	C	-1.190992	-0.175570	0.131908
3	O	-0.013527	-0.868874	0.007881
4	C	1.166366	-0.197237	-0.189866
5	C	1.203359	1.220947	-0.246685
6	C	-0.004603	1.965094	-0.117856
7	C	-2.495932	1.848572	0.184580
8	C	-3.642135	1.100476	0.351076
9	C	-3.590801	-0.310189	0.427757
10	C	-2.330782	-0.933525	0.309692
11	C	2.299893	-0.975449	-0.311762
12	C	3.562006	-0.373217	-0.497740
13	C	3.622190	1.038459	-0.545175
14	C	2.482064	1.805693	-0.433608
15	C	4.778554	-1.157859	-0.616719
16	C	-4.813192	-1.075635	0.600167
17	C	-0.000410	3.455472	-0.186143
18	C	0.253762	4.236648	0.960430
19	C	0.245734	5.629801	0.833013
20	C	-0.006011	6.248458	-0.389530
21	C	-0.258380	5.473849	-1.518386
22	C	-0.254968	4.085213	-1.410819
23	C	0.527133	3.596504	2.300769
24	N	4.870371	-2.326300	-1.385611
25	C	6.167595	-2.840457	-1.279658
26	C	6.909054	-1.981928	-0.429848
27	C	6.049278	-0.944521	-0.035991
28	C	3.949929	-2.897044	-2.226386
29	C	4.270727	-4.033066	-2.919949
30	C	5.553607	-4.608616	-2.791029
31	C	6.497166	-4.003673	-1.982517
32	C	-6.075532	-0.910810	-0.013730
33	C	-6.943892	-1.907988	0.457980
34	C	-6.216783	-2.689946	1.390061
35	N	-4.919308	-2.171545	1.467549
36	C	-6.559880	-3.784839	2.189599
37	C	-5.629497	-4.317154	3.061941
38	C	-4.346551	-3.735046	3.156970
39	C	-4.012656	-2.666257	2.369142
40	C	-6.406484	0.094761	-1.049844

41	C	6.394829	0.145560	0.905570
42	C	-5.666568	0.173249	-2.238437
43	C	-5.995472	1.106199	-3.217721
44	C	-7.067835	1.977064	-3.026197
45	C	-7.811555	1.906925	-1.849782
46	C	-7.484996	0.971888	-0.869651
47	C	5.670043	0.326038	2.092423
48	C	6.011739	1.338423	2.984549
49	C	7.081946	2.188249	2.705814
50	C	7.810619	2.017052	1.530377
51	C	7.471295	1.002398	0.637562
52	C	8.328187	-2.213153	-0.021942
53	C	-8.357952	-2.171838	0.052081
54	H	-2.561778	2.928072	0.146467
55	H	-4.595061	1.602958	0.449470
56	H	-2.228325	-2.010947	0.308356
57	H	2.191102	-2.047758	-0.213442
58	H	4.576775	1.523986	-0.697695
59	H	2.553539	2.883831	-0.497220
60	H	0.438582	6.238219	1.710488
61	H	-0.005968	7.330524	-0.457540
62	H	-0.456280	5.943422	-2.475105
63	H	-0.449878	3.475000	-2.286023
64	H	1.442553	2.997458	2.279223
65	H	-0.285807	2.927939	2.598034
66	H	0.641538	4.355283	3.076374
67	H	2.995347	-2.402821	-2.312401
68	H	3.527642	-4.471103	-3.571896
69	H	5.800481	-5.508764	-3.338755
70	H	7.497753	-4.405454	-1.891528
71	H	-7.560753	-4.190427	2.121477
72	H	-5.886888	-5.164298	3.684271
73	H	-3.614107	-4.114450	3.856033
74	H	-3.058115	-2.167605	2.424334
75	H	-4.837006	-0.505726	-2.397593
76	H	-5.416062	1.150741	-4.132883
77	H	-7.322257	2.703840	-3.789069
78	H	-8.645539	2.581258	-1.691942
79	H	-8.060541	0.930353	0.048026
80	H	4.842394	-0.336014	2.318830
81	H	5.444399	1.461286	3.900114
82	H	7.346464	2.976855	3.400829
83	H	8.642780	2.674286	1.305290
84	H	8.035186	0.881998	-0.280452
85	H	8.505002	-3.265437	0.215959
86	H	8.585116	-1.617307	0.853888

87	H	9.024542	-1.941266	-0.823098
88	H	-8.598862	-1.660420	-0.879911
89	H	-9.065118	-1.822991	0.812969
90	H	-8.536042	-3.241357	-0.088059

SiRos1300 *(S_0) Geometry and Frequency Data*
Total Energy: -2446.1716099 Hartrees
The optimized structure did not have any imaginary frequencies ($n_i = 0$).
XYZ Coordinates (in Å):

	Atom	x	y	z
1	C	-6.893250	-2.352256	1.105760
2	C	-7.436651	-3.463054	1.775927
3	C	-6.634564	-4.245373	2.571397
4	C	-5.268775	-3.904466	2.734068
5	C	-4.740450	-2.830762	2.077449
6	N	-5.523370	-2.085377	1.226960
7	C	-5.221738	-0.923354	0.503618
8	C	-6.444736	-0.471042	-0.063806
9	C	-7.468612	-1.355403	0.291696
10	C	-8.907015	-1.326381	-0.127414
11	C	-6.604431	0.679348	-0.984541
12	C	-3.900546	-0.372074	0.378059
13	C	-2.733764	-1.178845	0.295494
14	C	-1.460406	-0.659017	0.150523
15	C	-1.275818	0.764727	0.107299
16	C	-2.453300	1.569436	0.181337
17	C	-3.709153	1.029950	0.295516
18	Si	0.000040	-1.834763	0.000018
19	C	1.460478	-0.659009	-0.150422
20	C	1.275875	0.764715	-0.107295
21	C	0.000008	1.408476	0.000028
22	C	2.733865	-1.178828	-0.295317
23	C	3.900596	-0.372035	-0.378020
24	C	3.709181	1.029976	-0.295654
25	C	2.453307	1.569451	-0.181469
26	C	-0.147681	-2.905890	-1.541349
27	C	0.147662	-2.906099	1.541240
28	C	5.221832	-0.923310	-0.503536
29	C	-0.000010	2.914449	-0.000077
30	N	5.523501	-2.085223	-1.226989
31	C	6.893427	-2.352013	-1.105894
32	C	7.468739	-1.355211	-0.291771
33	C	6.444780	-0.470969	0.063900
34	C	4.740568	-2.830620	-2.077468
35	C	5.268923	-3.904227	-2.734206
36	C	6.634762	-4.245046	-2.571665
37	C	7.436848	-3.462733	-1.776205
38	C	6.604414	0.679308	0.984767
39	C	8.907165	-1.326031	0.127265
40	C	-5.918639	0.722551	-2.206959

41	C	-6.090841	1.793553	-3.079623
42	C	-6.950910	2.839573	-2.746230
43	C	-7.641751	2.805031	-1.536334
44	C	-7.472445	1.732002	-0.663392
45	C	7.472773	1.731794	0.663985
46	C	7.642044	2.804719	1.537059
47	C	6.950822	2.839333	2.746736
48	C	6.090398	1.793489	3.079765
49	C	5.918230	0.722594	2.206966
50	C	-0.094532	3.609769	-1.222499
51	C	-0.092717	5.007418	-1.199872
52	C	-0.000268	5.704503	-0.000256
53	C	0.092332	5.007581	1.199476
54	C	0.094403	3.609968	1.222252
55	C	0.196427	2.879143	2.540937
56	H	-8.492624	-3.671287	1.662606
57	H	-7.043792	-5.101566	3.092048
58	H	-4.627782	-4.478339	3.389383
59	H	-3.718686	-2.513606	2.206812
60	H	-9.054866	-0.648189	-0.968161
61	H	-9.558625	-0.990189	0.686353
62	H	-9.252005	-2.319000	-0.429638
63	H	-2.857404	-2.255615	0.282971
64	H	-2.357795	2.646113	0.163009
65	H	-4.562859	1.689527	0.364931
66	H	2.857534	-2.255593	-0.282692
67	H	4.562873	1.689564	-0.365187
68	H	2.357776	2.646127	-0.163238
69	H	-0.262027	-2.294380	-2.439562
70	H	-1.011290	-3.573251	-1.468812
71	H	0.743939	-3.528152	-1.662140
72	H	0.261678	-2.294726	2.439589
73	H	1.011407	-3.573292	1.468798
74	H	-0.743872	-3.528545	1.661710
75	H	3.718768	-2.513529	-2.206721
76	H	4.627926	-4.478104	-3.389514
77	H	7.044011	-5.101166	-3.092419
78	H	8.492846	-3.670901	-1.662987
79	H	9.252810	-2.318973	0.427642
80	H	9.054654	-0.649277	0.969242
81	H	9.558514	-0.987914	-0.685917
82	H	-5.255810	-0.091811	-2.476030
83	H	-5.556638	1.809001	-4.022902
84	H	-7.083601	3.673546	-3.425837
85	H	-8.312853	3.613604	-1.269702
86	H	-8.005866	1.714524	0.280222

87	H	8.006493	1.714274	-0.279458
88	H	8.313419	3.613156	1.270701
89	H	7.083486	3.673224	3.426448
90	H	5.555891	1.808990	4.022872
91	H	5.255131	-0.091635	2.475771
92	C	-0.196825	2.878829	-2.541092
93	H	-0.164994	5.549326	-2.136565
94	H	-0.000341	6.788620	-0.000272
95	H	0.164582	5.549734	2.136029
96	H	0.268839	3.587142	3.367475
97	H	1.077160	2.231412	2.575930
98	H	-0.676540	2.244300	2.718455
99	H	-0.265542	3.586810	-3.367957
100	H	-1.079780	2.234173	-2.577245
101	H	0.674190	2.240924	-2.717024

SiRos1300 *(T_1) Geometry and Frequency Data*
Total Energy: -2446.1457308 Hartrees
The optimized structure did not have any imaginary frequencies (n_i = 0).
XYZ Coordinates (in Å):

	Atom	x	y	z
1	C	-6.914232	-2.243036	1.258601
2	C	-7.464692	-3.260836	2.044129
3	C	-6.672692	-3.925422	2.960915
4	C	-5.319043	-3.553815	3.115803
5	C	-4.782915	-2.560051	2.341862
6	N	-5.555699	-1.938381	1.395357
7	C	-5.234846	-0.881463	0.531959
8	C	-6.427319	-0.522913	-0.140423
9	C	-7.463485	-1.363599	0.291455
10	C	-8.882018	-1.401730	-0.178434
11	C	-6.543546	0.509568	-1.196099
12	C	-3.901793	-0.323221	0.416551
13	C	-2.742999	-1.126485	0.358338
14	C	-1.461152	-0.604475	0.222565
15	C	-1.278827	0.818165	0.157546
16	C	-2.459723	1.618337	0.213455
17	C	-3.718109	1.074411	0.323176
18	Si	-0.001974	-1.778478	0.109268
19	C	1.453126	-0.614370	-0.106137
20	C	1.278034	0.810171	-0.079714
21	C	0.001779	1.471708	0.039071
22	C	2.727329	-1.146323	-0.269884
23	C	3.886602	-0.350737	-0.391331
24	C	3.712473	1.049743	-0.323275
25	C	2.460677	1.602671	-0.185847
26	C	-0.166378	-2.937496	-1.370278
27	C	0.174183	-2.798077	1.687880
28	C	5.210827	-0.919621	-0.546240
29	C	0.005714	2.976528	0.029854
30	N	5.489283	-2.003543	-1.391365
31	C	6.850448	-2.313359	-1.302189
32	C	7.445047	-1.409130	-0.385695
33	C	6.432816	-0.549437	0.064407
34	C	4.674625	-2.647396	-2.286724
35	C	5.172816	-3.667357	-3.052055
36	C	6.529201	-4.044129	-2.941447
37	C	7.362174	-3.358029	-2.078183
38	C	6.599467	0.513217	1.082896
39	C	8.882258	-1.443161	0.023605
40	C	-5.744672	0.453130	-2.347279

41	C	-5.872665	1.413555	-3.346735
42	C	-6.799521	2.447042	-3.212877
43	C	-7.600507	2.511869	-2.074339
44	C	-7.475330	1.549681	-1.074270
45	C	7.527460	1.545603	0.888302
46	C	7.700330	2.536282	1.852823
47	C	6.951752	2.507933	3.027925
48	C	6.029066	1.482632	3.233903
49	C	5.853235	0.493832	2.270030
50	C	-0.096801	3.673432	-1.191829
51	C	-0.091298	5.071604	-1.178964
52	C	0.013412	5.775120	0.016327
53	C	0.114237	5.082658	1.218349
54	C	0.112037	3.684648	1.244673
55	C	0.224017	2.958250	2.565039
56	H	-8.513403	-3.501939	1.930495
57	H	-7.090672	-4.713899	3.573154
58	H	-4.690595	-4.036941	3.851113
59	H	-3.765749	-2.216637	2.442495
60	H	-8.991807	-0.884819	-1.131928
61	H	-9.554771	-0.918143	0.538688
62	H	-9.229453	-2.430512	-0.304186
63	H	-2.865760	-2.205025	0.370655
64	H	-2.367685	2.694930	0.173268
65	H	-4.577840	1.730600	0.368449
66	H	2.845614	-2.225371	-0.255392
67	H	4.572901	1.700422	-0.412172
68	H	2.374707	2.680384	-0.168427
69	H	-0.287074	-2.375736	-2.300019
70	H	-1.030882	-3.598488	-1.257229
71	H	0.721583	-3.569840	-1.467068
72	H	0.271191	-2.153077	2.564928
73	H	1.057983	-3.441529	1.640186
74	H	-0.696995	-3.444034	1.834273
75	H	3.656243	-2.299979	-2.356669
76	H	4.511724	-4.167163	-3.746490
77	H	6.916940	-4.853537	-3.546220
78	H	8.413122	-3.603307	-1.999726
79	H	9.224116	-2.469520	0.181154
80	H	9.039151	-0.885079	0.946725
81	H	9.527497	-1.001185	-0.743880
82	H	-5.028141	-0.351821	-2.461717
83	H	-5.250753	1.352575	-4.232580
84	H	-6.897316	3.195160	-3.991116
85	H	-8.322322	3.312747	-1.961562
86	H	-8.093387	1.612561	-0.185904

87	H	8.104815	1.579857	-0.028548
88	H	8.418311	3.330684	1.683822
89	H	7.086773	3.278106	3.778609
90	H	5.447602	1.450310	4.148279
91	H	5.140036	-0.304218	2.440234
92	C	-0.213064	2.935087	-2.505146
93	H	-0.170301	5.609566	-2.117802
94	H	0.016343	6.859504	0.011077
95	H	0.196110	5.629257	2.151947
96	H	0.298943	3.667536	3.390960
97	H	1.106506	2.312410	2.593974
98	H	-0.645249	2.319396	2.746631
99	H	-0.287186	3.636934	-3.337469
100	H	-1.097495	2.291649	-2.526752
101	H	0.654091	2.292101	-2.682213

*^{2Ph}**FluIndz** (S_0) Geometry and Frequency Data*
Total Energy: -2076.7636455 Hartrees
The optimized structure did not have any imaginary frequencies ($n_i = 0$).
XYZ Coordinates (in Å):

	Atom	x	y	z
1	C	3.401329	1.110195	0.575959
2	C	3.008936	-0.253394	0.534379
3	C	1.627250	-0.559657	0.302287
4	C	0.725193	0.455962	0.142596
5	C	1.132253	1.826507	0.214806
6	C	2.485977	2.135247	0.427845
7	C	0.000028	2.662803	-0.000070
8	C	-1.132212	1.826521	-0.214923
9	C	-0.725174	0.455971	-0.142693
10	C	-1.627247	-0.559637	-0.302358
11	C	-3.008932	-0.253360	-0.534461
12	C	-3.401305	1.110237	-0.576022
13	C	-2.485935	2.135277	-0.427939
14	C	3.990260	-1.292776	0.673410
15	N	3.742212	-2.524295	1.298110
16	C	4.890359	-3.319273	1.205946
17	C	5.880698	-2.588029	0.512883
18	C	5.333195	-1.340130	0.203931
19	C	2.666987	-2.935099	2.049333
20	C	2.663187	-4.175534	2.621135
21	C	3.779145	-5.034568	2.477319
22	C	4.886953	-4.597111	1.788567
23	C	6.013611	-0.292361	-0.594182
24	C	7.195496	0.299571	-0.128581
25	C	7.850863	1.268488	-0.886254
26	C	7.337669	1.656310	-2.122457
27	C	6.165802	1.068531	-2.598302
28	C	5.508774	0.102628	-1.841161
29	C	-3.990300	-1.292710	-0.673440
30	N	-3.742407	-2.524206	-1.298263
31	C	-4.890567	-3.319148	-1.205927
32	C	-5.880777	-2.587882	-0.512704
33	C	-5.333191	-1.340003	-0.203829
34	C	-2.667348	-2.935021	-2.049721
35	C	-2.663694	-4.175440	-2.621559
36	C	-3.779643	-5.034449	-2.477545
37	C	-4.887309	-4.596973	-1.788578
38	C	-6.013464	-0.292213	0.594378
39	C	-7.195382	0.299775	0.128932
40	C	-7.850610	1.268714	0.886697

41	C	-7.337241	1.656503	2.122839
42	C	-6.165339	1.068669	2.598528
43	C	-5.508449	0.102745	1.841295
44	C	0.000039	4.144161	-0.000077
45	C	-0.006946	4.840040	1.227697
46	C	-0.010723	6.237040	1.203931
47	C	0.000067	6.932952	-0.000089
48	C	0.010844	6.237030	-1.204103
49	C	0.007039	4.840030	-1.227858
50	C	0.030111	4.107754	-2.549693
51	C	-0.030023	4.107775	2.549538
52	C	7.232222	-3.123951	0.153213
53	C	-7.232270	-3.123758	-0.152852
54	H	4.438818	1.349433	0.761153
55	H	1.315636	-1.590048	0.189920
56	H	2.811830	3.167237	0.487159
57	H	-1.315648	-1.590024	-0.189933
58	H	-4.438795	1.349490	-0.761190
59	H	-2.811776	3.167271	-0.487252
60	H	1.860479	-2.231816	2.177789
61	H	1.800352	-4.477836	3.198780
62	H	3.767610	-6.016710	2.931975
63	H	5.772761	-5.212159	1.698162
64	H	7.594610	0.007511	0.836137
65	H	8.761416	1.719982	-0.509126
66	H	7.847874	2.409042	-2.712374
67	H	5.764536	1.359617	-3.562455
68	H	4.602544	-0.355717	-2.219979
69	H	-1.860848	-2.231760	-2.178333
70	H	-1.800982	-4.477748	-3.199386
71	H	-3.768225	-6.016578	-2.932232
72	H	-5.773125	-5.211990	-1.698035
73	H	-7.594631	0.007742	-0.835738
74	H	-8.761191	1.720251	0.509689
75	H	-7.847339	2.409252	2.712827
76	H	-5.763936	1.359731	3.562632
77	H	-4.602189	-0.355641	2.219992
78	H	-0.024433	6.779888	2.142721
79	H	0.000077	8.017100	-0.000093
80	H	0.024563	6.779871	-2.142897
81	H	0.143806	4.810938	-3.375773
82	H	-0.892765	3.544437	-2.715177
83	H	0.856154	3.392807	-2.598891
84	H	0.892870	3.544493	2.715047
85	H	-0.856041	3.392798	2.598725
86	H	-0.143761	4.810963	3.375610

87	H	7.875530	-3.217455	1.034719
88	H	7.736281	-2.467206	-0.555593
89	H	7.157422	-4.116694	-0.299750
90	H	-7.875703	-3.217228	-1.034271
91	H	-7.736207	-2.467002	0.556030
92	H	-7.157444	-4.116509	0.300088

***^{2Ph}FluIndz** (T$_1$) Geometry and Frequency Data*
Total Energy: -2076.7489911 Hartrees
The optimized structure did not have any imaginary frequencies (n_i = 0).
XYZ Coordinates (in Å):

	Atom	x	y	z
1	C	3.393946	1.109594	0.579519
2	C	2.993055	-0.256497	0.537150
3	C	1.626958	-0.559692	0.305432
4	C	0.716335	0.462423	0.139817
5	C	1.128374	1.837506	0.210640
6	C	2.486653	2.138693	0.430926
7	C	-0.000026	2.685796	0.000021
8	C	-1.128402	1.837480	-0.210626
9	C	-0.716335	0.462406	-0.139775
10	C	-1.626936	-0.559731	-0.305382
11	C	-2.993033	-0.256566	-0.537138
12	C	-3.393953	1.109515	-0.579528
13	C	-2.486684	2.138635	-0.430933
14	C	3.987403	-1.292244	0.699421
15	N	3.765058	-2.473435	1.425234
16	C	4.911864	-3.270009	1.361717
17	C	5.879169	-2.585055	0.581090
18	C	5.311424	-1.367578	0.191760
19	C	2.700276	-2.831870	2.211552
20	C	2.714980	-4.027063	2.878425
21	C	3.834155	-4.882650	2.782987
22	C	4.930638	-4.493792	2.037816
23	C	5.956546	-0.368304	-0.691559
24	C	7.178756	0.214601	-0.329471
25	C	7.800151	1.138798	-1.166951
26	C	7.211819	1.490600	-2.380267
27	C	5.998302	0.912725	-2.752457
28	C	5.374709	-0.008252	-1.915562
29	C	-3.987358	-1.292334	-0.699406
30	N	-3.764973	-2.473554	-1.425160
31	C	-4.911767	-3.270145	-1.361640
32	C	-5.879124	-2.585148	-0.581114
33	C	-5.311395	-1.367663	-0.191788
34	C	-2.700153	-2.832017	-2.211415
35	C	-2.714811	-4.027250	-2.878219
36	C	-3.833977	-4.882848	-2.782777
37	C	-4.930497	-4.493964	-2.037672
38	C	-5.956561	-0.368353	0.691457
39	C	-7.178770	0.214510	0.329300
40	C	-7.800209	1.138740	1.166710

41	C	-7.211921	1.490621	2.380024
42	C	-5.998402	0.912793	2.752282
43	C	-5.374766	-0.008219	1.915457
44	C	-0.000046	4.169327	0.000025
45	C	-0.001443	4.871466	1.224660
46	C	-0.002199	6.269089	1.203727
47	C	-0.000089	6.966039	0.000029
48	C	0.002042	6.269092	-1.203670
49	C	0.001329	4.871469	-1.224606
50	C	0.015591	4.139170	-2.546619
51	C	-0.015695	4.139165	2.546672
52	C	7.222046	-3.138808	0.227021
53	C	-7.222025	-3.138881	-0.227107
54	H	4.434301	1.341230	0.763827
55	H	1.310847	-1.590842	0.202670
56	H	2.818469	3.168880	0.487571
57	H	-1.310804	-1.590873	-0.202610
58	H	-4.434313	1.341125	-0.763844
59	H	-2.818522	3.168815	-0.487587
60	H	1.887014	-2.127761	2.282520
61	H	1.861553	-4.293534	3.486510
62	H	3.839361	-5.827649	3.310463
63	H	5.816912	-5.111696	1.978657
64	H	7.635989	-0.047189	0.617944
65	H	8.742391	1.584537	-0.869326
66	H	7.695512	2.208900	-3.032144
67	H	5.537749	1.177202	-3.697494
68	H	4.435278	-0.458655	-2.214169
69	H	-1.886896	-2.127903	-2.282385
70	H	-1.861354	-4.293744	-3.486251
71	H	-3.839150	-5.827875	-3.310203
72	H	-5.816767	-5.111874	-1.978520
73	H	-7.635971	-0.047345	-0.618113
74	H	-8.742450	1.584442	0.869032
75	H	-7.695647	2.208948	3.031846
76	H	-5.537882	1.177332	3.697318
77	H	-4.435335	-0.458586	2.214117
78	H	-0.008097	6.812041	2.142880
79	H	-0.000106	8.050433	0.000031
80	H	0.007922	6.812046	-2.142822
81	H	0.119961	4.841344	-3.375346
82	H	-0.906439	3.571382	-2.702921
83	H	0.842251	3.425302	-2.599696
84	H	0.906330	3.571365	2.702961
85	H	-0.842363	3.425305	2.599758
86	H	-0.120048	4.841338	3.375401

87	H	7.908414	-3.100221	1.080012
88	H	7.675219	-2.574128	-0.587487
89	H	7.148077	-4.185264	-0.081517
90	H	-7.908349	-3.100301	-1.080134
91	H	-7.675234	-2.574184	0.587369
92	H	-7.148080	-4.185333	0.081449

^{1Ph}***FluIndz*** *(S_0) Geometry and Frequency Data*
Total Energy: -1998.1162419 Hartrees
The optimized structure did not have any imaginary frequencies (n_i = 0).
XYZ Coordinates (in Å):

	Atom	x	y	z
1	C	-5.113372	-3.902034	1.087078
2	C	-4.027504	-4.493123	1.691694
3	C	-2.878076	-3.719966	1.976216
4	C	-2.821863	-2.408069	1.601659
5	N	-3.873589	-1.836444	0.927109
6	C	-5.066768	-2.545671	0.727682
7	C	-4.040924	-0.520555	0.464191
8	C	-5.368907	-0.429717	0.010747
9	C	-6.012446	-1.655144	0.146719
10	C	-3.028246	0.491614	0.393458
11	C	-1.638719	0.185713	0.222100
12	C	-0.731562	1.203375	0.107956
13	C	-1.140961	2.573476	0.161133
14	C	-2.504192	2.882339	0.303591
15	C	-3.422571	1.856628	0.412465
16	C	0.731553	1.203370	-0.107912
17	C	1.140956	2.573466	-0.161111
18	C	-0.000004	3.409978	0.000008
19	C	1.638711	0.185704	-0.222043
20	C	3.028242	0.491613	-0.393412
21	C	3.422564	1.856617	-0.412460
22	C	2.504180	2.882329	-0.303587
23	C	4.040925	-0.520573	-0.464139
24	N	3.873551	-1.836494	-0.926989
25	C	5.066748	-2.545711	-0.727611
26	C	6.012454	-1.655160	-0.146745
27	C	5.368924	-0.429726	-0.010767
28	C	2.821764	-2.408162	-1.601429
29	C	2.877962	-3.720075	-1.975927
30	C	4.027418	-4.493204	-1.691461
31	C	5.113324	-3.902090	-1.086953
32	C	-7.389583	-1.970858	-0.264828
33	C	7.389618	-1.970857	0.264716
34	C	0.000001	4.890658	-0.000001
35	C	0.083058	5.586666	-1.225327
36	C	0.076543	6.983557	-1.201655
37	C	0.000016	7.679431	-0.000014
38	C	-0.076519	6.983568	1.201633
39	C	-0.083050	5.586677	1.225316
40	C	-0.152805	4.855801	2.546415

41	C	0.152803	4.855775	-2.546418
42	C	-7.905993	-1.440155	-1.458735
43	C	-9.213712	-1.707015	-1.851649
44	C	-10.034073	-2.513288	-1.062993
45	C	-9.536625	-3.043701	0.126136
46	C	-8.229787	-2.773974	0.524013
47	C	8.229756	-2.774022	-0.524144
48	C	9.536623	-3.043736	-0.126353
49	C	10.034166	-2.513258	1.062709
50	C	9.213871	-1.706936	1.851383
51	C	7.906123	-1.440091	1.458554
52	H	-6.014729	-4.463476	0.885040
53	H	-4.059294	-5.538457	1.970208
54	H	-2.035056	-4.147947	2.501041
55	H	-1.985012	-1.771134	1.835773
56	H	-5.800704	0.459508	-0.420975
57	H	-1.316837	-0.841349	0.117252
58	H	-2.834207	3.914100	0.337923
59	H	-4.468629	2.096506	0.549846
60	H	1.316840	-0.841356	-0.117198
61	H	4.468623	2.096488	-0.549857
62	H	2.834190	3.914091	-0.337941
63	H	5.800748	0.459517	0.420895
64	H	1.984894	-1.771246	-1.835492
65	H	2.034906	-4.148092	-2.500664
66	H	4.059192	-5.538551	-1.969927
67	H	6.014695	-4.463524	-0.884954
68	H	0.129960	7.526488	-2.138927
69	H	0.000023	8.763554	-0.000019
70	H	-0.129930	7.526508	2.138900
71	H	-0.097157	5.560289	3.377273
72	H	0.667690	4.140948	2.654532
73	H	-1.084702	4.292545	2.648477
74	H	-0.667699	4.140929	-2.654525
75	H	1.084694	4.292510	-2.648476
76	H	0.097159	5.560255	-3.377284
77	H	-7.269665	-0.830017	-2.089601
78	H	-9.590366	-1.291060	-2.779230
79	H	-11.051625	-2.723728	-1.371126
80	H	-10.170180	-3.660852	0.753038
81	H	-7.872135	-3.163116	1.469678
82	H	7.872029	-3.163216	-1.469759
83	H	10.170125	-3.660927	-0.753269
84	H	11.051741	-2.723687	1.370774
85	H	9.590599	-1.290931	2.778911
86	H	7.269848	-0.829914	2.089435

***[1Ph]FluIndz** (T1) Geometry and Frequency Data*
Total Energy: -1998.1008436 Hartrees
The optimized structure did not have any imaginary frequencies (n_i = 0).
XYZ Coordinates (in Å):

	Atom	x	y	z
1	C	-5.141118	-3.863864	1.239571
2	C	-4.066804	-4.415770	1.910514
3	C	-2.924750	-3.630009	2.170703
4	C	-2.858283	-2.342513	1.712238
5	N	-3.897852	-1.816763	0.989554
6	C	-5.084200	-2.537489	0.800408
7	C	-4.043544	-0.533003	0.432049
8	C	-5.349004	-0.467164	-0.071174
9	C	-6.009535	-1.678526	0.130215
10	C	-3.017113	0.478439	0.361996
11	C	-1.640961	0.176810	0.205435
12	C	-0.723400	1.200927	0.097154
13	C	-1.138582	2.575465	0.143637
14	C	-2.508193	2.876062	0.279058
15	C	-3.419712	1.845798	0.378698
16	C	0.723429	1.200929	-0.097112
17	C	1.138605	2.575469	-0.143609
18	C	0.000010	3.424256	0.000012
19	C	1.640995	0.176816	-0.205387
20	C	3.017144	0.478451	-0.361947
21	C	3.419738	1.845811	-0.378680
22	C	2.508214	2.876072	-0.279045
23	C	4.043564	-0.532998	-0.432006
24	N	3.897804	-1.816782	-0.989427
25	C	5.084158	-2.537516	-0.800354
26	C	6.009554	-1.678543	-0.130257
27	C	5.349048	-0.467169	0.071154
28	C	2.858154	-2.342547	-1.711980
29	C	2.924560	-3.630061	-2.170402
30	C	4.066633	-4.415824	-1.910298
31	C	5.141018	-3.863909	-1.239474
32	C	-7.373737	-2.007763	-0.295872
33	C	7.373781	-2.007784	0.295748
34	C	0.000007	4.907466	0.000004
35	C	0.089311	5.609510	-1.221581
36	C	0.086756	7.007083	-1.200560
37	C	0.000001	7.704004	-0.000013
38	C	-0.086752	7.007097	1.200542
39	C	-0.089300	5.609525	1.221580
40	C	-0.171002	4.877887	2.541547

41	C	0.171017	4.877856	-2.541539
42	C	-7.873458	-1.492893	-1.505632
43	C	-9.170862	-1.774452	-1.917996
44	C	-10.000193	-2.575124	-1.132245
45	C	-9.521657	-3.086515	0.073154
46	C	-8.223705	-2.806541	0.489436
47	C	8.223692	-2.806586	-0.489597
48	C	9.521668	-3.086563	-0.073392
49	C	10.000286	-2.575149	1.131965
50	C	9.171013	-1.774451	1.917751
51	C	7.873585	-1.492890	1.505464
52	H	-6.033521	-4.442563	1.048777
53	H	-4.107964	-5.442016	2.251412
54	H	-2.091987	-4.025995	2.735178
55	H	-2.021209	-1.692495	1.907490
56	H	-5.759992	0.396390	-0.569437
57	H	-1.314215	-0.850461	0.108924
58	H	-2.844513	3.905860	0.309954
59	H	-4.469621	2.078265	0.505072
60	H	1.314255	-0.850460	-0.108888
61	H	4.469644	2.078281	-0.505069
62	H	2.844529	3.905871	-0.309958
63	H	5.760079	0.396393	0.569367
64	H	2.021065	-1.692524	-1.907162
65	H	2.091735	-4.026060	-2.734776
66	H	4.107746	-5.442084	-2.251158
67	H	6.033427	-4.442615	-1.048735
68	H	0.149834	7.550065	-2.137552
69	H	-0.000001	8.788378	-0.000020
70	H	-0.149831	7.550091	2.137527
71	H	-0.129213	5.580843	3.375074
72	H	0.650865	4.165658	2.656024
73	H	-1.100694	4.308238	2.630684
74	H	-0.650848	4.165622	-2.656007
75	H	1.100711	4.308210	-2.630668
76	H	0.129225	5.580801	-3.375075
77	H	-7.230738	-0.887387	-2.134025
78	H	-9.533659	-1.373889	-2.857580
79	H	-11.010946	-2.795388	-1.455196
80	H	-10.163729	-3.696960	0.697634
81	H	-7.883743	-3.179110	1.447735
82	H	7.883667	-3.179173	-1.447866
83	H	10.163695	-3.697027	-0.697900
84	H	11.011058	-2.795414	1.454856
85	H	9.533874	-1.373870	2.857302
86	H	7.230911	-0.887363	2.133884

*^{7DMA}**FluIndz** (S_0) Geometry and Frequency Data*
Total Energy: -2807.0022822 Hartrees
The optimized structure did not have any imaginary frequencies (n_i = 0).
XYZ Coordinates (in Å):

	Atom	x	y	z
1	C	-1.117916	5.168801	1.283932
2	C	0.119710	5.799001	1.144908
3	C	0.872472	5.045053	0.211036
4	N	0.097080	3.953458	-0.188310
5	C	-1.141215	3.999863	0.467761
6	C	-2.161937	3.001844	0.326012
7	C	2.147621	5.225111	-0.329249
8	C	2.653632	4.360002	-1.296161
9	C	1.785167	3.308961	-1.731020
10	C	0.544168	3.136329	-1.198596
11	C	3.991709	4.524398	-1.873490
12	C	0.631496	7.010214	1.861007
13	C	-2.179401	5.593286	2.227964
14	C	-1.833886	1.617184	0.156585
15	C	-2.833412	0.687017	0.057079
16	C	-4.213367	1.071844	0.104288
17	C	-4.543026	2.427515	0.280525
18	C	-3.535010	3.364397	0.396131
19	C	-2.805860	-0.781859	-0.079824
20	C	-4.170061	-1.218579	-0.127769
21	C	-5.027316	-0.088882	-0.012107
22	C	-1.772005	-1.673934	-0.177900
23	C	-2.047076	-3.070040	-0.344413
24	C	-3.405173	-3.484481	-0.415052
25	C	-4.448082	-2.585939	-0.302414
26	C	-0.987946	-4.028223	-0.481397
27	C	-6.509976	-0.116135	-0.012360
28	N	0.245458	-3.933194	0.179496
29	C	1.065304	-4.992935	-0.218454
30	C	0.343301	-5.779668	-1.149243
31	C	-0.916518	-5.196140	-1.295507
32	C	0.653822	-3.104615	1.196599
33	C	1.894039	-3.236258	1.742674
34	C	2.805283	-4.250613	1.310638
35	C	2.340242	-5.129539	0.335061
36	C	-1.956469	-5.660660	-2.244410
37	C	0.902500	-6.973524	-1.859084
38	C	4.145541	-4.369814	1.894860
39	C	-7.202077	-0.440525	1.173767
40	C	-8.599328	-0.456334	1.152643

41	C	-9.300899	-0.165182	-0.012385
42	C	-8.609989	0.150231	-1.177446
43	C	-7.213030	0.183440	-1.198541
44	C	-2.567087	4.767495	3.292691
45	C	-3.545896	5.181883	4.191643
46	C	-4.154285	6.428035	4.042287
47	C	-3.773850	7.259165	2.990448
48	C	-2.792090	6.846271	2.091402
49	C	4.643478	5.772807	-1.899503
50	C	5.905657	5.933803	-2.438802
51	C	6.614419	4.835059	-2.989721
52	C	5.962000	3.576378	-2.964961
53	C	4.694459	3.438272	-2.429134
54	C	-2.375593	-4.847585	-3.307090
55	C	-3.333504	-5.299130	-4.210621
56	C	-3.889431	-6.570347	-4.068024
57	C	-3.477395	-7.388964	-3.018278
58	C	-2.516424	-6.938828	-2.114711
59	C	5.204803	-4.980591	1.195798
60	C	6.471356	-5.102485	1.735649
61	C	6.763916	-4.619234	3.037071
62	C	5.700041	-4.004168	3.744474
63	C	4.441923	-3.884217	3.182797
64	N	7.866635	4.984062	-3.521572
65	C	8.507766	6.292816	-3.541853
66	C	8.556637	3.843388	-4.110189
67	N	8.013495	-4.739172	3.582410
68	C	9.087184	-5.377304	2.831475
69	C	8.287887	-4.219107	4.915840
70	C	-6.463294	-0.752359	2.454764
71	C	-6.485792	0.521066	-2.479612
72	H	2.733880	6.057411	0.036030
73	H	2.079908	2.651110	-2.536306
74	H	-0.140579	2.387914	-1.563212
75	H	-0.034691	7.292915	2.675971
76	H	0.715938	7.870354	1.187930
77	H	1.625023	6.831026	2.282507
78	H	-0.798094	1.302979	0.172771
79	H	-5.580471	2.740054	0.314765
80	H	-3.793307	4.407753	0.509453
81	H	-0.748852	-1.321027	-0.194973
82	H	-3.623785	-4.537135	-0.526387
83	H	-5.472940	-2.937486	-0.337245
84	H	-0.051190	-2.365741	1.540966
85	H	2.176576	-2.534373	2.514248
86	H	2.939390	-5.970636	0.013345

87	H	1.899372	-6.765967	-2.258724
88	H	0.261307	-7.270985	-2.688817
89	H	0.996057	-7.834035	-1.187591
90	H	-9.137903	-0.698234	2.062543
91	H	-10.384992	-0.184153	-0.012372
92	H	-9.156679	0.372865	-2.087434
93	H	-2.093688	3.800509	3.417987
94	H	-3.831467	4.532588	5.011551
95	H	-4.917262	6.749117	4.742116
96	H	-4.241232	8.229531	2.867067
97	H	-2.505867	7.493128	1.269811
98	H	4.139015	6.652546	-1.517325
99	H	6.342934	6.922122	-2.446144
100	H	6.454693	2.699786	-3.361119
101	H	4.254315	2.448061	-2.416536
102	H	-1.942878	-3.861084	-3.427263
103	H	-3.643800	-4.659154	-5.028864
104	H	-4.636237	-6.920395	-4.771400
105	H	-3.903960	-8.378583	-2.900023
106	H	-2.205797	-7.576336	-1.294718
107	H	5.044655	-5.344577	0.187505
108	H	7.245163	-5.566306	1.140463
109	H	5.858097	-3.625679	4.744382
110	H	3.662892	-3.426849	3.781271
111	H	8.627171	6.698773	-2.531521
112	H	7.937957	7.016326	-4.136248
113	H	9.496544	6.196739	-3.984409
114	H	8.723216	3.048396	-3.374919
115	H	9.526608	4.167514	-4.480058
116	H	7.996046	3.419711	-4.951244
117	H	9.305669	-4.841686	1.900620
118	H	8.843120	-6.415271	2.579778
119	H	9.990046	-5.381210	3.437746
120	H	8.118697	-3.137833	4.972093
121	H	9.329167	-4.410981	5.164037
122	H	7.663668	-4.702750	5.675453
123	H	-5.777487	0.054315	2.728654
124	H	-5.865334	-1.663657	2.363419
125	H	-7.164558	-0.893783	3.278399
126	H	-7.191061	0.629446	-3.304835
127	H	-5.765982	-0.256866	-2.749168
128	H	-5.927179	1.457333	-2.391118

*7DMA**FluIndz** (T_1) Geometry and Frequency Data*
Total Energy: -2806.992636 Hartrees
The optimized structure did not have any imaginary frequencies ($n_i = 0$).
XYZ Coordinates (in Å):

	Atom	x	y	z
1	C	-1.089766	4.975705	1.752223
2	C	0.098247	5.697711	1.584228
3	C	0.793755	5.111972	0.495565
4	N	0.030124	4.035632	0.030115
5	C	-1.133515	3.928838	0.793019
6	C	-2.137354	2.905619	0.580878
7	C	1.998851	5.423584	-0.128038
8	C	2.452861	4.701958	-1.235814
9	C	1.598800	3.655182	-1.711172
10	C	0.423039	3.353213	-1.096330
11	C	3.720377	5.006784	-1.896805
12	C	0.611609	6.842848	2.398527
13	C	-2.088764	5.210967	2.820355
14	C	-1.782094	1.562372	0.301795
15	C	-2.767725	0.613057	0.119899
16	C	-4.157217	0.967629	0.199492
17	C	-4.508911	2.300224	0.488914
18	C	-3.515310	3.240683	0.680994
19	C	-2.709150	-0.821843	-0.144067
20	C	-4.065267	-1.287157	-0.230986
21	C	-4.958381	-0.193903	-0.018586
22	C	-1.649406	-1.688960	-0.319139
23	C	-1.893788	-3.057148	-0.596614
24	C	-3.239809	-3.502089	-0.703837
25	C	-4.306869	-2.644152	-0.520085
26	C	-0.809576	-3.996521	-0.799314
27	C	-6.440558	-0.253341	-0.024460
28	N	0.356508	-4.004089	-0.032048
29	C	1.208902	-5.014135	-0.492233
30	C	0.564508	-5.664520	-1.575686
31	C	-0.676951	-5.041489	-1.752500
32	C	0.684964	-3.293896	1.097690
33	C	1.874544	-3.503943	1.724613
34	C	2.815881	-4.474388	1.253550
35	C	2.430315	-5.227534	0.140195
36	C	-1.648707	-5.362588	-2.823412
37	C	1.172639	-6.768924	-2.380921
38	C	4.099071	-4.682349	1.922213
39	C	-7.129294	-0.771518	1.094174
40	C	-8.526194	-0.818810	1.069575

41	C	-9.237785	-0.366022	-0.036398
42	C	-8.555540	0.143041	-1.136395
43	C	-7.159213	0.208297	-1.149095
44	C	-2.413697	4.201013	3.737585
45	C	-3.335832	4.434815	4.753702
46	C	-3.950791	5.681121	4.871152
47	C	-3.634567	6.693345	3.967068
48	C	-2.709649	6.461074	2.950944
49	C	4.337343	6.270808	-1.782343
50	C	5.532485	6.566103	-2.405231
51	C	6.209493	5.597092	-3.192679
52	C	5.595391	4.322484	-3.308143
53	C	4.393775	4.050137	-2.684795
54	C	-2.048533	-4.388190	-3.749478
55	C	-2.943887	-4.702120	-4.767976
56	C	-3.456686	-5.994381	-4.879050
57	C	-3.065424	-6.971769	-3.966036
58	C	-2.167217	-6.659144	-2.947528
59	C	5.189839	-5.289286	1.263656
60	C	6.405107	-5.493015	1.884434
61	C	6.614724	-5.103165	3.234012
62	C	5.520772	-4.492823	3.901809
63	C	4.315351	-4.290130	3.259803
64	N	7.393905	5.879505	-3.810396
65	C	8.007363	7.195294	-3.666745
66	C	8.053559	4.875666	-4.638122
67	N	7.811986	-5.304668	3.858564
68	C	8.912084	-5.955485	3.155401
69	C	8.006487	-4.874971	5.239030
70	C	-6.384294	-1.257330	2.316244
71	C	-6.445260	0.752930	-2.364852
72	H	2.579795	6.237850	0.282942
73	H	1.849819	3.104455	-2.606211
74	H	-0.253123	2.600009	-1.468816
75	H	0.072540	6.924024	3.342550
76	H	0.497772	7.795932	1.870164
77	H	1.675054	6.722657	2.622886
78	H	-0.738063	1.272422	0.281798
79	H	-5.551231	2.590538	0.556598
80	H	-3.788269	4.267100	0.887581
81	H	-0.632114	-1.315898	-0.294624
82	H	-3.428174	-4.547488	-0.909575
83	H	-5.322003	-3.017510	-0.593495
84	H	-0.041620	-2.580387	1.452304
85	H	2.094754	-2.895412	2.589655
86	H	3.054486	-6.028068	-0.232413

87	H	2.221691	-6.560397	-2.608345
88	H	0.641489	-6.904720	-3.323118
89	H	1.141596	-7.723207	-1.843389
90	H	-9.057255	-1.211132	1.930482
91	H	-10.321363	-0.409695	-0.041030
92	H	-9.109109	0.491720	-2.001876
93	H	-1.935107	3.232101	3.656513
94	H	-3.572552	3.643749	5.456216
95	H	-4.670054	5.861590	5.661783
96	H	-4.109293	7.664508	4.049451
97	H	-2.477083	7.249147	2.243791
98	H	3.853735	7.058691	-1.217576
99	H	5.942242	7.559910	-2.294614
100	H	6.069435	3.539752	-3.882996
101	H	3.984857	3.052474	-2.789282
102	H	-1.649325	-3.383561	-3.673340
103	H	-3.239405	-3.937583	-5.477547
104	H	-4.155199	-6.237117	-5.671608
105	H	-3.460689	-7.978300	-4.043316
106	H	-1.876252	-7.420982	-2.233322
107	H	5.097196	-5.582933	0.224909
108	H	7.205977	-5.946921	1.318404
109	H	5.615336	-4.186449	4.933815
110	H	3.510117	-3.842823	3.829436
111	H	8.212044	7.429808	-2.617109
112	H	7.370275	7.985738	-4.078361
113	H	8.951759	7.206113	-4.205340
114	H	8.330399	3.990692	-4.055074
115	H	8.961599	5.303009	-5.056403
116	H	7.414276	4.555446	-5.467605
117	H	9.230251	-5.377804	2.280741
118	H	8.636324	-6.961361	2.822040
119	H	9.760684	-6.043745	3.829347
120	H	7.851420	-3.796615	5.349013
121	H	9.026000	-5.102155	5.540780
122	H	7.325047	-5.392464	5.922971
123	H	-5.686965	-0.500644	2.685954
124	H	-5.795438	-2.153653	2.100118
125	H	-7.080600	-1.500928	3.120517
126	H	-7.151985	0.940810	-3.174975
127	H	-5.686586	0.054674	-2.728701
128	H	-5.931720	1.693203	-2.143589

***[1,7DBA]FluIndz** (S_0) Geometry and Frequency Data*
Total Energy: -3458.5727606 Hartrees
The optimized structure did not have any imaginary frequencies (n_i = 0).
XYZ Coordinates (in Å):

	Atom	x	y	z
1	C	-5.907135	0.084106	-0.105716
2	C	-4.957087	-0.621487	-0.897239
3	N	-3.833863	0.194635	-1.047829
4	C	-4.039745	1.403125	-0.367291
5	C	-5.336680	1.329299	0.204348
6	C	-7.200787	-0.462959	0.331855
7	C	-5.963266	2.374070	1.049932
8	C	-3.058791	2.453224	-0.298860
9	C	-4.958527	-1.896889	-1.468345
10	C	-3.895566	-2.346041	-2.247816
11	C	-2.824405	-1.422527	-2.464298
12	C	-2.814825	-0.191185	-1.885325
13	C	-1.663954	2.157468	-0.180838
14	C	-0.754837	3.178656	-0.087849
15	C	-1.173741	4.549252	-0.124170
16	C	-2.544484	4.846232	-0.226076
17	C	-3.461517	3.815567	-0.302685
18	C	0.709514	3.185314	0.077304
19	C	1.116151	4.559467	0.111925
20	C	-0.032699	5.391433	-0.006691
21	C	1.627903	2.172265	0.170793
22	C	3.019770	2.480507	0.288464
23	C	3.410234	3.846319	0.291663
24	C	2.484084	4.868824	0.213712
25	C	-3.873398	-3.684862	-2.846373
26	C	4.010344	1.439210	0.357403
27	C	-0.039451	6.873829	-0.007804
28	N	3.817317	0.229715	1.040658
29	C	4.947910	-0.576426	0.887532
30	C	5.891641	0.139205	0.097558
31	C	5.306892	1.377058	-0.215865
32	C	2.807225	-0.162541	1.885652
33	C	2.835376	-1.387929	2.477299
34	C	3.915113	-2.300363	2.261936
35	C	4.968202	-1.845198	1.472051
36	C	3.917869	-3.636698	2.867974
37	C	5.920451	2.425336	-1.066576
38	C	7.190598	-0.394656	-0.341354
39	C	-5.364440	2.787672	2.247768
40	C	-5.965490	3.760342	3.042883

41	C	-7.173793	4.336634	2.652967
42	C	-7.779102	3.930763	1.464398
43	C	-7.180496	2.955281	0.670539
44	C	-7.622496	-0.386532	1.669342
45	C	-8.835631	-0.908910	2.088478
46	C	-9.719347	-1.537075	1.177288
47	C	-9.292672	-1.621098	-0.169487
48	C	-8.072472	-1.096134	-0.569486
49	C	-5.055198	-4.407275	-3.102574
50	C	-5.039955	-5.670542	-3.662896
51	C	-3.819134	-6.308081	-4.003703
52	C	-2.627050	-5.584999	-3.746248
53	C	-2.664409	-4.318467	-3.191943
54	C	-0.154082	7.571097	-1.229645
55	C	-0.155495	8.968428	-1.208540
56	C	-0.052770	9.666437	-0.010040
57	C	0.056692	8.971354	1.189570
58	C	0.068663	7.574115	1.212902
59	C	7.133582	3.019029	-0.693399
60	C	7.720035	3.997832	-1.492214
61	C	7.106561	4.394646	-2.679666
62	C	5.902279	3.805943	-3.063428
63	C	5.313362	2.829951	-2.263324
64	C	7.607164	-0.319389	-1.680519
65	C	8.825116	-0.829211	-2.101334
66	C	9.719016	-1.442880	-1.190240
67	C	9.297641	-1.525823	0.158229
68	C	8.072730	-1.013238	0.560031
69	C	4.654420	-4.699656	2.309840
70	C	4.667820	-5.962548	2.871518
71	C	3.938637	-6.247425	4.054543
72	C	3.195876	-5.180178	4.619766
73	C	3.188478	-3.925761	4.036995
74	N	-10.938208	-2.034582	1.580677
75	C	-11.285047	-2.054413	2.994633
76	C	-11.751127	-2.808471	0.653310
77	N	-3.793861	-7.560104	-4.556208
78	C	-5.035964	-8.280321	-4.806076
79	C	-2.525984	-8.175050	-4.927798
80	N	3.950337	-7.493413	4.621435
81	C	4.719211	-8.571936	4.013734
82	C	3.177353	-7.760724	5.827470
83	N	10.942381	-1.928075	-1.595294
84	C	11.285058	-1.950028	-3.010179
85	C	11.766395	-2.689358	-0.667256
86	C	0.176855	6.844053	2.531839

87	C	-0.255137	6.838000	-2.547478
88	H	-5.810514	-2.531407	-1.270196
89	H	-2.010508	-1.669491	-3.131043
90	H	-2.038891	0.532044	-2.077557
91	H	-1.336776	1.128371	-0.102278
92	H	-2.882369	5.875883	-0.255958
93	H	-4.512262	4.048511	-0.402776
94	H	1.309791	1.140245	0.092923
95	H	4.458867	4.088677	0.391737
96	H	2.812821	5.901462	0.242427
97	H	2.010285	0.542590	2.057029
98	H	1.998472	-1.659066	3.104932
99	H	5.849446	-2.451598	1.320375
100	H	-4.427640	2.339627	2.558786
101	H	-5.490761	4.066405	3.968343
102	H	-7.640751	5.094703	3.271354
103	H	-8.718726	4.373587	1.153850
104	H	-7.654723	2.643063	-0.252575
105	H	-6.977319	0.078491	2.405145
106	H	-9.095136	-0.833040	3.135118
107	H	-9.921340	-2.089770	-0.913532
108	H	-7.804568	-1.158901	-1.618317
109	H	-6.018419	-3.959956	-2.887013
110	H	-5.982758	-6.164442	-3.850891
111	H	-1.665102	-6.022012	-3.973711
112	H	-1.720565	-3.823651	-2.995350
113	H	-0.235580	9.510109	-2.144853
114	H	-0.057939	10.750697	-0.010904
115	H	0.131666	9.515278	2.125006
116	H	7.614142	2.713875	0.228794
117	H	8.656594	4.450329	-1.186394
118	H	7.564086	5.155305	-3.301913
119	H	5.421262	4.104923	-3.987957
120	H	4.379653	2.372278	-2.569582
121	H	6.954240	0.134784	-2.416296
122	H	9.080448	-0.754790	-3.149116
123	H	9.933991	-1.983852	0.902369
124	H	7.809394	-1.074824	1.610106
125	H	5.209693	-4.546044	1.391825
126	H	5.239323	-6.739590	2.383905
127	H	2.626898	-5.332483	5.525991
128	H	2.621204	-3.144227	4.528465
129	H	-11.262376	-1.047601	3.422165
130	H	-10.610375	-2.690388	3.583537
131	H	-12.297517	-2.437595	3.105611
132	H	-12.003821	-2.223983	-0.236586

133	H	-12.683133	-3.083154	1.143227
134	H	-11.250742	-3.729539	0.325270
135	H	-5.677275	-7.750952	-5.520211
136	H	-5.605653	-8.433187	-3.883059
137	H	-4.802151	-9.257443	-5.222412
138	H	-1.987257	-7.575519	-5.670234
139	H	-2.719525	-9.153676	-5.360863
140	H	-1.872091	-8.311975	-4.059209
141	H	5.786681	-8.330031	3.965356
142	H	4.372947	-8.795693	2.998212
143	H	4.604837	-9.471320	4.614427
144	H	3.497696	-7.126961	6.661810
145	H	3.321972	-8.798426	6.119078
146	H	2.105370	-7.598604	5.667142
147	H	11.247884	-0.945598	-3.442240
148	H	10.616954	-2.597452	-3.594174
149	H	12.302149	-2.320369	-3.122621
150	H	12.015877	-2.098366	0.219236
151	H	12.699584	-2.956550	-1.159062
152	H	11.276682	-3.614087	-0.333441
153	H	0.144292	7.548156	3.364588
154	H	1.111596	6.280946	2.605880
155	H	-0.639030	6.127543	2.661531
156	H	-1.185899	6.268386	-2.622076
157	H	0.565826	6.126884	-2.674639
158	H	-0.226077	7.540880	-3.381396

***1,7DBA*FluIndz** *(T_1) Geometry and Frequency Data*
Total Energy: -3458.5669722 Hartrees
The optimized structure did not have any imaginary frequencies (n_i = 0).
XYZ Coordinates (in Å):

	Atom	x	y	z
1	C	-5.870527	0.081632	0.128667
2	C	-5.093127	-0.488709	-0.934057
3	N	-3.987471	0.337429	-1.131104
4	C	-4.028152	1.404672	-0.224252
5	C	-5.177985	1.253560	0.551619
6	C	-7.087408	-0.489088	0.670644
7	C	-5.598068	2.196528	1.616371
8	C	-3.003773	2.452590	-0.200590
9	C	-5.202937	-1.655861	-1.680674
10	C	-4.269633	-1.979683	-2.677036
11	C	-3.211411	-1.044738	-2.892231
12	C	-3.087542	0.076736	-2.128893
13	C	-1.633170	2.123141	-0.117015
14	C	-0.688246	3.133309	-0.075759
15	C	-1.085845	4.506933	-0.122163
16	C	-2.447538	4.830497	-0.194521
17	C	-3.388858	3.809885	-0.230513
18	C	0.770339	3.111517	0.035233
19	C	1.212879	4.477152	0.042405
20	C	0.086498	5.341827	-0.052371
21	C	1.675949	2.072443	0.111623
22	C	3.064579	2.345238	0.193780
23	C	3.492067	3.699141	0.182692
24	C	2.594102	4.747620	0.110787
25	C	-4.373815	-3.207808	-3.458003
26	C	4.044499	1.271457	0.254279
27	C	0.106482	6.824019	-0.074114
28	N	3.888033	0.138640	1.053010
29	C	4.999888	-0.702242	0.895866
30	C	5.884920	-0.082944	-0.014709
31	C	5.283533	1.136670	-0.404765
32	C	2.916482	-0.169664	1.978134
33	C	2.969481	-1.336996	2.673466
34	C	4.031870	-2.279398	2.480171
35	C	5.036315	-1.919574	1.595117
36	C	4.053187	-3.560222	3.207249
37	C	5.843172	2.103024	-1.378942
38	C	7.148585	-0.671290	-0.492544
39	C	-4.769896	2.449360	2.717709
40	C	-5.165536	3.340612	3.712570

41	C	-6.393161	3.994393	3.620275
42	C	-7.223864	3.751228	2.526905
43	C	-6.830846	2.858383	1.533386
44	C	-7.383333	-0.432469	2.052315
45	C	-8.524472	-0.996224	2.579781
46	C	-9.476585	-1.647432	1.748461
47	C	-9.196119	-1.686725	0.356028
48	C	-8.039872	-1.132781	-0.152607
49	C	-5.588591	-3.917916	-3.576920
50	C	-5.693437	-5.079198	-4.312681
51	C	-4.567779	-5.626924	-4.985212
52	C	-3.342487	-4.918056	-4.867146
53	C	-3.260956	-3.751998	-4.134752
54	C	-0.074760	7.512319	-1.293845
55	C	-0.052056	8.909884	-1.294174
56	C	0.142418	9.622332	-0.115511
57	C	0.318827	8.940189	1.083658
58	C	0.305434	7.542970	1.124930
59	C	7.115471	2.657528	-1.180140
60	C	7.648914	3.562398	-2.094652
61	C	6.922404	3.926032	-3.227921
62	C	5.658083	3.377655	-3.439345
63	C	5.123296	2.475095	-2.523134
64	C	7.461932	-0.744072	-1.858903
65	C	8.646766	-1.303247	-2.314469
66	C	9.599181	-1.839663	-1.415912
67	C	9.293795	-1.750866	-0.038167
68	C	8.102634	-1.186854	0.399063
69	C	4.682292	-4.700499	2.676383
70	C	4.716194	-5.907890	3.353309
71	C	4.121299	-6.049782	4.631260
72	C	3.475316	-4.907411	5.162843
73	C	3.447496	-3.709509	4.466997
74	N	-10.609057	-2.204192	2.263877
75	C	-10.889609	-2.124217	3.694665
76	C	-11.569097	-2.873443	1.391101
77	N	-4.659754	-6.777944	-5.709967
78	C	-5.927611	-7.495082	-5.802865
79	C	-3.495956	-7.300925	-6.419024
80	N	4.171003	-7.237793	5.321841
81	C	4.732354	-8.425217	4.692916
82	C	3.430765	-7.389778	6.566579
83	N	10.766287	-2.429367	-1.863741
84	C	11.142906	-2.306638	-3.264900
85	C	11.808196	-2.778171	-0.908466
86	C	0.486955	6.829907	2.445214

87	C	-0.272265	6.764615	-2.592410
88	H	-6.014143	-2.329663	-1.451040
89	H	-2.492952	-1.193201	-3.684895
90	H	-2.302551	0.803049	-2.273553
91	H	-1.329406	1.084013	-0.052149
92	H	-2.768092	5.865735	-0.223032
93	H	-4.441243	4.055852	-0.293891
94	H	1.331894	1.045724	0.064774
95	H	4.550954	3.910293	0.253681
96	H	2.952635	5.770913	0.117289
97	H	2.129426	0.554953	2.114438
98	H	2.166726	-1.547102	3.366506
99	H	5.896519	-2.557130	1.444507
100	H	-3.815697	1.941416	2.796132
101	H	-4.514267	3.523259	4.559771
102	H	-6.699955	4.689192	4.393668
103	H	-8.178175	4.259151	2.445597
104	H	-7.479006	2.676140	0.683978
105	H	-6.680851	0.038210	2.727202
106	H	-8.681923	-0.943065	3.647493
107	H	-9.898975	-2.139068	-0.328866
108	H	-7.893241	-1.152486	-1.225250
109	H	-6.487108	-3.535996	-3.107812
110	H	-6.656637	-5.564145	-4.380714
111	H	-2.448794	-5.292715	-5.345130
112	H	-2.294300	-3.269244	-4.061112
113	H	-0.183794	9.440588	-2.231206
114	H	0.156589	10.706571	-0.131517
115	H	0.465240	9.494891	2.004487
116	H	7.684365	2.379919	-0.300534
117	H	8.632562	3.984352	-1.921059
118	H	7.338285	4.629515	-3.940302
119	H	5.087378	3.650950	-4.319937
120	H	4.142049	2.048596	-2.696612
121	H	6.758201	-0.357515	-2.586913
122	H	8.828541	-1.326185	-3.379907
123	H	9.993717	-2.113422	0.701601
124	H	7.925120	-1.121325	1.466995
125	H	5.132957	-4.654553	1.691352
126	H	5.198311	-6.751546	2.879757
127	H	2.997757	-4.952277	6.131552
128	H	2.960456	-2.861887	4.935352
129	H	-10.969642	-1.085144	4.029597
130	H	-10.110371	-2.618843	4.283297
131	H	-11.834899	-2.620840	3.897906
132	H	-11.994558	-2.181867	0.656370

133	H	-12.381380	-3.268598	1.995664
134	H	-11.105902	-3.707283	0.854516
135	H	-6.698262	-6.890960	-6.293735
136	H	-6.295027	-7.786860	-4.813933
137	H	-5.780594	-8.398987	-6.388711
138	H	-3.113701	-6.579687	-7.148606
139	H	-3.783191	-8.202960	-6.953515
140	H	-2.685403	-7.556913	-5.728612
141	H	5.769971	-8.259553	4.387765
142	H	4.162412	-8.739086	3.808310
143	H	4.724742	-9.243204	5.410427
144	H	3.748474	-6.652517	7.310118
145	H	3.626458	-8.378426	6.976686
146	H	2.347082	-7.285062	6.423673
147	H	11.312324	-1.263938	-3.568764
148	H	10.374557	-2.729677	-3.917805
149	H	12.061371	-2.865880	-3.434483
150	H	12.216465	-1.901001	-0.386567
151	H	12.622464	-3.270905	-1.436964
152	H	11.433608	-3.476878	-0.155372
153	H	0.492240	7.543482	3.270925
154	H	1.428500	6.273923	2.476915
155	H	-0.314653	6.107944	2.623380
156	H	-1.217073	6.213227	-2.600821
157	H	0.524087	6.033593	-2.757081
158	H	-0.280519	7.455028	-3.437551

Nucleus-independent chemical shift (NICS) calculations for (anti-)aromaticity

In the NICS analysis reported here, the ZZ component of the magnetic shielding tensor has been determined at a series of points on a line that runs through the geometric center of each ring and is perpendicular to a mean ring plane taken to be the average of the 5 or 6 planes defined by the bond angles in each ring. The most relevant data for this analysis are typically reported at 1 Ångstrom (Å) above or below the ring, denoted here as $NICS_{ZZ}(+1)$ or $NICS_{ZZ}(-1)$. Negative $NICS_{ZZ}$ values signify aromatic character for the ring, whereas positive values are indicative of an antiaromatic ring. These sign conventions are consistent with those used for reporting NMR chemical shifts.

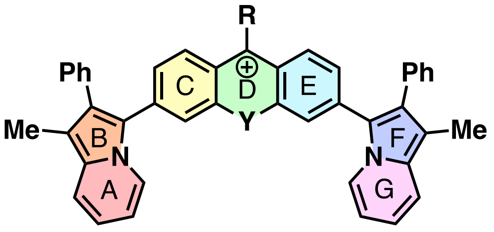

Y = O (**[tol]RosIndz**)
SiMe$_2$ (**SiRos1300**)
none (**[2Ph]FluIndz**)

Figure S13. Generic indolizine-xanthene structure with ring naming/color system used for NICS analysis.

Table S7. $NICS_{ZZ}$ (in ppm) at ±1 Å from the center of each ring of **[tol]RosIndz** for both the singlet ground (S_0) and first triplet (T_1) states. Values are rounded to the first decimal place. Note that the values are not symmetric above and below many of the rings because the two faces of these rings are not symmetry equivalent. For this reason, an average is given, which is the value reported in Figure 4.

State	Ring	$NICS_{ZZ}(+1)$	$NICS_{ZZ}(-1)$	$NICS_{ZZ}(avg)$
Singlet (S_0)	A	-13.5	-8.2	-10.9
	B	-15.0	-17.7	-16.4
	C	-16.2	-15.9	-16.1
	D	-4.6	-4.9	-4.8
	E	-15.7	-16.4	-16.0
	F	-18.8	-16.2	-16.2
	G	-9.0	-14.2	-11.6
Triplet (T_1)	A	-9.5	-5.2	-7.3
	B	-6.6	-9.2	-7.9
	C	-11.9	-12.1	-12.0
	D	+11.1	+10.8	+11.0
	E	-11.8	-11.9	-11.8
	F	-10.1	-7.5	-8.8
	G	-6.0	-10.4	-8.2

Table S8. $NICS_{ZZ}$ (in ppm) at ±1 Å from the center of each ring of **SiRos1300** for both the singlet ground (S_0) and first triplet (T_1) states. Values are rounded to the first decimal place. Note that the values are not symmetric above and below many of the rings because the two faces of these rings are not symmetry equivalent. For this reason, an average is given, which is the value reported in Figure 4.

State	Ring	$NICS_{ZZ}(+1)$	$NICS_{ZZ}(-1)$	$NICS_{ZZ}(avg)$
Singlet (S_0)	A	-13.0	-7.8	-10.4
	B	-13.8	-16.4	-15.1
	C	-12.4	-12.5	-12.4
	D	+9.1	+9.0	+9.1
	E	-12.5	-12.4	-12.4
	F	-16.4	-13.8	-15.1
	G	-7.8	-13.0	-10.4
Triplet (T_1)	A	-9.1	-4.7	-6.9
	B	-6.2	-8.7	-7.5
	C	-14.8	-14.8	-14.8
	D	+7.5	+7.4	+7.5
	E	-14.5	-14.6	-14.6
	F	-9.1	-6.5	-7.8
	G	-5.1	-9.6	-7.3

Table S9. $NICS_{ZZ}$ (in ppm) at ±1 Å from the center of each ring of **^{2Ph}FluIndz** for both the singlet ground (S_0) and first triplet (T_1) states. Values are rounded to the first decimal place. Note that the values are not symmetric above and below many of the rings because the two faces of these rings are not symmetry equivalent. For this reason, an average is given, which is the value reported in Figure 4.

State	Ring	$NICS_{ZZ}(+1)$	$NICS_{ZZ}(-1)$	$NICS_{ZZ}(avg)$
Singlet (S_0)	A	-9.5	-14.6	-12.0
	B	-17.1	-14.7	-15.9
	C	-0.1	+0.3	+0.1
	D	+25.6	+25.6	+25.6
	E	+0.3	-0.1	+0.1
	F	-14.7	-17.1	-15.9
	G	-14.6	-9.5	-12.0
Triplet (T_1)	A	-6.8	-11.3	-9.1
	B	-10.7	-8.1	-9.4
	C	-11.6	-11.7	-11.6
	D	-0.5	-0.5	-0.5
	E	-11.7	-11.6	-11.6
	F	-8.1	-10.7	-9.4
	G	-11.3	-6.8	-9.0

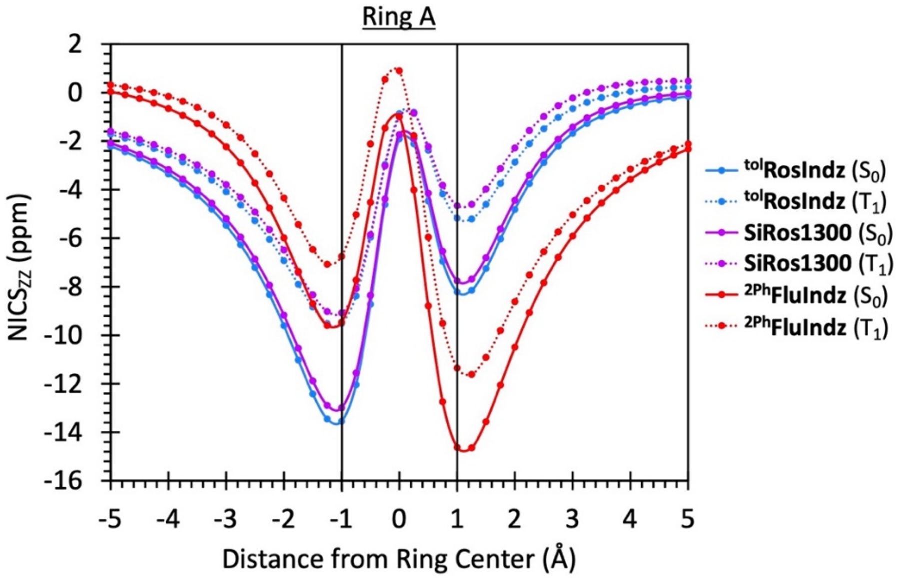

Figure S14. NICS$_{ZZ}$ (in ppm) versus distance from the center of ring A (in Å) of **^{tol}RosIndz**, **SiRos1300**, and **^{2Ph}FluIndz**. The S_0 (singlet ground state) calculations are shown as solid lines and the T_1 (first triplet state) calculations are shown as dashed lines. Vertical lines at +1 and -1 Å indicate the source of the values given in Figure 4 and the tables above. Note that the data curves are not symmetric about the y-axis because the two faces of the ring are not symmetry equivalent.

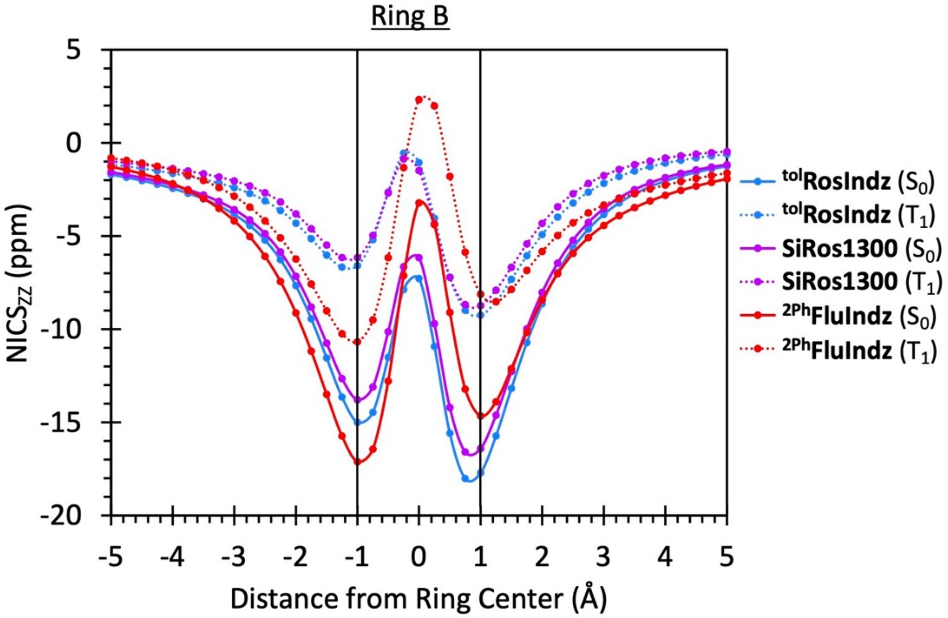

Figure S15. NICS$_{ZZ}$ (in ppm) versus distance from the center of ring B (in Å) of **^{tol}RosIndz**, **SiRos1300**, and **^{2Ph}FluIndz**. The S_0 (singlet ground state) calculations are shown as solid lines and the T_1 (first triplet state) calculations are shown as dashed lines. Vertical lines at +1 and -1 Å indicate the source of the values given in Figure 4 and the tables above. Note that the data curves are not symmetric about the y-axis because the two faces of the ring are not symmetry equivalent.

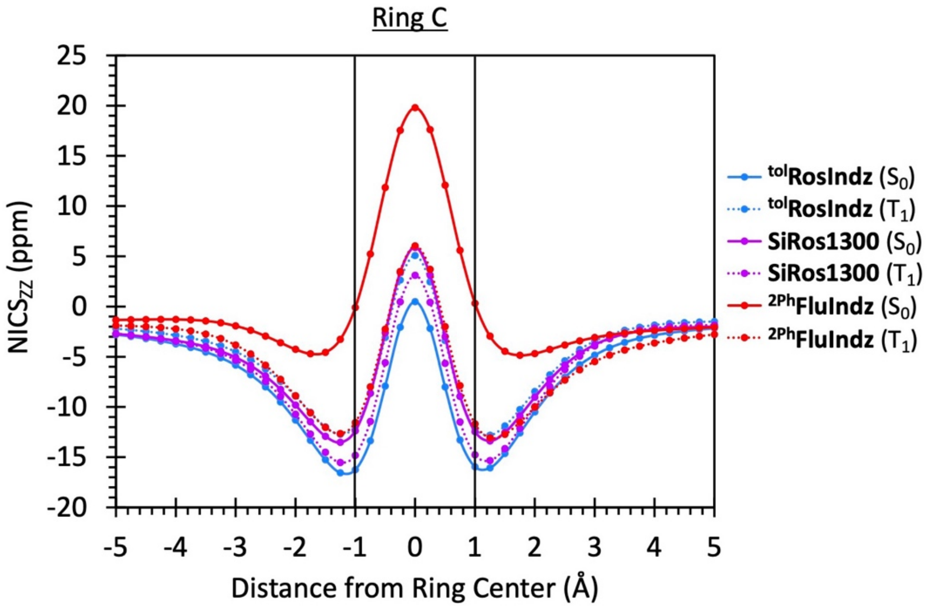

Figure S16. NICS$_{ZZ}$ (in ppm) versus distance from the center of ring C (in Å) of ^{tol}**RosIndz**, **SiRos1300**, and ^{2Ph}**FluIndz**. The S_0 (singlet ground state) calculations are shown as solid lines and the T_1 (first triplet state) calculations are shown as dashed lines. Vertical lines at +1 and -1 Å indicate the source of the values given in Figure 4 and the tables above.

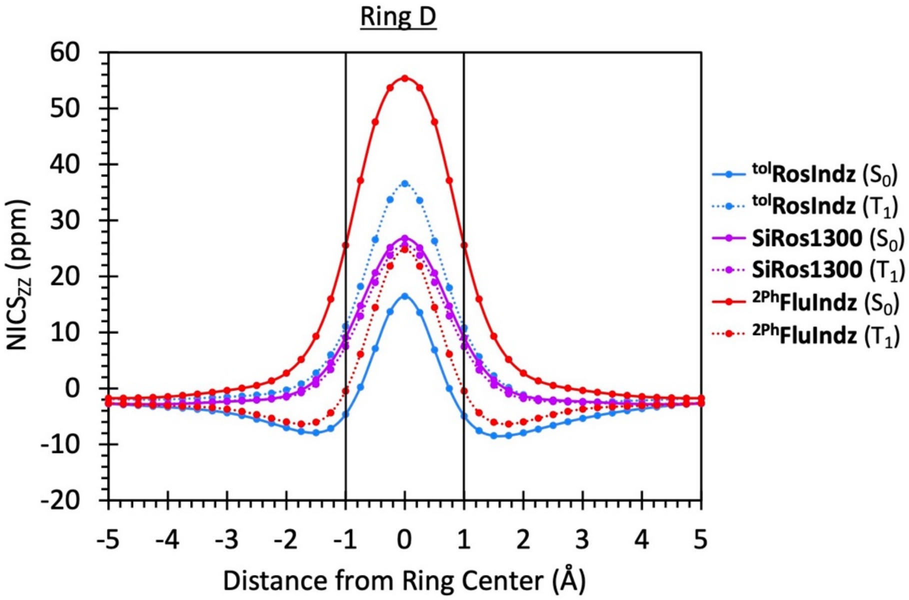

Figure S17. NICS$_{ZZ}$ (in ppm) versus distance from the center of ring D (in Å) of ^{tol}**RosIndz**, **SiRos1300**, and ^{2Ph}**FluIndz**. The S_0 (singlet ground state) calculations are shown as solid lines and the T_1 (first triplet state) calculations are shown as dashed lines. Vertical lines at +1 and -1 Å indicate the source of the values given in Figure 4 and the tables above.

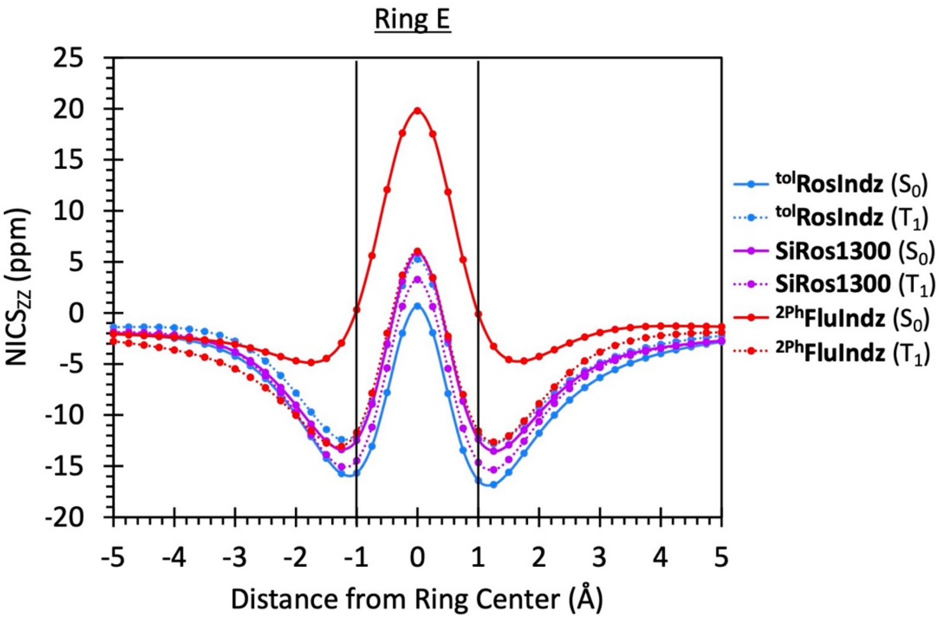

Figure S18. NICS$_{ZZ}$ (in ppm) versus distance from the center of ring E (in Å) of **^{tol}RosIndz**, **SiRos1300**, and **^{2Ph}FluIndz**. The S_0 (singlet ground state) calculations are shown as solid lines and the T_1 (first triplet state) calculations are shown as dashed lines. Vertical lines at +1 and -1 Å indicate the source of the values given in Figure 4 and the tables above.

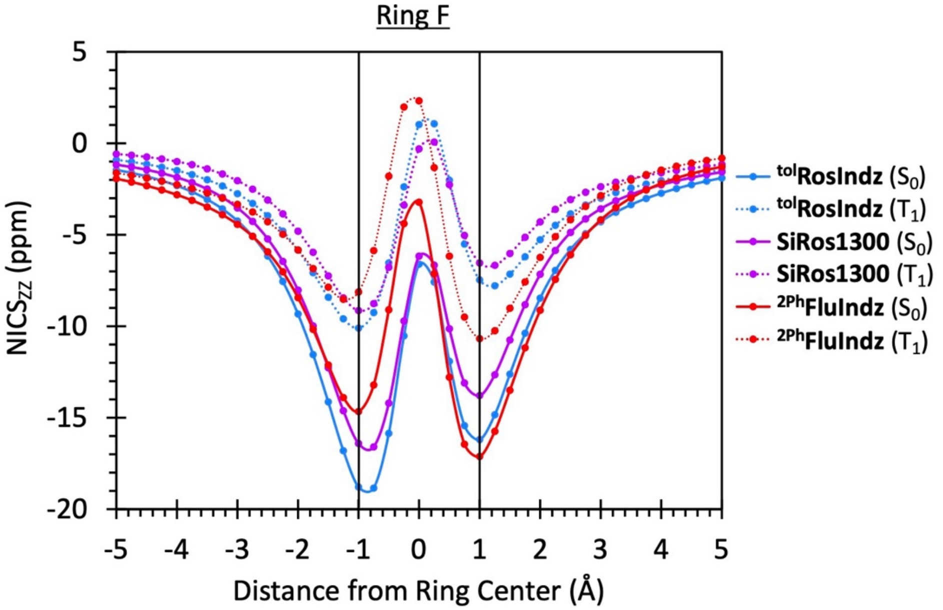

Figure S19. NICS$_{ZZ}$ (in ppm) versus distance from the center of ring F (in Å) of **^{tol}RosIndz**, **SiRos1300**, and **^{2Ph}FluIndz**. The S_0 (singlet ground state) calculations are shown as solid lines and the T_1 (first triplet state) calculations are shown as dashed lines. Vertical lines at +1 and -1 Å indicate the source of the values given in Figure 4 and the tables above. Note that the data curves are not symmetric about the y-axis because the two faces of the ring are not symmetry equivalent.

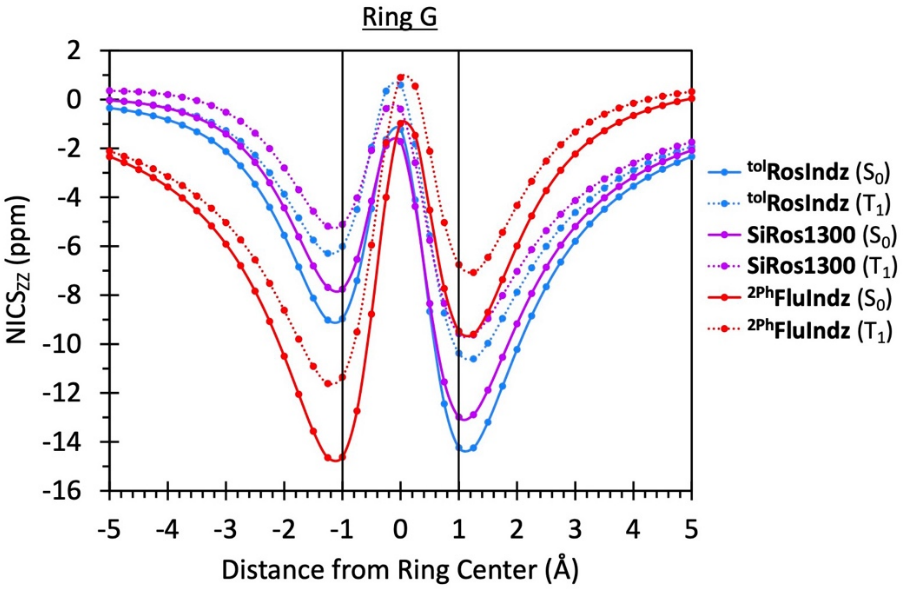

Figure S20. NICS$_{ZZ}$ (in ppm) versus distance from the center of ring G (in Å) of **^{tol}RosIndz**, **SiRos1300**, and **^{2Ph}FluIndz**. The S$_0$ (singlet ground state) calculations are shown as solid lines and the T$_1$ (first triplet state) calculations are shown as dashed lines. Vertical lines at +1 and -1 Å indicate the source of the values given in Figure 4 and the tables above. Note that the data curves are not symmetric about the y-axis because the two faces of the ring are not symmetry equivalent.

CRYSTALLOGRAPHIC DATA

The obtained **^{1Ph}FluIndz** crystals were thin, splintered shards. While diffraction with MoK(alpha) was observed to be weak, diffraction with CuK(alpha) provided sufficient, identifiable reflections at high enough resolution to allow for data collection. The structure was readily solved with the organic portion being well resolved. One PF_6^- anion is observed to sit on a crystallographic inversion, meaning only half of the anion is present in each asymmetric unit and that another half anionic charge must be located elsewhere. In the remaining difference density, another PF_6^- anion was observed, which must be present at half occupancy for the charges to match. The rest of the time this site is observed to be occupied by a disordered superposition of acetonitrile and diethyl ether. This unusual combination elucidated a deeper examination of reciprocal space to see if any cell doubling had been missed, however, no sensible doubling of the unit cell was discovered, so the partial anion/solvent overlap is not clearly resolved herein. This information is provided for full disclosure due to the uniqueness of the solved structure. However, it is emphasized that the chemically important part, the cation structure of **^{1Ph}FluIndz**, is in agreement with the predicted structure and makes good chemical sense.

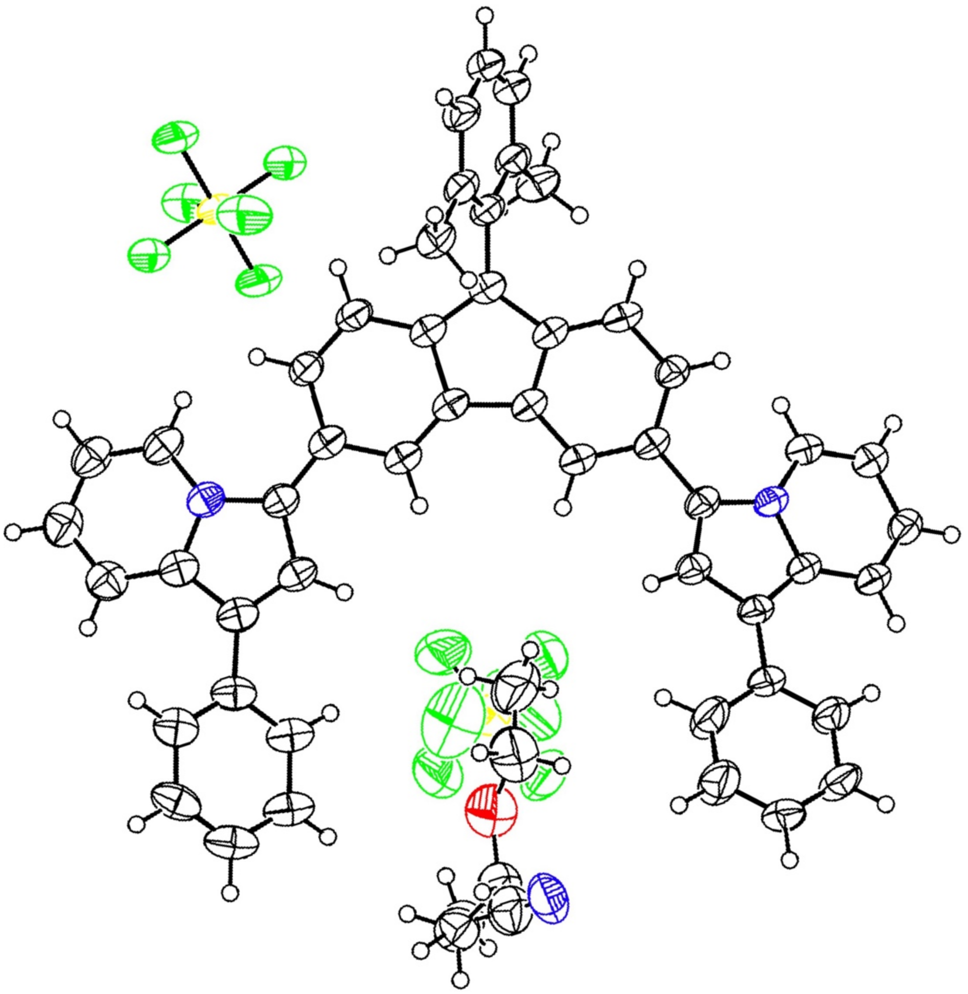

Figure S21. Thermal ellipsoid plot at 50% probability for a single [1Ph]**FluIndz** molecule with added hydrogens, anions, and solvent molecules.

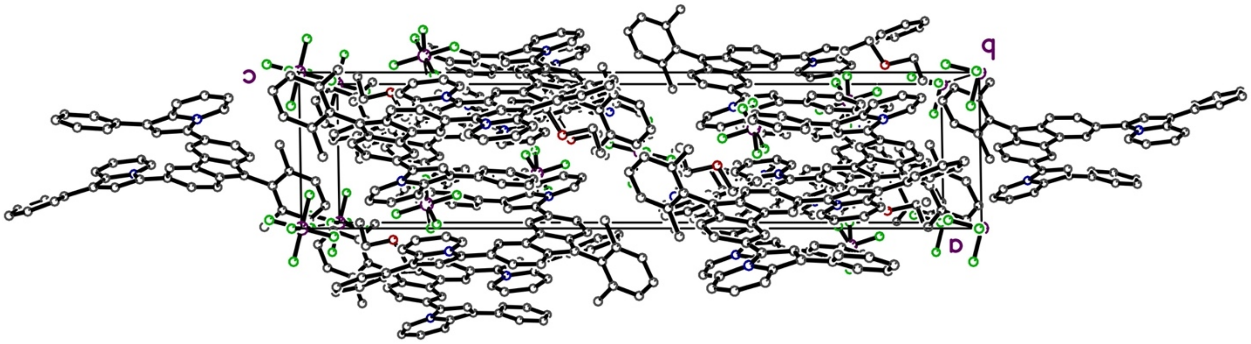

Figure S22. Perspective of the crystal packing structure for $^{\mathbf{1Ph}}$**FluIndz**.

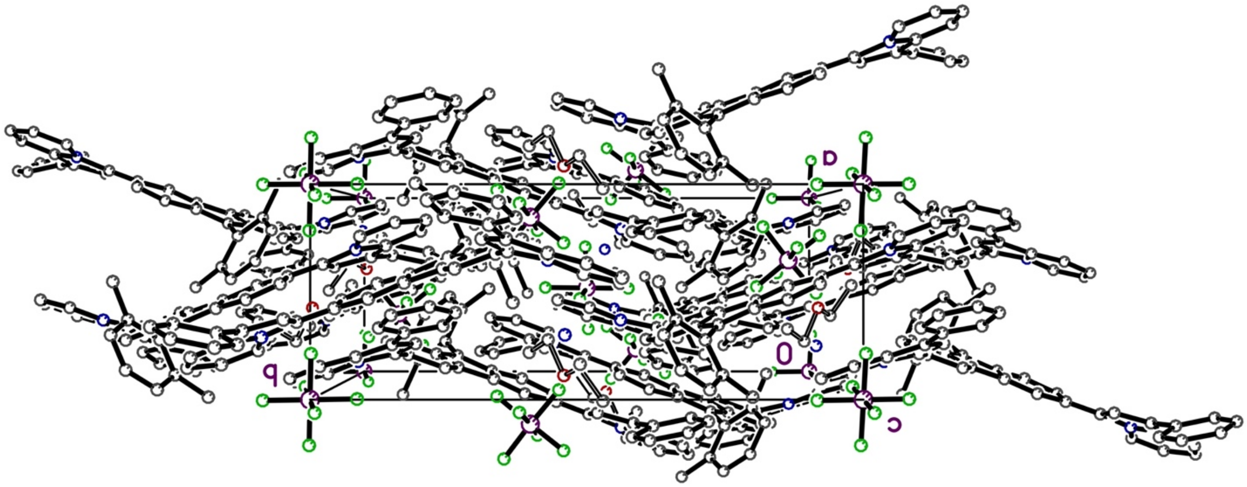

Figure S23. Perspective of the crystal packing structure for $^{\mathbf{1Ph}}$**FluIndz**.

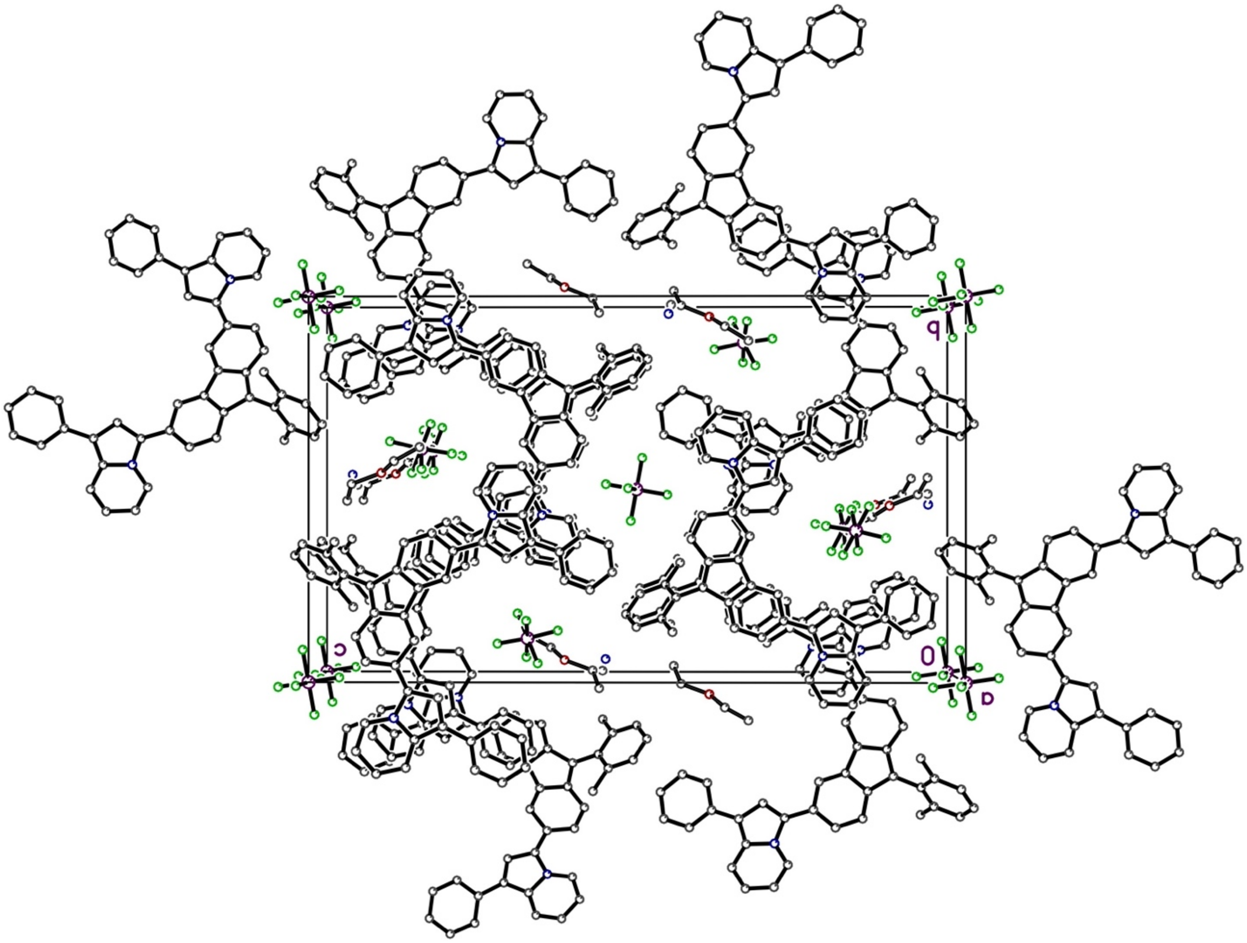

Figure S24. Perspective of the crystal packing structure for **[1Ph]FluIndz**.

Table S10. Atomic coordinates for crystallography of **^{1Ph}FluIndz**.

Number	Label	Xfrac + ESD	Yfrac + ESD	Zfrac + ESD	Symm. op.
1	N1	0.8485(3)	0.06539(16)	0.21431(8)	x,y,z
2	N3	1.1971(4)	0.56761(17)	0.35457(9)	x,y,z
3	C1	0.8399(5)	0.2438(2)	0.39787(10)	x,y,z
4	C2	0.8269(4)	0.2047(2)	0.35804(9)	x,y,z
5	C3	0.7615(4)	0.1360(2)	0.34885(9)	x,y,z
6	H3	0.71754	0.105647	0.370793	x,y,z
7	C4	0.7610(4)	0.1120(2)	0.30702(9)	x,y,z
8	H4	0.717864	0.0647	0.300714	x,y,z
9	C5	0.8228(4)	0.15606(19)	0.27396(9)	x,y,z
10	C6	0.8888(4)	0.2266(2)	0.28362(9)	x,y,z
11	H6	0.930469	0.257518	0.261719	x,y,z
12	C7	0.8914(4)	0.24941(19)	0.32491(9)	x,y,z
13	C8	0.9522(4)	0.3180(2)	0.34453(9)	x,y,z
14	C9	1.0236(4)	0.3791(2)	0.32804(10)	x,y,z
15	H9	1.045091	0.381985	0.298461	x,y,z
16	C10	1.0676(4)	0.4402(2)	0.35508(10)	x,y,z
17	C11	1.0338(5)	0.4321(2)	0.39966(10)	x,y,z
18	H11	1.0645	0.470829	0.418222	x,y,z
19	C12	0.9595(5)	0.3709(2)	0.41623(10)	x,y,z
20	H12	0.93623	0.368148	0.445737	x,y,z
21	C13	0.9167(4)	0.3114(2)	0.38960(9)	x,y,z
22	C14	0.7719(5)	0.2175(2)	0.43922(9)	x,y,z
23	C15	0.8680(5)	0.1627(2)	0.46142(10)	x,y,z
24	C16	0.8005(6)	0.1396(2)	0.50026(11)	x,y,z
25	H16	0.864232	0.103024	0.515775	x,y,z
26	C17	0.6396(6)	0.1701(2)	0.51642(11)	x,y,z
27	H17	0.594645	0.154028	0.542944	x,y,z
28	C18	0.5459(5)	0.2228(2)	0.49448(11)	x,y,z
29	H18	0.436286	0.242705	0.505987	x,y,z
30	C19	0.6087(5)	0.2480(2)	0.45533(10)	x,y,z
31	C20	1.0461(5)	0.1320(2)	0.44545(12)	x,y,z
32	H20A	1.094832	0.096595	0.465888	x,y,z
33	H20B	1.13636	0.170927	0.441954	x,y,z
34	H20C	1.023216	0.108225	0.418086	x,y,z
35	C21	0.5011(5)	0.3048(2)	0.43175(11)	x,y,z
36	H21A	0.375953	0.307911	0.443165	x,y,z
37	H21B	0.493469	0.292021	0.401618	x,y,z

38	H21C	0.563442	0.351501	0.435036	x,y,z
39	C22	0.8128(4)	0.1343(2)	0.22942(9)	x,y,z
40	C23	0.7595(4)	0.1759(2)	0.19512(9)	x,y,z
41	H23	0.72666	0.2255	0.196457	x,y,z
42	C24	0.7609(4)	0.1343(2)	0.15787(9)	x,y,z
43	C25	0.8209(4)	0.06497(19)	0.16979(9)	x,y,z
44	C26	0.8645(4)	0.0002(2)	0.14821(10)	x,y,z
45	H26	0.850984	-0.001868	0.118223	x,y,z
46	C27	0.9253(5)	-0.0588(2)	0.1698(1)	x,y,z
47	H27	0.952488	-0.101994	0.154926	x,y,z
48	C28	0.9485(5)	-0.0561(2)	0.21468(10)	x,y,z
49	H28	0.990195	-0.097552	0.229783	x,y,z
50	C29	0.9113(5)	0.0053(2)	0.23561(10)	x,y,z
51	H29	0.928704	0.007169	0.26551	x,y,z
52	C30	0.7007(4)	0.1602(2)	0.11562(9)	x,y,z
53	C31	0.6742(7)	0.1162(2)	0.08129(12)	x,y,z
54	H31	0.699661	0.066096	0.08423	x,y,z
55	C32	0.6110(6)	0.1420(3)	0.04197(12)	x,y,z
56	H32	0.595269	0.109632	0.018838	x,y,z
57	C33	0.5719(5)	0.2134(2)	0.03670(11)	x,y,z
58	H33	0.525666	0.231335	0.010364	x,y,z
59	C34	0.6009(10)	0.2583(3)	0.07032(15)	x,y,z
60	H34	0.57514	0.308316	0.067199	x,y,z
61	C35	0.6666(9)	0.2330(3)	0.10865(14)	x,y,z
62	H35	0.689361	0.266405	0.131029	x,y,z
63	C36	1.1266(4)	0.5036(2)	0.3352(1)	x,y,z
64	C37	1.1132(5)	0.5204(2)	0.29107(10)	x,y,z
65	H37	1.075099	0.487234	0.269653	x,y,z
66	C38	1.1622(4)	0.5898(2)	0.28382(11)	x,y,z
67	C39	1.2164(5)	0.6208(2)	0.32386(11)	x,y,z
68	C40	1.2869(5)	0.6876(2)	0.33521(13)	x,y,z
69	H40	1.300475	0.724322	0.314469	x,y,z
70	C41	1.3374(5)	0.7005(3)	0.37689(13)	x,y,z
71	H41	1.380027	0.746952	0.385267	x,y,z
72	C42	1.3258(5)	0.6454(2)	0.40654(12)	x,y,z
73	H42	1.364074	0.653955	0.435065	x,y,z
74	C43	1.2600(5)	0.5791(2)	0.39506(11)	x,y,z
75	H43	1.257924	0.540938	0.415195	x,y,z
76	C44	1.1685(5)	0.6266(2)	0.24219(11)	x,y,z

77	C45	1.1283(5)	0.6997(2)	0.23680(13)	x,y,z
78	H45	1.094426	0.728349	0.260409	x,y,z
79	C46	1.1380(5)	0.7303(3)	0.19673(14)	x,y,z
80	H46	1.110563	0.78021	0.193312	x,y,z
81	C47	1.1861(5)	0.6906(3)	0.16205(13)	x,y,z
82	H47	1.193293	0.71264	0.134926	x,y,z
83	C48	1.2240(5)	0.6181(3)	0.16704(12)	x,y,z
84	H48	1.256287	0.589813	0.143141	x,y,z
85	C49	1.2154(5)	0.5863(3)	0.20665(11)	x,y,z
86	H49	1.241814	0.536232	0.209658	x,y,z
87	P1	0.5	0.5	0.5	x,y,z
88	F1	0.5526(6)	0.48597(16)	0.45188(7)	x,y,z
89	F2	0.5019(5)	0.58408(15)	0.49103(8)	x,y,z
90	F3	0.2857(5)	0.49692(19)	0.48721(10)	x,y,z
91	F1	0.4474(6)	0.51403(16)	0.54812(7)	1-x,1-y,1-z
92	F2	0.4981(5)	0.41592(15)	0.50897(8)	1-x,1-y,1-z
93	F3	0.7143(5)	0.50308(19)	0.51279(10)	1-x,1-y,1-z
94	P2	0.1454(4)	0.39384(15)	0.16934(8)	x,y,z
95	F4	-0.0196(12)	0.4475(6)	0.1728(3)	x,y,z
96	F5	0.2079(12)	0.4064(4)	0.2154(2)	x,y,z
97	F6	0.0137(9)	0.3304(3)	0.1863(2)	x,y,z
98	F7	0.3133(11)	0.3443(5)	0.1698(4)	x,y,z
99	F8	0.081(2)	0.3748(4)	0.1227(2)	x,y,z
100	F9	0.2698(15)	0.4565(4)	0.1525(3)	x,y,z
101	C1E	0.800(3)	0.3916(8)	0.1733(4)	x,y,z
102	H1EA	0.694652	0.371894	0.188611	x,y,z
103	H1EB	0.883113	0.352126	0.165269	x,y,z
104	H1EC	0.867788	0.425736	0.191578	x,y,z
105	C2E	0.736(2)	0.4278(8)	0.1363(5)	x,y,z
106	H2EA	0.651357	0.467641	0.144041	x,y,z
107	H2EB	0.666739	0.393755	0.117635	x,y,z
108	O1E	0.9031(13)	0.4575(5)	0.1134(2)	x,y,z
109	C3E	0.860(3)	0.4863(12)	0.0693(4)	x,y,z
110	H3EA	0.84434	0.446066	0.048845	x,y,z
111	H3EB	0.745856	0.515654	0.069147	x,y,z
112	C4E	1.016(5)	0.530(2)	0.0585(12)	x,y,z
113	H4EA	0.99668	0.54987	0.030058	x,y,z
114	H4EB	1.030123	0.568885	0.079084	x,y,z
115	H4EC	1.128044	0.499695	0.058898	x,y,z

116	C1S	1.030(5)	0.521(2)	0.0614(12)	x,y,z
117	H1SA	1.068003	0.512642	0.090962	x,y,z
118	H1SB	1.023767	0.572663	0.055712	x,y,z
119	H1SC	1.119402	0.498166	0.042542	x,y,z
120	C2S	0.842(3)	0.4877(12)	0.0537(5)	x,y,z
121	N1S	0.7037(13)	0.4599(5)	0.0493(3)	x,y,z

Table S11. Bond lengths for crystallography of **^{1Ph}FluIndz**.

Number	Atom1	Atom2	Type	Polymeric	Cyclicity	Length	SybylType
1	N1	C22	Unknown	no	cyclic	1.388(5)	1
2	N1	C25	Unknown	no	cyclic	1.415(4)	1
3	N1	C29	Unknown	no	cyclic	1.373(5)	1
4	N3	C36	Unknown	no	cyclic	1.423(5)	1
5	N3	C39	Unknown	no	cyclic	1.390(5)	1
6	N3	C43	Unknown	no	cyclic	1.365(4)	1
7	C1	C2	Unknown	no	cyclic	1.452(5)	un
8	C1	C13	Unknown	no	cyclic	1.395(5)	un
9	C1	C14	Unknown	no	acyclic	1.483(4)	un
10	C2	C3	Unknown	no	cyclic	1.386(5)	un
11	C2	C7	Unknown	no	cyclic	1.418(4)	un
12	C3	H3	Unknown	no	acyclic	0.95	1
13	C3	C4	Unknown	no	cyclic	1.392(4)	un
14	C4	H4	Unknown	no	acyclic	0.949	1
15	C4	C5	Unknown	no	cyclic	1.403(4)	un
16	C5	C6	Unknown	no	cyclic	1.421(5)	un
17	C5	C22	Unknown	no	acyclic	1.462(4)	un
18	C6	H6	Unknown	no	acyclic	0.95	1
19	C6	C7	Unknown	no	cyclic	1.369(4)	un
20	C7	C8	Unknown	no	cyclic	1.476(5)	un
21	C8	C9	Unknown	no	cyclic	1.351(5)	un
22	C8	C13	Unknown	no	cyclic	1.454(4)	un
23	C9	H9	Unknown	no	acyclic	0.95	1
24	C9	C10	Unknown	no	cyclic	1.449(5)	un
25	C10	C11	Unknown	no	cyclic	1.439(4)	un
26	C10	C36	Unknown	no	acyclic	1.400(5)	un
27	C11	H11	Unknown	no	acyclic	0.95	1
28	C11	C12	Unknown	no	cyclic	1.362(5)	un
29	C12	H12	Unknown	no	acyclic	0.95	1
30	C12	C13	Unknown	no	cyclic	1.416(5)	un

31	C14	C15	Unknown	no	cyclic	1.409(5)	un
32	C14	C19	Unknown	no	cyclic	1.409(5)	un
33	C15	C16	Unknown	no	cyclic	1.393(5)	un
34	C15	C20	Unknown	no	acyclic	1.501(5)	1
35	C16	H16	Unknown	no	acyclic	0.95	1
36	C16	C17	Unknown	no	cyclic	1.396(6)	un
37	C17	H17	Unknown	no	acyclic	0.95	1
38	C17	C18	Unknown	no	cyclic	1.369(5)	un
39	C18	H18	Unknown	no	acyclic	0.951	1
40	C18	C19	Unknown	no	cyclic	1.402(5)	un
41	C19	C21	Unknown	no	acyclic	1.498(5)	1
42	C20	H20A	Unknown	no	acyclic	0.981	1
43	C20	H20B	Unknown	no	acyclic	0.979	1
44	C20	H20C	Unknown	no	acyclic	0.98	1
45	C21	H21A	Unknown	no	acyclic	0.98	1
46	C21	H21B	Unknown	no	acyclic	0.98	1
47	C21	H21C	Unknown	no	acyclic	0.979	1
48	C22	C23	Unknown	no	cyclic	1.378(4)	un
49	C23	H23	Unknown	no	acyclic	0.949	1
50	C23	C24	Unknown	no	cyclic	1.405(4)	un
51	C24	C25	Unknown	no	cyclic	1.404(5)	un
52	C24	C30	Unknown	no	acyclic	1.475(4)	un
53	C25	C26	Unknown	no	cyclic	1.417(5)	un
54	C26	H26	Unknown	no	acyclic	0.95	1
55	C26	C27	Unknown	no	cyclic	1.356(5)	un
56	C27	H27	Unknown	no	acyclic	0.949	1
57	C27	C28	Unknown	no	cyclic	1.424(4)	un
58	C28	H28	Unknown	no	acyclic	0.95	1
59	C28	C29	Unknown	no	cyclic	1.344(5)	un
60	C29	H29	Unknown	no	acyclic	0.95	1
61	C30	C31	Unknown	no	cyclic	1.366(5)	un
62	C30	C35	Unknown	no	cyclic	1.387(7)	un
63	C31	H31	Unknown	no	acyclic	0.95	1
64	C31	C32	Unknown	no	cyclic	1.398(6)	un
65	C32	H32	Unknown	no	acyclic	0.95	1
66	C32	C33	Unknown	no	cyclic	1.361(7)	un
67	C33	H33	Unknown	no	acyclic	0.95	1
68	C33	C34	Unknown	no	cyclic	1.361(6)	un
69	C34	H34	Unknown	no	acyclic	0.949	1

70	C34	C35	Unknown	no	cyclic	1.374(7)	un
71	C35	H35	Unknown	no	acyclic	0.951	1
72	C36	C37	Unknown	no	cyclic	1.428(4)	un
73	C37	H37	Unknown	no	acyclic	0.95	1
74	C37	C38	Unknown	no	cyclic	1.353(5)	un
75	C38	C39	Unknown	no	cyclic	1.436(5)	un
76	C38	C44	Unknown	no	acyclic	1.481(5)	un
77	C39	C40	Unknown	no	cyclic	1.382(5)	un
78	C40	H40	Unknown	no	acyclic	0.95	1
79	C40	C41	Unknown	no	cyclic	1.379(6)	un
80	C41	H41	Unknown	no	acyclic	0.949	1
81	C41	C42	Unknown	no	cyclic	1.387(6)	un
82	C42	H42	Unknown	no	acyclic	0.95	1
83	C42	C43	Unknown	no	cyclic	1.363(5)	un
84	C43	H43	Unknown	no	acyclic	0.95	1
85	C44	C45	Unknown	no	cyclic	1.394(5)	un
86	C44	C49	Unknown	no	cyclic	1.393(5)	un
87	C45	H45	Unknown	no	acyclic	0.95	1
88	C45	C46	Unknown	no	cyclic	1.388(6)	un
89	C46	H46	Unknown	no	acyclic	0.951	1
90	C46	C47	Unknown	no	cyclic	1.368(7)	un
91	C47	H47	Unknown	no	acyclic	0.95	1
92	C47	C48	Unknown	no	cyclic	1.378(8)	un
93	C48	H48	Unknown	no	acyclic	0.95	1
94	C48	C49	Unknown	no	cyclic	1.383(6)	un
95	C49	H49	Unknown	no	acyclic	0.951	1

Table S12. Bond angles for crystallography of ^{1Ph}**FluIndz**.

Number	Atom1	Atom2	Atom3	Angle
1	C22	N1	C25	108.8(2)
2	C22	N1	C29	129.7(3)
3	C25	N1	C29	121.4(3)
4	C36	N3	C39	109.3(3)
5	C36	N3	C43	130.0(3)
6	C39	N3	C43	120.4(3)
7	C2	C1	C13	107.8(3)
8	C2	C1	C14	125.4(3)
9	C13	C1	C14	126.7(3)
10	C1	C2	C3	131.0(3)

11	C1	C2	C7	109.2(3)
12	C3	C2	C7	119.8(3)
13	C2	C3	H3	120.5
14	C2	C3	C4	119.1(3)
15	H3	C3	C4	120.4
16	C3	C4	H4	119.2
17	C3	C4	C5	121.5(3)
18	H4	C4	C5	119.2
19	C4	C5	C6	119.0(3)
20	C4	C5	C22	122.9(3)
21	C6	C5	C22	118.0(3)
22	C5	C6	H6	120.5
23	C5	C6	C7	119.1(3)
24	H6	C6	C7	120.4
25	C2	C7	C6	121.4(3)
26	C2	C7	C8	107.0(3)
27	C6	C7	C8	131.6(3)
28	C7	C8	C9	132.2(3)
29	C7	C8	C13	106.4(3)
30	C9	C8	C13	121.4(3)
31	C8	C9	H9	119.7
32	C8	C9	C10	120.6(3)
33	H9	C9	C10	119.8
34	C9	C10	C11	117.1(3)
35	C9	C10	C36	117.1(3)
36	C11	C10	C36	125.6(3)
37	C10	C11	H11	118.8
38	C10	C11	C12	122.4(3)
39	H11	C11	C12	118.8
40	C11	C12	H12	119.9
41	C11	C12	C13	120.2(3)
42	H12	C12	C13	119.9
43	C1	C13	C8	109.6(3)
44	C1	C13	C12	132.1(3)
45	C8	C13	C12	118.3(3)
46	C1	C14	C15	120.4(3)
47	C1	C14	C19	118.5(3)
48	C15	C14	C19	121.1(3)
49	C14	C15	C16	118.8(3)

50	C14	C15	C20	121.6(3)
51	C16	C15	C20	119.6(3)
52	C15	C16	H16	119.9
53	C15	C16	C17	120.2(3)
54	H16	C16	C17	120
55	C16	C17	H17	119.6
56	C16	C17	C18	120.7(4)
57	H17	C17	C18	119.7
58	C17	C18	H18	119.4
59	C17	C18	C19	121.2(3)
60	H18	C18	C19	119.4
61	C14	C19	C18	118.0(3)
62	C14	C19	C21	122.2(3)
63	C18	C19	C21	119.9(3)
64	C15	C20	H20A	109.4
65	C15	C20	H20B	109.6
66	C15	C20	H20C	109.4
67	H20A	C20	H20B	109.5
68	H20A	C20	H20C	109.4
69	H20B	C20	H20C	109.5
70	C19	C21	H21A	109.4
71	C19	C21	H21B	109.4
72	C19	C21	H21C	109.5
73	H21A	C21	H21B	109.5
74	H21A	C21	H21C	109.5
75	H21B	C21	H21C	109.5
76	N1	C22	C5	125.2(3)
77	N1	C22	C23	107.2(3)
78	C5	C22	C23	127.5(3)
79	C22	C23	H23	124.9
80	C22	C23	C24	110.2(3)
81	H23	C23	C24	124.9
82	C23	C24	C25	106.5(3)
83	C23	C24	C30	124.8(3)
84	C25	C24	C30	128.6(3)
85	N1	C25	C24	107.4(3)
86	N1	C25	C26	116.9(3)
87	C24	C25	C26	135.7(3)
88	C25	C26	H26	119.5

89	C25	C26	C27	120.9(3)
90	H26	C26	C27	119.6
91	C26	C27	H27	119.9
92	C26	C27	C28	120.2(3)
93	H27	C27	C28	119.9
94	C27	C28	H28	120.1
95	C27	C28	C29	119.8(3)
96	H28	C28	C29	120.1
97	N1	C29	C28	120.8(3)
98	N1	C29	H29	119.6
99	C28	C29	H29	119.6
100	C24	C30	C31	123.9(3)
101	C24	C30	C35	120.5(3)
102	C31	C30	C35	115.6(4)
103	C30	C31	H31	118.7
104	C30	C31	C32	122.6(4)
105	H31	C31	C32	118.7
106	C31	C32	H32	119.9
107	C31	C32	C33	120.3(4)
108	H32	C32	C33	119.8
109	C32	C33	H33	121
110	C32	C33	C34	118.0(4)
111	H33	C33	C34	121
112	C33	C34	H34	119.2
113	C33	C34	C35	121.6(5)
114	H34	C34	C35	119.2
115	C30	C35	C34	121.9(5)
116	C30	C35	H35	119.1
117	C34	C35	H35	119
118	N3	C36	C10	127.9(3)
119	N3	C36	C37	104.8(3)
120	C10	C36	C37	127.1(3)
121	C36	C37	H37	124.5
122	C36	C37	C38	111.0(3)
123	H37	C37	C38	124.5
124	C37	C38	C39	107.4(3)
125	C37	C38	C44	126.8(3)
126	C39	C38	C44	125.8(3)
127	N3	C39	C38	107.5(3)

128	N3	C39	C40	119.6(3)
129	C38	C39	C40	132.8(3)
130	C39	C40	H40	120.3
131	C39	C40	C41	119.5(4)
132	H40	C40	C41	120.3
133	C40	C41	H41	120.1
134	C40	C41	C42	119.8(4)
135	H41	C41	C42	120.1
136	C41	C42	H42	119.7
137	C41	C42	C43	120.5(4)
138	H42	C42	C43	119.8
139	N3	C43	C42	119.9(3)
140	N3	C43	H43	120
141	C42	C43	H43	120.1
142	C38	C44	C45	123.1(3)
143	C38	C44	C49	118.6(3)
144	C45	C44	C49	118.3(3)
145	C44	C45	H45	120.2
146	C44	C45	C46	119.6(4)
147	H45	C45	C46	120.2
148	C45	C46	H46	119.1
149	C45	C46	C47	121.8(4)
150	H46	C46	C47	119.1
151	C46	C47	H47	120.5
152	C46	C47	C48	119.0(4)
153	H47	C47	C48	120.5
154	C47	C48	H48	119.8
155	C47	C48	C49	120.4(4)
156	H48	C48	C49	119.8
157	C44	C49	C48	120.9(4)
158	C44	C49	H49	119.5
159	C48	C49	H49	119.5
160	F1	P1	F2	89.3
161	F1	P1	F3	89.9
162	F1	P1	F1	180
163	F1	P1	F2	90.7
164	F1	P1	F3	90.1
165	F2	P1	F3	90.1
166	F2	P1	F1	90.7

167	F2	P1	F2	180
168	F2	P1	F3	89.9
169	F3	P1	F1	90.1
170	F3	P1	F2	89.9
171	F3	P1	F3	180
172	F1	P1	F2	89.3
173	F1	P1	F3	89.9
174	F2	P1	F3	90.1
175	F4	P2	F5	92.9(5)
176	F4	P2	F6	89.2(4)
177	F4	P2	F7	174.7(6)
178	F4	P2	F8	89.7(5)
179	F4	P2	F9	89.7(5)
180	F5	P2	F6	87.8(4)
181	F5	P2	F7	82.0(5)
182	F5	P2	F8	175.8(5)
183	F5	P2	F9	92.7(5)
184	F6	P2	F7	91.8(5)
185	F6	P2	F8	88.9(4)
186	F6	P2	F9	178.8(4)
187	F7	P2	F8	95.6(5)
188	F7	P2	F9	89.3(5)
189	F8	P2	F9	90.6(5)

PARAMAGNETIC EXPERIMENTS

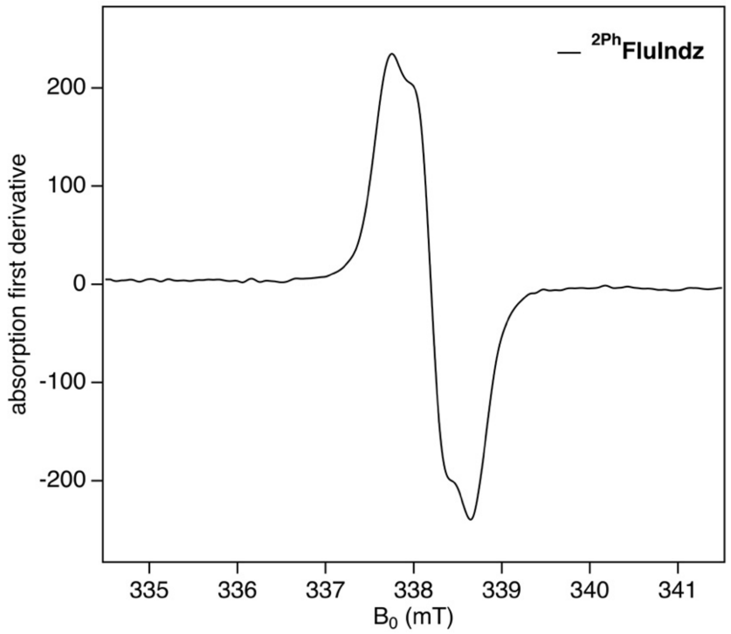

Figure S25. X-band (9.5 GHz) EPR spectrum of **^{2Ph}FluIndz** collected in DCM solution at room temperature, g = 1.9991.

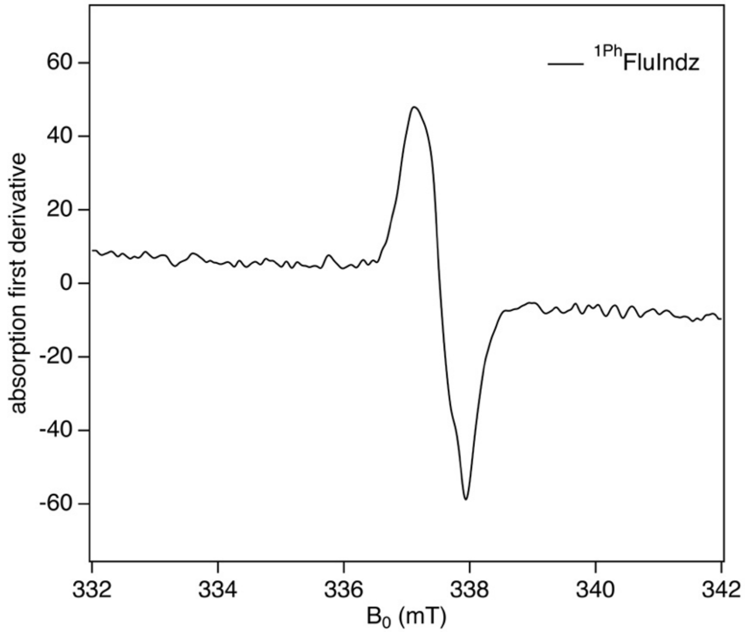

Figure S26. X-band (9.5 GHz) EPR spectrum of **^{1Ph}FluIndz** collected in DCM solution at room temperature, g = 2.0035.

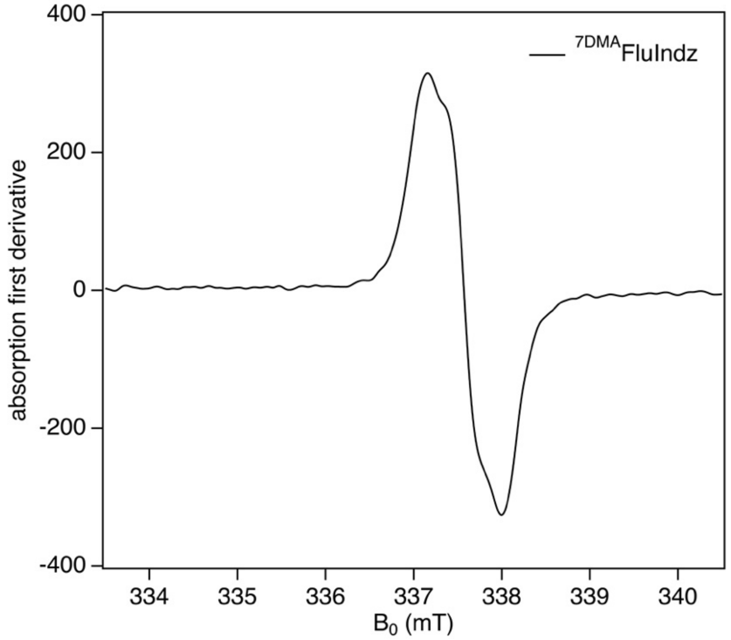

Figure S27. X-band (9.5 GHz) EPR spectrum of **[7DMA]FluIndz** collected in DCM solution at room temperature, g = 2.0034.

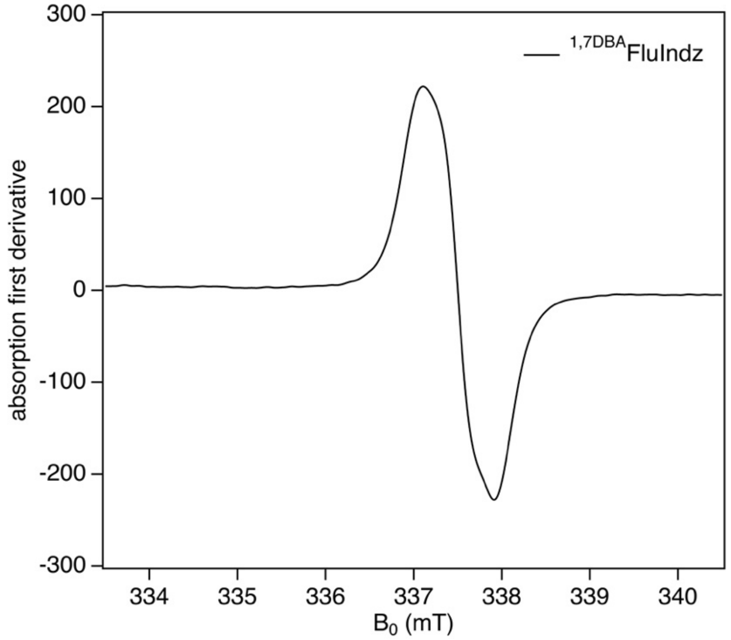

Figure S28. X-band (9.5 GHz) EPR spectrum of **[1,7DBA]FluIndz** collected in DCM solution at room temperature, g = 2.0035.

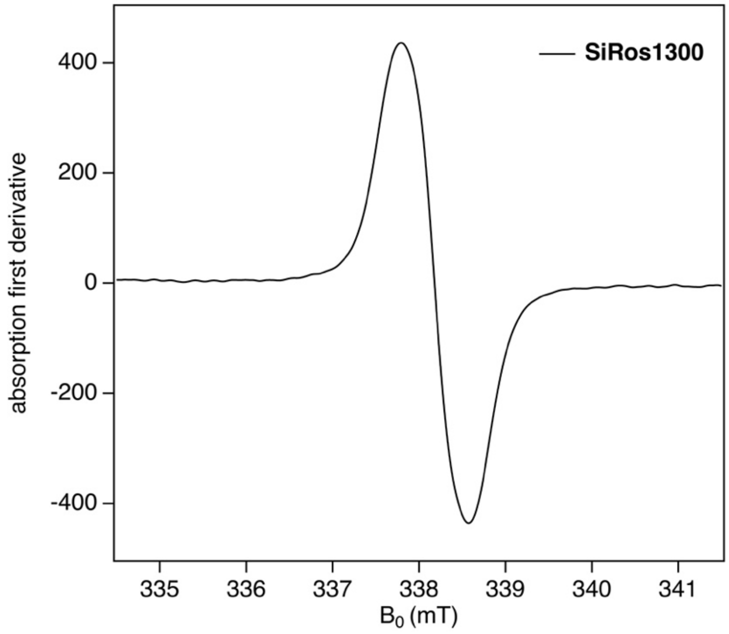

Figure S29. X-band (9.5 GHz) EPR spectrum of **SiRos1300** collected in DCM solution at room temperature, g = 2.0036.

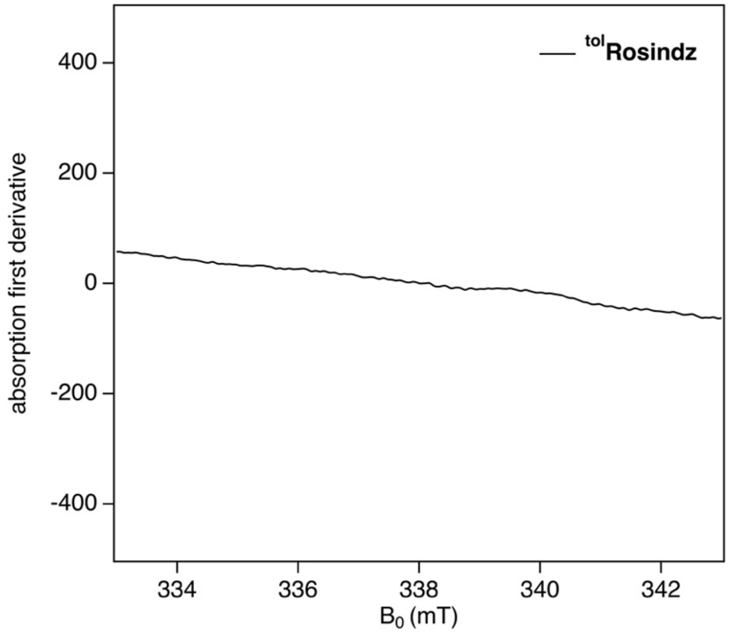

Figure S30. X-band (9.5 GHz) EPR spectrum of **^{tol}RosIndz** collected in DCM solution at room temperature showing no EPR activity.

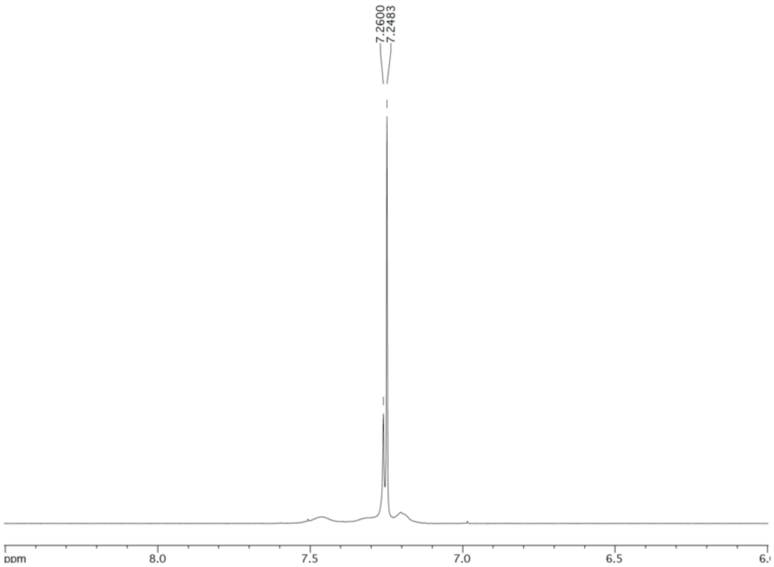

Figure S31. Evans method NMR spectrum for **^{2Ph}FluIndz** in $CDCl_3$ solution. Parameters: T = 298 K, spectrometer frequency = 400 MHz, concentration = 1.71×10^{-2} M. Paramagnetic values calculated: χ_m (magnetic susceptibility) = 2.053×10^{-9} m^3/mol, μ_{eff} (effective magnetic moment) = 0.624 μ_B, n (number of unpaired electrons per molecule for spin-only μ_{eff}) = 0.179.

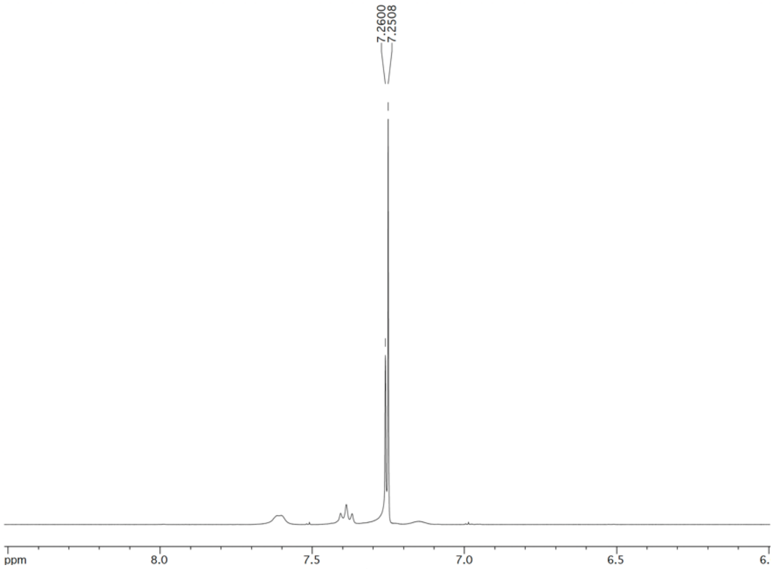

Figure S32. Evans method NMR spectrum for **^{1Ph}FluIndz** in $CDCl_3$ solution. Parameters: T = 298 K, spectrometer frequency = 400 MHz, concentration = 9.94×10^{-3} M. Paramagnetic values calculated: χ_m (magnetic susceptibility) = 2.777×10^{-9} m^3/mol, μ_{eff} (effective magnetic moment) = 0.726 μ_B, n (number of unpaired electrons per molecule for spin-only μ_{eff}) = 0.236.

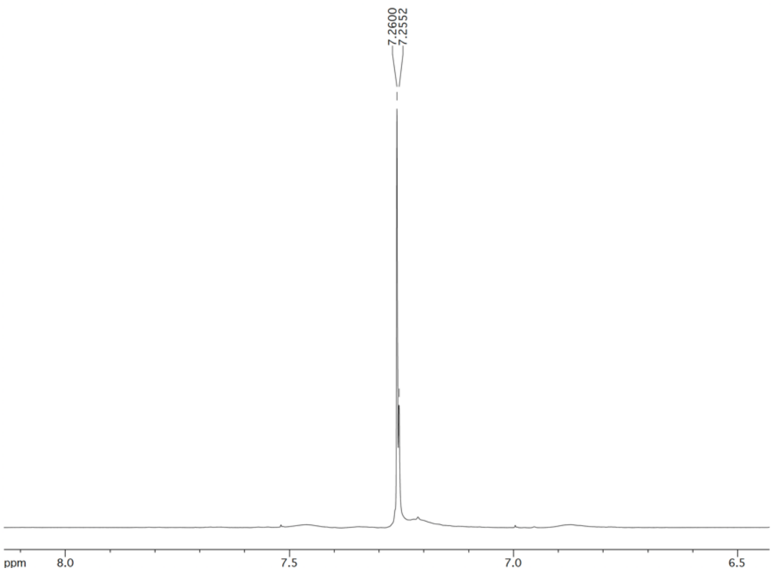

Figure S33. Evans method NMR spectrum for **^{7DMA}FluIndz** in $CDCl_3$ solution. Parameters: T = 298 K, spectrometer frequency = 400 MHz, concentration = 1.662×10^{-2} M. Paramagnetic values calculated: χ_m (magnetic susceptibility) = 8.659×10^{-10} m^3/mol, μ_{eff} (effective magnetic moment) = 0.405 μ_B, n (number of unpaired electrons per molecule for spin-only μ_{eff}) = 0.079.

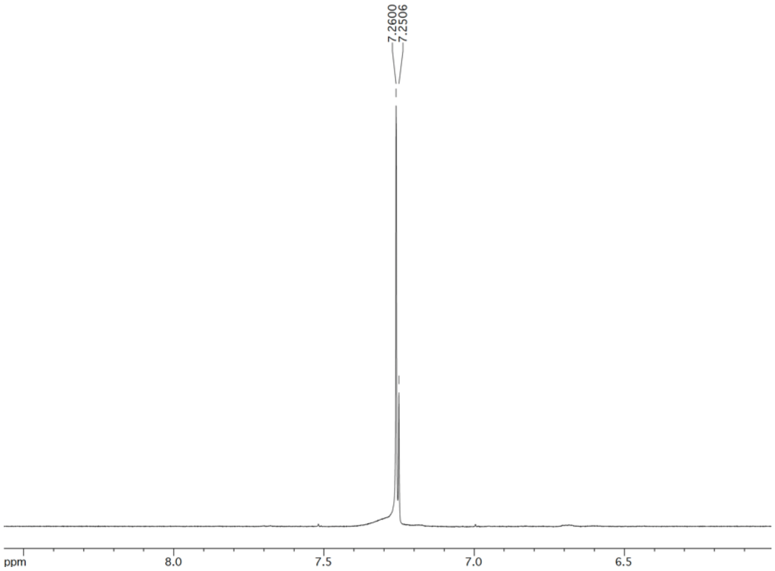

Figure S34. Evans method NMR spectrum for **^{1,7DBA}FluIndz** in $CDCl_3$ solution. Parameters: T = 298 K, spectrometer frequency = 400 MHz, concentration = 8.535×10^{-3} M. Paramagnetic values calculated: χ_m (magnetic susceptibility) = 3.304×10^{-9} m^3/mol, μ_{eff} (effective magnetic moment) = 0.792 μ_B, n (number of unpaired electrons per molecule for spin-only μ_{eff}) = 0.275.

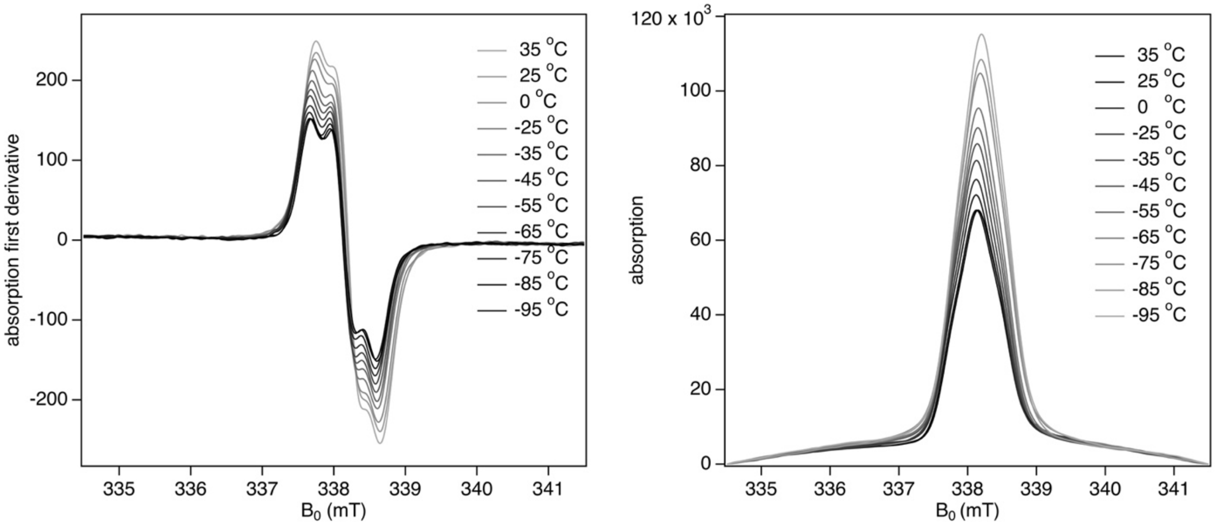

Figure S35. Variable temperature EPR experiment including the first derivative and integrated absorption of **^{2Ph}FluIndz** in DCM solution from 30 °C down to -95 °C.

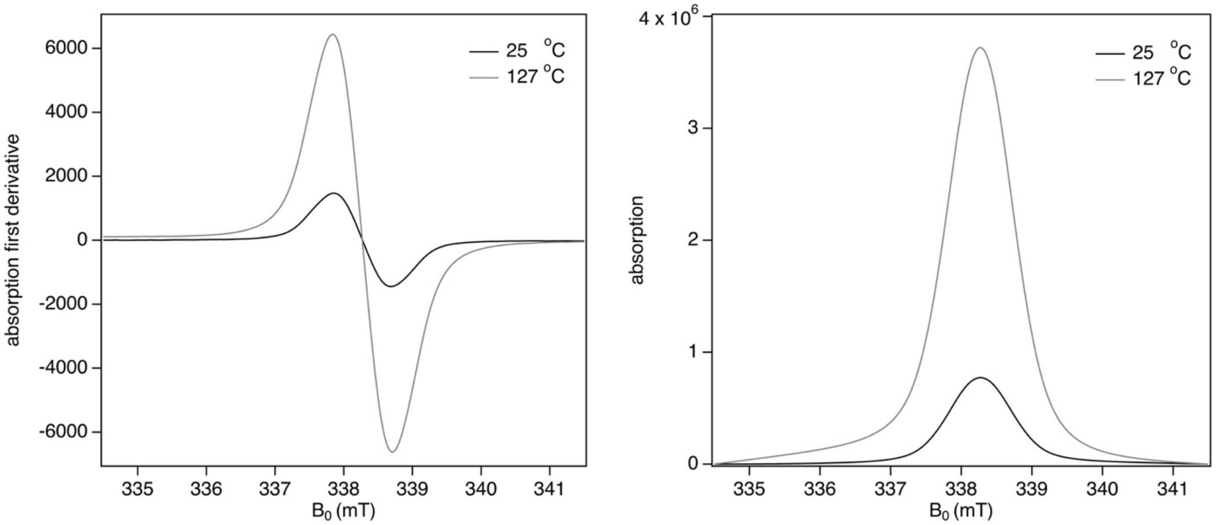

Figure S36. Variable temperature EPR experiment including the first derivative and integrated absorption of **^{2Ph}FluIndz** in PhCl solution from 25 °C (g = 2.0034) up to 127°C (g = 2.0035).

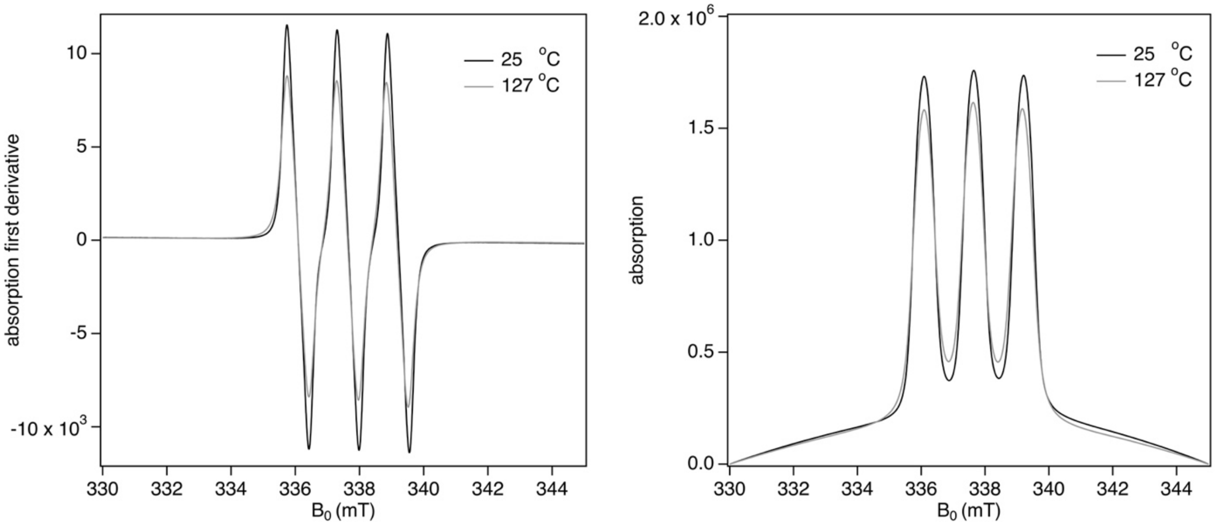

Figure S37. Variable temperature EPR experiment including the first derivative and integrated absorption of TEMPO in PhCl solution from 25 °C up to 127°C as an example of a temperature independent radical EPR response.

SYNTHESIS

3,6-bis(1-methyl-2-phenylindolizin-3-yl)-9H-fluoren-9-one (**6**): To a flame dried pressure flask under N_2 was added 1-methyl-2-phenylindolizine (**2**, 1.30 g, 6.27 mmol), 3,6-dibromo-9*H*-fluoren-9-one (**1**, 0.922 g, 2.73 mmol), $Pd(OAc)_2$ (61.4 mg, 0.273 mmol), PCy_3HBF_4 (201 mg, 0.546 mmol), Cs_2CO_3 (5.34 g, 16.4 mmol), and anhydrous toluene (11.0 mL). The reaction was degassed with N_2 for ~15 minutes before sealing and heating to 120°C for 16 hours. At this point, the reaction was observed to be complete via ^{1}H NMR monitoring. The reaction was cooled to room temperature and transferred to a separatory funnel containing water and DCM, shaken vigorously, and the organic layer collected. The aqueous layer was washed three more times with DCM. The organic layers were combined, dried over Na_2SO_4, and concentrated to dryness to yield the crude product. The crude was subjected to silica gel chromatography using 1:1 DCM:hexanes and the product was observed to elute as a red solution and yielded a matte red solid when concentrated (0.944 g, 1.60 mmol, 59%). ^{1}H-NMR (400 MHz; $CDCl_3$): δ 8.13 (d, *J* = 6.8 Hz, 2H), 7.58 (d, *J* = 7.7 Hz, 2H), 7.42 (d, *J* = 9.0 Hz, 2H), 7.33-7.28 (ms, 8H), 7.24-7.22 (ms, 4H), 7.16 (dd, *J* = 7.7, 1.3 Hz, 2H), 6.75 (t, *J* = 7.6 Hz, 2H), 6.51 (t, *J* = 6.7 Hz, 2H), 2.34 (s, 6H). ^{13}C NMR were taken in both DMSO-d_6 and $CDCl_3$ due to insufficient resolution in a single solvent despite both samples being saturated and run for 16 hours. In DMSO-d_6 all of the aromatic carbon signals are observed, however, the ketone carbon signal is not observed. In $CDCl_3$ the ketone carbon signal is observed, but not all of the aromatic carbon signals are observed. ^{13}C NMR (101 MHz; DMSO-d_6): δ 143.9, 137.5, 134.7, 131.7, 130.9, 130.8, 130.3, 128.7, 128.2, 126.6, 124.2, 122.4, 121.9, 120.1, 117.7, 117.5, 111.4, 107.5, 9.0. ^{13}C NMR (101 MHz; $CDCl_3$): δ 192.4, 144.5, 138.2, 135.1, 132.9, 130.7, 130.6, 130.6, 128.4, 126.9, 126.9, 124.8, 121.3, 118.1, 9.4. HRMS *m/z* calculated for $C_{43}H_{30}N_2O$ $[M]^+$ 590.2358, found 590.2330. IR (cm^{-1}) 3107, 3054, 2921, 2855, 1701, 1606, 1524, 1495, 1446, 1422, 1394, 1350, 1323, 1295, 1264, 1248, 1234, 1195, 1154,1124, 1105, 1065, 1032, 1014, 1003.

3,6-bis(1-phenylindolizin-3-yl)-9H-fluoren-9-one (**7**): To a flame dried pressure flask under N_2 was added 1-phenylindolizine (**3**, 0.72 g, 3.73 mmol), 3,6-dibromo-9*H*-fluoren-9-one (**1**, 0.504 g, 1.49 mmol), $Pd(OAc)_2$ (33.3 mg, 0.149 mmol), PCy_3HBF_4 (110 mg, 0.298 mmol), Cs_2CO_3 (2.91 g, 8.94 mmol), and anhydrous toluene (11.9 mL). The reaction was degassed with N_2 for ~15 minutes before sealing and heating to 120°C for 16 hours. At this point, the reaction was observed to be complete via ^{1}H NMR monitoring. The reaction was cooled to room temperature and transferred to a separatory funnel containing water and DCM, shaken vigorously, and the organic layer collected. The aqueous layer was washed three more times with DCM. The organic layers were combined, dried over Na_2SO_4, and concentrated to dryness to yield the crude. The crude was subjected to silica gel chromatography using 70:30 DCM:hexanes and the product was observed to elute as a red solution and yielded a red solid when concentrated (0.148 g, 0.263 mmol, 18%). ^{1}H NMR (400 MHz, $CDCl_3$) δ 8.43 (d, *J* = 7.2 Hz, 2H), 7.84 – 7.80 (m, 6H), 7.63 (m, 6H), 7.46 (t, *J* = 7.7 Hz, 4H), 7.29 (t, *J* = 7.4 Hz, 2H), 7.21 (s, 2H), 6.84 (t, *J* = 6.6 Hz, 2H), 6.64 (t, *J* = 6.8 Hz, 2H) ppm. ^{13}C NMR (101 MHz, $CDCl_3$) δ 192.3, 145.0, 138.5, 135.8, 133.1, 131.9, 129.0, 127.8, 127.7, 126.0, 125.2, 125.1, 123.2, 119.4, 119.3, 119.0, 116.6, 115.3, 112.2 ppm. HRMS *m/z* calculated for $C_{43}H_{30}N_2O$ $[M + H]^+$ 563.2123, found 563.2097. IR (cm^{-1}) 3054, 3028, 2953, 2925, 2854, 2360, 2340, 1700, 1599, 1545, 1511, 1489, 1469, 1441, 1418, 1398, 1365, 1341, 1326, 1299, 1253, 1208, 1139, 1110, 1072, 1031, 1014.

3,6-bis(7-(4-(dimethylamino)phenyl)-1-(2-ethylhexyl)-2-phenylindolizin-3-yl)-9H-fluoren-9-one (**8**): To a flame dried pressure flask under N_2 was added 4-(1-(2-ethylhexyl)-2-phenylindolizin-7-yl)-*N*,*N*-dimethylaniline (**4**, 0.500 g, 1.18 mmol), 3,6-dibromo-9*H*-fluoren-9-one (**1**, 0.181 g, 0.536 mmol), $Pd(OAc)_2$ (12.0 mg, 0.0536 mmol), PCy_3HBF_4 (39.4 mg, 0.107 mmol), Cs_2CO_3 (1.05 g, 3.22 mmol), and anhydrous toluene (4.3 mL). The reaction was degassed with N_2 for ~15 minutes before sealing and heating to 130°C for 60 hours. At this point, the reaction was observed to be complete via ^{1}H NMR monitoring. The reaction was cooled to room temperature and transferred to a separatory funnel containing water and Et_2O, shaken vigorously, and the organic layer collected. The aqueous layer was washed three more times with Et_2O. All the organic layers were combined, dried over Na_2SO_4, and concentrated to dryness to yield the crude material. The crude was subjected to silica gel chromatography using 100% hexanes and increasing to 80% DCM/hexanes where the product was observed to elute as a purple solution and yielded a purple solid when concentrated (0.181 g, 0.177 mmol, 33%). ^{1}H-NMR (400 MHz; C_6D_6): δ 7.96 (d, *J* = 7.4 Hz, 2H), 7.89 (s, 2H), 7.73 (d, *J* = 8.7 Hz, 4H), 7.49 (d, *J* = 7.7 Hz, 2H), 7.42 (d, *J* = 7.2 Hz, 4H), 7.26 (s, 2H), 7.21 (t, *J* = 7.5 Hz, 4H), 7.12 (t, *J* = 7.5 Hz, 2H), 6.93 (d, *J* = 7.8 Hz, 2H), 6.78 (d, *J* = 7.4 Hz, 2H), 6.73 (d, *J* = 8.8 Hz, 4H), 2.95 (m, 4H), 2.57 (s, 12H), 1.65 (m, 2H), 1.35 (ms, 8H), 1.19 - 1.14 (ms, 8H), 0.85 - 0.79 (ms, 14H). ^{13}C-NMR (101 MHz; C_6D_6): δ 150.4, 144.8, 137.9, 136.6, 133.2, 133.2, 131.2, 131.0, 130.9, 130.8, 128.6, 127.4, 127.3, 126.9, 124.7, 123.0, 121.3, 121.2, 113.6, 113.4, 113.4, 111.1, 41.6, 40.2, 33.3, 29.2, 28.7, 26.3, 23.5, 14.4, 11.2. HRMS *m/z* calculated for $C_{73}H_{77}N_4O$ $[M]^+$ 1025.6097, found 1025.6110. IR (cm^{-1}) 3055, 3028, 2954, 2924, 2869, 2854, 2800, 1702, 1607, 1523, 1496, 1480, 1465, 1444, 1422, 1379, 1356, 1297, 1233, 1198, 1167, 1127, 1107, 1059, 1029, 1001.

(4-bromopyridin-2-yl)(4-(dibutylamino)phenyl)methanol (**12**): To a flame dried round bottom flask under N_2 was added THF (50 mL) and magnesium turnings (0.62 g, 25.3 mmol) followed by a flake of iodine. 4-bromo-*N*,*N*-dibutylaniline (5.60 g, 19.7 mmol) was then added to the solution and the reaction flask was adapted to a flame dried reflux condenser under N_2. The reaction was heated to reflux overnight, approximately 16 hours. The next morning the reaction was cooled to room temperature. To a separate flame dried round bottom flask under N_2 was added 4-bromopicolinaldehyde (**10**, 2.62 g, 14.1 mmol) and THF (35 mL). The Grignard reaction was added to the aldehyde solution at 0 °C via canula over ~10 minutes. The reaction was allowed to stir at 0 °C for 1 hour and was allowed to come to room temperature over an additional hour. At this time the reaction was quenched with water and extracted with sat. $NH_4Cl_{(aq)}$ and Et_2O three times. The Et_2O layer was dried with Na_2SO_4 and concentrated to yield a dark brown oil as the crude product. The oil was subjected to silica gel chromatography beginning with 100% hexanes and gradually increasing to 20% EtOAc/hexanes to yield the pure product as a yellow oil (3.22 g, 8.23 mmol, 58%). ^{1}H-NMR (300 MHz; C_6D_6): δ 7.86 (d, *J* = 5.3 Hz, 1H), 7.35 (d, *J* = 1.9 Hz, 1H), 7.29 (d, *J* = 8.6 Hz, 2H), 6.69 (dd, *J* = 5.3, 1.9 Hz, 1H), 6.59 (d, *J* = 8.9 Hz, 2H), 5.67 (d, *J* = 4.2 Hz, 1H), 4.52 (d, *J* = 4.3 Hz, 1H), 3.00 (t, *J* = 7.6 Hz, 4H), 1.37 (p, *J* = 7.6 Hz, 4H), 1.12 (sext, *J* = 7.4 Hz, 4H), 0.81 (t, *J* = 7.3 Hz, 6H). ^{13}C NMR (75 MHz; C_6D_6): δ 165.2, 148.9, 148.3, 133.4, 130.4, 128.6, 125.3, 124.6, 112.3, 75.3, 51.0, 29.7, 20.6, 14.2. HRMS *m/z* calculated for $C_{20}H_{28}BrN_2O$ $[M + H]^+$ 391.1380, found 391.1379. IR (cm^{-1}) 3310 br, 3095, 3074, 3047, 2955, 2929, 2870, 1611, 1570, 1553, 1518, 1461, 1396, 1367, 1285, 1252, 1217, 1181, 1148, 1101,1088, 1046.

4-((4-bromopyridin-2-yl)methyl)-N,N-dibutylaniline (**13**): To a flame dried round bottom flask under N_2 was added **12** (3.22 g, 8.23 mmol), AcOH (35 mL), and $HI_{(aq)}$ (2.7 mL, 57% w/w, 20.6 mmol) and the reaction degassed with N_2 for 10 minutes. At this time the reaction was heated to reflux for 1.5 hours upon which the starting material was observed to be consumed via ^{1}H NMR monitoring. The reaction was cooled to room temperature and quenched with 100 mL of saturated $Na_2SO_{3(aq)}$ and allowed to stir for 10 minutes. Sufficient Na_2CO_3 was then added to neutralize the reaction, which was extracted with DCM three times, dried over Na_2SO_4, and concentrated to yield a dark brown oil. The reaction was purified via silica gel chromatography using 100% hexanes and gradually increasing to 20% Et_2O/hexanes to elute the product as a yellow oil. (2.08g, 5.55 mmol, 67%). Note that the product coeluted with an inseparable impurity and was isolated and used in the subsequent reaction as an impure material. The calculated yield herein is estimated from ^{1}H-NMR integrations. ^{1}H-NMR (300 MHz; C_6D_6): δ 7.92 (d, *J* = 5.2 Hz, 1H), 7.41 (d, *J* = 1.4 Hz, 1H), 7.14 (d, *J* = 8.8 Hz, 2H), 6.96 (dd, *J* = 5.2, 1.6 Hz, 1H), 6.60 (d, *J* = 8.8 Hz, 2H), 3.96 (s, 2H), 3.02 (t, *J* = 7.5 Hz, 4H), 1.40 (p, *J* = 7.6 Hz, 4H), 1.14 (sext, *J* = 7.4 Hz, 4H), 0.81 (t, *J* = 7.3 Hz, 6H). ^{13}C NMR (75 MHz; C_6D_6): δ 163.9, 149.9, 147.4, 132.2, 130.3, 126.2, 124.3, 112.8, 105.8, 51.1, 43.8, 29.8, 20.6, 14.2. HRMS *m/z* calculated for $C_{20}H_{28}BrN_2$ $[M + H]^+$ 375.1431, found 375.1429. IR (cm^{-1}) 3092, 3067, 3037, 3010, 2955, 2929, 2870, 1614, 1591, 1561, 1543, 1518, 1462, 1430, 1376, 1368, 1285, 1254, 1216, 1188, 1148, 1109, 1083, 1008.

N,N-dibutyl-4-(2-(4-(dibutylamino)benzyl)pyridin-4-yl)aniline (**15**): To a round bottom flask was added **13** (1.47 g, 3.91 mmol), *N,N*-dibutyl-4-(4,4,5,5-tetramethyl-1,3,2-dioxaborolan-2-yl)aniline (**14**, 2.76 g, 8.33 mmol), $Pd(PPh_3)_4$ (0.411 g, 0.360 mmol), K_2CO_3 (2.95 g, 21.3 mmol), 1,4-dioxane (23 mL), ethanol (10 mL), and water (3 mL). The solution was degassed with N_2 for 10 minutes, then set to reflux overnight until the starting materials were consumed as visualized *via* TLC and ^{1}H-NMR monitoring. The flask was cooled to room temperature and the reaction mixture was quenched with $NH_4Cl_{(aq)}$ and extracted with DCM three times. The organic layers were combined and dried over Na_2SO_4 and concentrated. The resulting crude oil was purified via silica gel chromatography using 100% hexanes and gradually increasing to 20% EtOAc/hexanes to elute the product as a red oil. (**15**, 2.65 g, 5.31 mmol, 96%). Note that the product coeluted with an inseparable impurity and was isolated and used in the subsequent reaction as an impure material. The calculated yield herein is estimated from ^{1}H-NMR integrations. A small amount of pure material was isolated and used for characterization shown herein. ^{1}H-NMR (300 MHz, $CDCl_3$) δ 8.47 (d, *J* = 5.3 Hz, 1H), 7.48 (d, *J* = 9.0 Hz, 2H), 7.30 – 7.28 (m, 1H), 7.26 – 7.23 (m, 1H), 7.13 (d, *J* = 8.7 Hz, 2H), 6.67 (d, *J* = 9.0 Hz, 2H), 6.59 (d, *J* = 8.8 Hz, 2H), 4.06 (s, 2H), 3.35 – 3.26 (m, 4H), 3.26 – 3.14 (m, 4H), 1.63 – 1.46 (m, 8H), 1.46 – 1.19 (m, 8H), 1.09 – 0.82 (m, 12H). ^{13}C-NMR (75 MHz, $CDCl_3$) δ 162.2, 149.4, 148.7, 146.8, 129.8, 127.8, 126.3, 124.3, 119.5, 117.7, 112.1, 111.7, 50.9, 50.8, 43.9, 29.5, 29.4, 20.4, 20.4, 14.1, 14.0. HRMS (ESI) *m*/*z* calculated for $C_{34}H_{50}N_3$ $[M + H]^+$ 500.4005, found: 500.4009. IR (cm^{-1}) 2955, 2929, 2870, 1611, 1594.

4,4'-(2-phenylindolizine-1,7-diyl)bis(N,N-dibutylaniline) (**5**): To a round bottom flask was added **15** (2.65 g, 5.31 mmol), 2-bromoacetophenone (**16**, 1.32 g, 6.63 mmol), and acetone (27 mL). The reaction was then set to reflux for 48 hours until the starting materials were consumed as visualized *via* TLC and ^{1}H-NMR monitoring, at which point the flask was cooled to room temperature and the acetone was removed *via* constant flow of air. To the flask was then added $NaHCO_3$ (2.23 g, 26.5 mmol), 1,4-dioxane (14 mL), ethanol (28 mL), and water (8 mL). The reaction mixture was degassed with N_2 for 10 minutes and set to reflux overnight until the intermediate was consumed

by TLC and ^{1}H-NMR monitoring. The flask was cooled to room temperature, water was added, and the reaction extracted with DCM three times. The organic layers were combined and dried over Na_2SO_4 and concentrated. The resulting crude oil was purified via neutral alumina chromatography using 100% hexanes and gradually increasing to 20% DCM/hexanes to elute the product as a brown oil (2.36 g, 3.94 mmol, 74%). ^{1}H-NMR (400 MHz, CD_3CN) δ 8.01 (d, *J* = 7.2 Hz, 1H), 7.48 (s, 1H), 7.46 (s, 1H), 7.42 (d, *J* = 8.6 Hz, 2H), 7.35–7.17 (m, 5H), 7.08 (d, *J* = 8.4 Hz, 2H), 6.81 (d, *J* = 6.8 Hz, 1H), 6.68 (d, *J* = 8.7 Hz, 2H), 6.64 (d, *J* = 8.6 Hz, 2H), 3.34–3.21 (m, 8H), 1.60–1.47 (m, 8H), 1.40–1.25 (m, 8H), 0.93 (t, *J* = 7.3 Hz, 12H). ^{13}C-NMR (101 MHz, CD_3CN) δ 148.6, 147.5, 136.9, 132.2, 131.9, 131.0, 129.7, 129.1, 129.0, 127.5, 127.1, 126.5, 126.3, 122.6, 113.3, 112.9, 112.7, 112.5, 111.7, 110.9, 51.3, 51.2, 30.2, 30.1, 20.9, 20.9, 14.3, 14.2. HRMS (ESI) *m/z* calculated for $C_{42}H_{54}N_3$ $[M + H]^+$ 600.4318, found: 600.4315. IR (cm^{-1}) 3043, 2954, 2928, 2869, 1608.

3,6-bis(1,7-bis(4-(dibutylamino)phenyl)-2-phenylindolizin-3-yl)-9H-fluoren-9-one (**9**): To a flame dried pressure flask under N_2 was added 4,4'-(2-phenylindolizine-1,7-diyl)bis(*N*,*N*-dibutylaniline) (**5**, 0.75 g, 1.25 mmol), 3,6-dibromo-9*H*-fluoren-9-one (**1**, 0.192 g, 0.568 mmol), $Pd(OAc)_2$ (12.7 mg, 0.057 mmol), PCy_3HBF_4 (41.8 mg, 0.114 mmol), Cs_2CO_3 (1.11 g, 3.41 mmol), and anhydrous toluene (4.5 mL). The reaction was degassed with N_2 for ~15 minutes before sealing and heating to 130°C for 22 hours. At this point, the reaction was observed to be complete via ^{1}H-NMR monitoring due to complete consumption of the dibromo starting material. The reaction was cooled to room temperature and transferred to a separatory funnel containing water and DCM, shaken vigorously, and the organic layer collected. The aqueous layer was washed three more times with DCM. All of the organic layers were combined, dried over Na_2SO_4, and concentrated to dryness to yield the crude material. The crude was subjected to silica gel chromatography using 100% hexanes and increasing to 20% EtOAc/hexanes where the product was observed to elute as a purple-colored solution and yielded a green solid when concentrated (171 mg, 0.124 mmol, 22%). ^{1}H-NMR (300 MHz; C_6D_6): δ 8.15 (d, *J* = 1.1 Hz, 2H), 7.94 (d, *J* = 7.3 Hz, 2H), 7.57-7.50 (ms, 10H), 7.44 (dd, *J* = 8.0, 1.3 Hz, 4H), 7.27 (d, *J* = 0.6 Hz, 2H), 7.09-7.01 (ms, 6H), 6.96 (dd, *J* = 7.7, 1.3 Hz, 2H), 6.86 (dd, *J* = 7.5, 1.9 Hz, 2H), 6.70 (d, *J* = 8.9 Hz, 4H), 6.65 (d, *J* = 8.9 Hz, 4H), 3.07 (q, *J* = 7.7 Hz, 16H), 1.52-1.40 (m, 16H), 1.27-1.08 (m, 16H), 0.87 (t, *J* = 7.3 Hz, 12H), 0.80 (t, *J* = 7.3 Hz, 12H). ^{13}C-NMR (75 MHz; C_6D_6): δ 191.7, 147.9, 147.0, 144.9, 138.2, 136.0, 133.5, 132.8, 131.8, 131.8, 131.7, 131.6, 128.9, 128.9, 127.4, 127.18, 127.03, 126.7, 124.8, 122.8, 122.5, 121.9, 121.5, 116.0, 113.7, 112.9, 112.5, 111.2, 51.1, 29.9, 29.9, 20.7, 14.2, 14.2. HRMS *m/z* calculated for $C_{97}H_{110}N_6O$ $[M]^+$ 1375.8770, found 1375.8759. IR (cm^{-1}) 3079, 3051, 2955, 2929, 2870, 1704, 1608, 1524, 1512, 1491, 1465, 1423, 1396, 1367, 1293, 1250, 1220, 1198, 1150, 1111, 1051.

9-(2,6-dimethylphenyl)-3,6-bis(1-methyl-2-phenylindolizin-3-yl)-9H-fluoren-9-ylium hexafluorophosphate (**^{2Ph}FluIndz**): To a flame dried round bottom flask under N_2 was added THF (3.6 mL) followed by **6** (0.085 g, 0.144 mmol). 2,6-dimethylphenylmagnesium bromide (0.72 mL, 1.0 M in THF, 0.72 mmol) was then added dropwise and the reaction allowed to stir at room temperature overnight. The next morning the reaction was observed to be complete via ^{1}H-NMR monitoring and was quenched with H_2O. The reaction was then extracted with DCM three times and the organic layer passed through a thin pad of silica gel, rinsing with DCM, and concentrated to yield the intermediate as an off red solid. The solid was added to a flame dried round bottom flask under N_2 followed by dichloroethane (5.0 mL) and $POCl_3$ (0.04 mL, 0.432 mmol) and heated

to reflux for 1 hour. At this point the reaction was observed to be complete as visualized *via* TLC monitoring for consumption of the intermediate. The reaction was added dropwise to ~100 mL of Et_2O to precipitate the crude product. The crude product was filtered off, rinsed with Et_2O, and redissolved into a clean round bottom flask using DCM. Then 5 mL of a saturated KPF_6 solution in MeCN was added dropwise to swap the existing anion for a PF_6^- anion. The reaction was allowed to stir until the salt swap was complete by TLC (all the product moving off the baseline in 10% MeCN/DCM), approximately 1 hour. At this point, the solids were filtered off, rinsed with DCM, and the filtrate concentrated to yield the crude product. The crude was subjected to silica gel chromatography beginning with 100% DCM and gradually increasing to 10% MeCN/DCM to elute the product as a dark purple colored solution and a sparkly dark purple solid when concentrated (0.075 g, 0.091 mmol, 63%). ^{1}H- and ^{13}C-NMRs were not obtained due to insufficient resolution (broad peaks, no discernable aromatic signals) in a multitude of NMR solvents attempted, likely due to the presence of thermally accessible diradicals. Anal. Calcd for 8:1 dye ($C_{51}H_{39}N_2PF_6$):CH_2Cl_2: C, 73.50; H, 4.74; N, 3.35. Found: C, 73.49; H, 4.83; N, 3.37. Residual DCM is included in the formula herein as it was observed to be present in a ^{1}H-NMR of the sample, despite being placed on vacuum overnight. HRMS *m/z* calculated for $C_{51}H_{39}N_2$ $[M - PF_6^-]^+$ 679.3108, found 679.3100. IR (cm^{-1}) 3058, 2921, 2857, 1617, 1588, 1545, 1521, 1505, 1471, 1433, 1388, 1369, 1320, 1282, 1225, 1208, 1153, 1134, 1096, 1036, 1001.

9-(2,6-dimethylphenyl)-3,6-bis(1-phenylindolizin-3-yl)-9H-fluoren-9-ylium hexafluorophosphate (^{1Ph}**FluIndz**): To a flame dried round bottom flask under N_2 was added THF (6.6 mL) followed by **7** (0.148 g, 0.264 mmol). 2,6-dimethylphenylmagnesium bromide (0.79 mL, 1.0 M in THF, 0.79 mmol) was then added dropwise and the reaction allowed to stir at room temperature overnight. The next morning the reaction was observed to be complete as visualized *via* ^{1}H-NMR monitoring and was quenched with H_2O. The reaction was then extracted with DCM three times and the organic layer passed through a thin pad of silica gel, rinsing with DCM, and concentrated to yield the intermediate as an off red solid. The solid was added to a flame dried round bottom flask under N_2 followed by dichloroethane (13.2 mL) and $POCl_3$ (0.074 mL, 0.792 mmol) and heated to reflux for 2 hours. At this point the reaction was observed to be complete *via* TLC monitoring for consumption of the intermediate. The reaction was added dropwise to ~100 mL of Et_2O to precipitate the crude product. The crude product was filtered off, rinsed with Et_2O, and redissolved into a clean round bottom flask using DCM. Then 5 mL of a saturated KPF_6 solution in MeCN was added dropwise to swap the existing anion for a PF_6^- anion. The reaction was allowed to stir until the salt swap was complete by TLC (all the product moving off the baseline in 10% MeCN/DCM), approximately 1 hour. At this point, the solids were filtered off, rinsed with DCM, and the filtrate concentrated to yield the crude product. The crude was subjected to silica gel chromatography beginning with 100% DCM and gradually increasing to 10% MeCN/DCM to elute the product as a dark brown colored solution and a sparkly dark brown solid when concentrated (0.069 g, 0.087 mmol, 32%). ^{1}H- and ^{13}C-NMRs were not obtained due to insufficient resolution (broad peaks, no discernable aromatic signals) in a multitude of NMR solvents attempted, likely due to the presence of thermally accessible diradicals. Anal. Calcd for $C_{49}H_{35}N_2PF_6$: C, 73.86; H, 4.43; N, 3.52. Found: C, 73.60; H, 4.45; N, 3.49. HRMS *m/z* calculated for $C_{49}H_{35}N_2$ $[M - PF_6^-]^+$ 651.2795, found 651.2805. IR (cm^{-1}) 3103, 3059, 2920, 2855, 1615, 1588, 1506, 1470, 1432, 1355, 1283, 1238, 1209, 1130, 1105, 1027, 1011.

3,6-bis(7-(4-(dimethylamino)phenyl)-1-(2-ethylhexyl)-2-phenylindolizin-3-yl)-9-(2,6-dimethylphenyl)-9H-fluoren-9-ylium hexafluorophosphate (^{7DMA}**FluIndz**): To a flame dried round bottom flask under N_2 was added THF (4.4 mL) followed by **8** (0.181 g, 0.177 mmol). 2,6-dimethylphenylmagnesium bromide (0.53 mL, 1.0 M in THF, 0.53 mmol) was then added dropwise and the reaction allowed to stir at room temperature overnight. The next morning the reaction was observed to be complete via ^{1}H-NMR monitoring and was quenched with H_2O. The reaction was then extracted with DCM three times and the organic layer passed through a thin pad of silica, rinsing with DCM, and concentrated to yield the intermediate as an off yellow solid. The solid was added to a flame dried round bottom flask under N_2 followed by dichloroethane (8.9 mL) and $POCl_3$ (0.049 mL, 0.53 mmol) and heated to reflux for 2 hours. At this point the reaction was observed to be complete *via* TLC monitoring for consumption of the intermediate. The reaction was added dropwise to ~100 mL of Et_2O to precipitate the crude product. The crude product was filtered off, rinsed with Et_2O, and redissolved into a clean round bottom flask using DCM. Then 5 mL of a saturated KPF_6 solution in MeCN was added dropwise to swap the existing anion for a PF_6^- anion. The reaction was allowed to stir until the salt swap was complete by TLC (all the product moving off the baseline in 10% MeCN/DCM), approximately 1 hour. At this point, the solids were filtered off, rinsed with DCM, and the filtrate concentrated to yield the crude product. The crude was subjected to silica gel chromatography beginning with 100% DCM and gradually increasing to 10% MeCN/DCM to elute the product as a dark brown colored solution and a sparkly dark brown solid when concentrated (0.127 g, 0.101 mmol, 57%). ^{1}H- and ^{13}C-NMRs were not obtained due to insufficient resolution (broad peaks, no discernable aromatic signals) in a multitude of NMR solvents attempted, likely due to the presence of thermally accessible diradicals. Anal. Calcd for $C_{81}H_{85}N_4PF_6$: C, 77.24; H, 6.80; N, 4.45. Found: C, 77.15; H, 6.85; N, 4.36. HRMS *m/z* calculated for $C_{81}H_{85}N_4$ $[M-PF_6^-]^+$ 1113.6769, found 1113.6782. IR (cm^{-1}) 3174, 3059, 2954, 2924, 2856, 2808, 1630, 1579, 1537, 1475, 1433, 1414, 1372, 1332, 1293, 1262, 1227, 1198, 1171, 1136, 1081, 1041, 1024, 1006.

3,6-bis(1,7-bis(4-(dibutylamino)phenyl)-2-phenylindolizin-3-yl)-9-(2,6-dimethylphenyl)-9H-fluoren-9-ylium hexafluorophosphate (^{1,7DBA}**FluIndz**): To a flame dried round bottom flask under N_2 was added THF (3.1 mL) followed by **9** (0.171 g, 0.124 mmol). 2,6-dimethylphenylmagnesium bromide (0.37 mL, 1.0 M in THF, 0.37 mmol) was then added dropwise and the reaction allowed to stir at room temperature for 4 hours. At this time the reaction was observed to be complete via ^{1}H-NMR monitoring and was quenched with H_2O. The reaction was then extracted with DCM three times and the organic layer passed through a thin pad of silica, rinsing with DCM, and concentrated to yield the intermediate as a green solid. The solid was added to a flame dried round bottom flask under N_2 followed by dichloroethane (4.3 mL) and $POCl_3$ (0.024 mL, 0.259 mmol) and heated to reflux for 2 hours. At this point the reaction was observed to be complete via TLC monitoring for consumption of the intermediate. The reaction was added dropwise to ~100 mL of 1:1 hexanes/Et_2O to precipitate the crude product. The crude product was filtered off, rinsed with Et_2O, and redissolved into a clean round bottom flask using DCM. Then 5 mL of a saturated KPF_6 solution in MeCN was added dropwise to swap the existing anion for a PF_6^- anion. The reaction was allowed to stir until the salt swap was complete by TLC (all the product moving off the baseline in 10% MeCN/DCM), approximately 1 hour. At this point, the solids were filtered off, rinsed with DCM, and the filtrate concentrated to yield the crude product. The crude was subjected to silica gel chromatography beginning with 100% DCM and gradually increasing to 10% MeCN/DCM to elute the product as a reddish-brown colored solution and a sparkly dark brown

solid when concentrated (72 mg, 0.047 mmol, 38%). ^{1}H- and ^{13}C-NMRs were not obtained due to insufficient resolution (broad peaks, no discernable aromatic signals) in a multitude of NMR solvents attempted, likely due to the presence of thermally accessible diradicals. Anal. Calcd for 7:1 dye ($C_{105}H_{119}N_6PF_6$):CH_2Cl_2: C, 77.84; H, 7.42; N, 5.18. Found: C, 77.87; H, 7.46; N, 5.14. Residual DCM is included in the formula herein as it was observed to be present in a ^{1}H NMR of the sample, despite being placed on vacuum overnight. HRMS *m/z* calculated for $C_{105}H_{119}N_6^+$ [M – PF_6^-]$^+$ 1464.9525, found 1464.9496. IR (cm^{-1}) 3186, 3053, 2954, 2929, 2870, 1576, 1534, 1510, 1460, 1403, 1365, 1347, 1314, 1286, 1259, 1192, 1128, 1108, 1054, 1003.

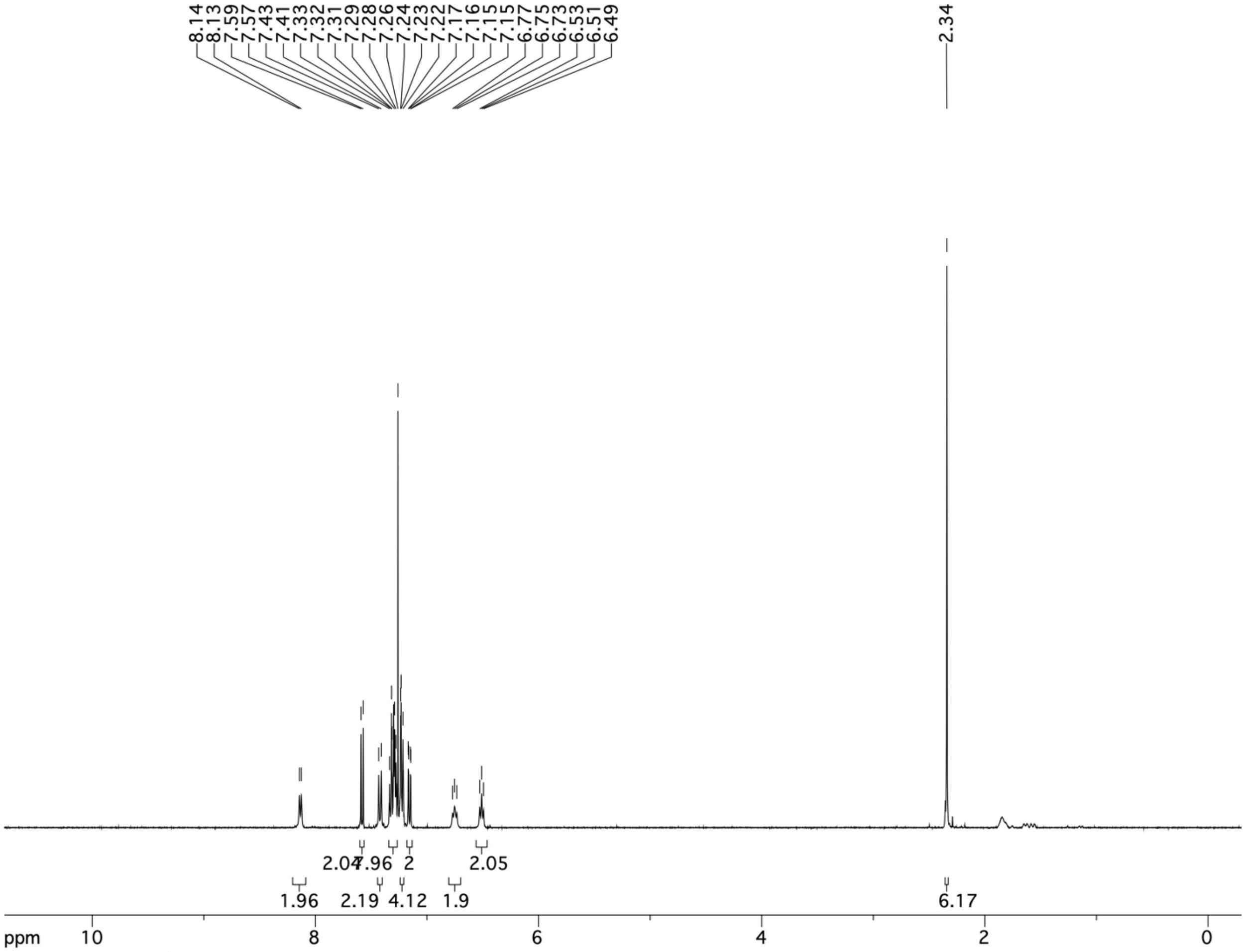

Figure S38. ^{1}H NMR (400 MHz) of **6** in $CDCl_3$.

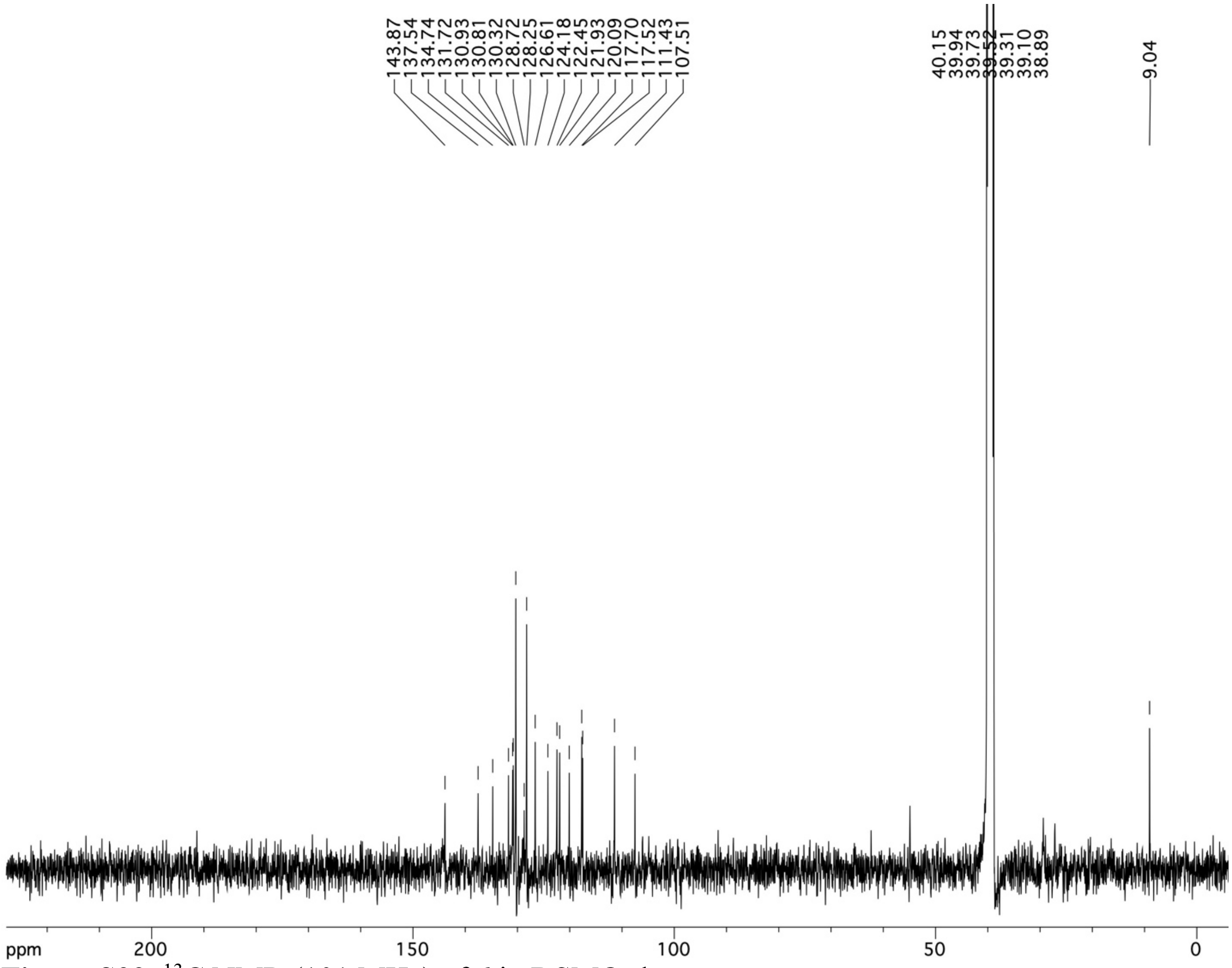

Figure S39. ^{13}C NMR (101 MHz) of **6** in DSMO-d$_6$.

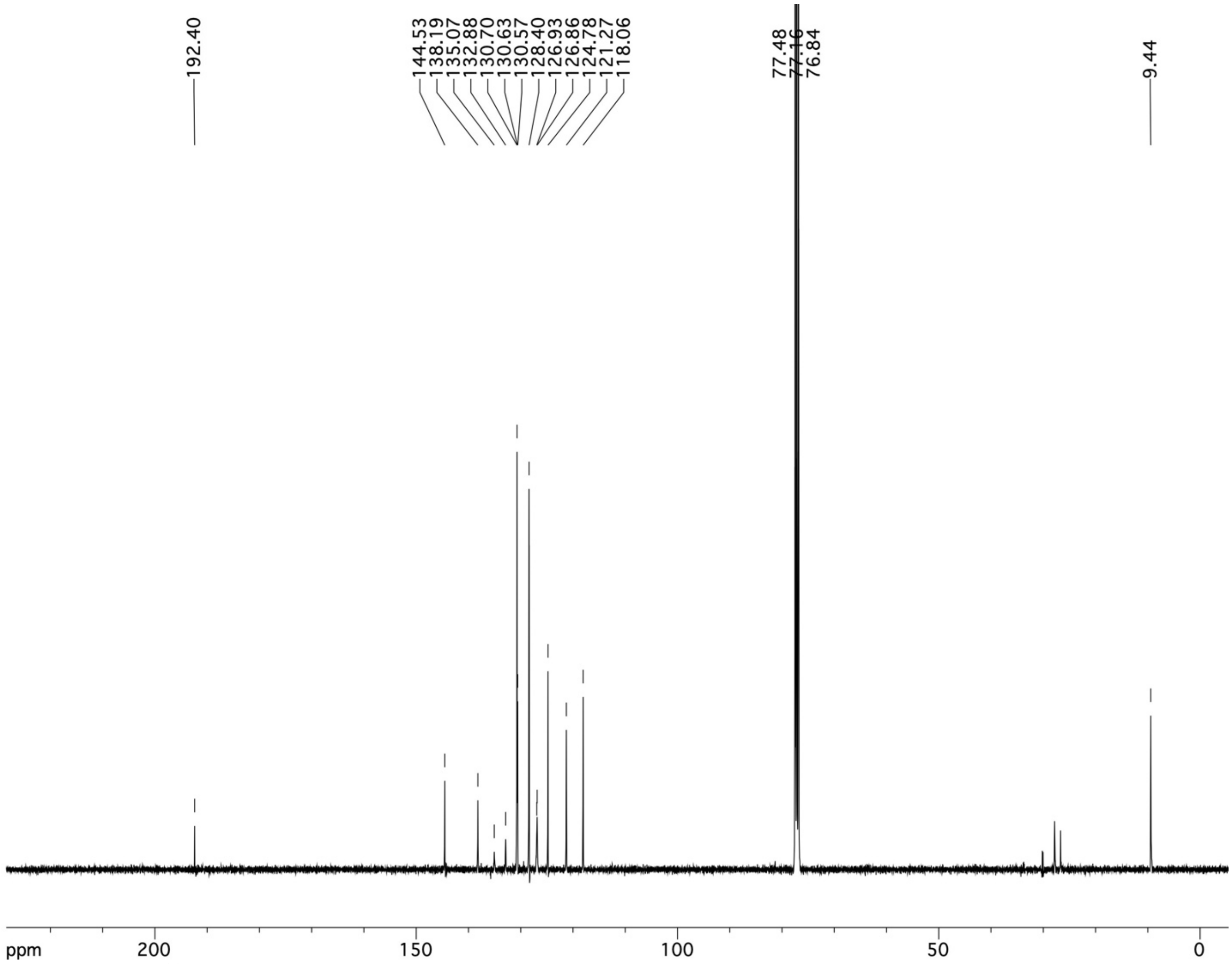

Figure S40. ^{13}C NMR (101 MHz) of **6** in $CDCl_3$.

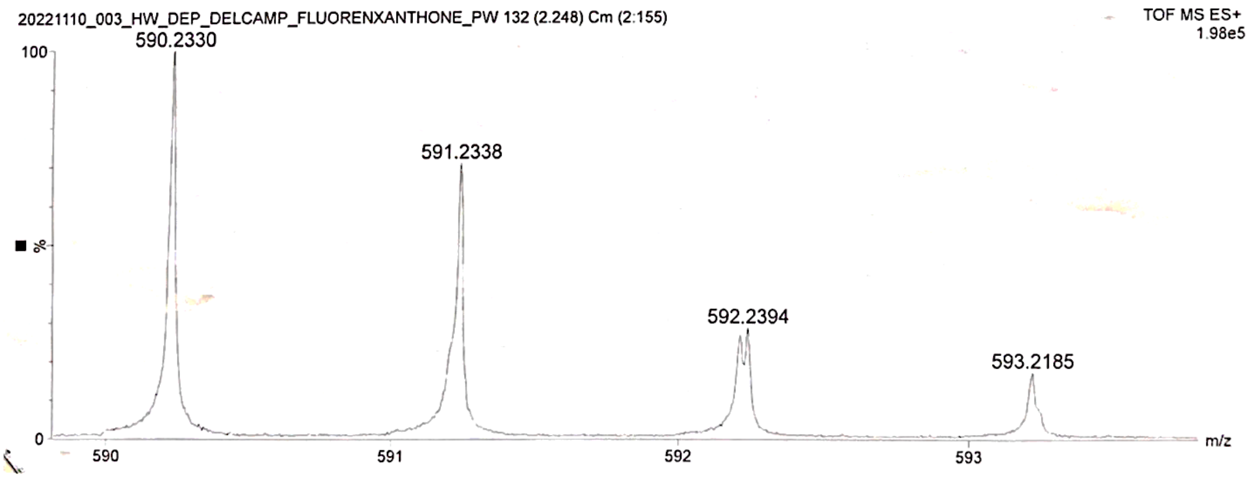

Figure S41. HRMS of **6**.

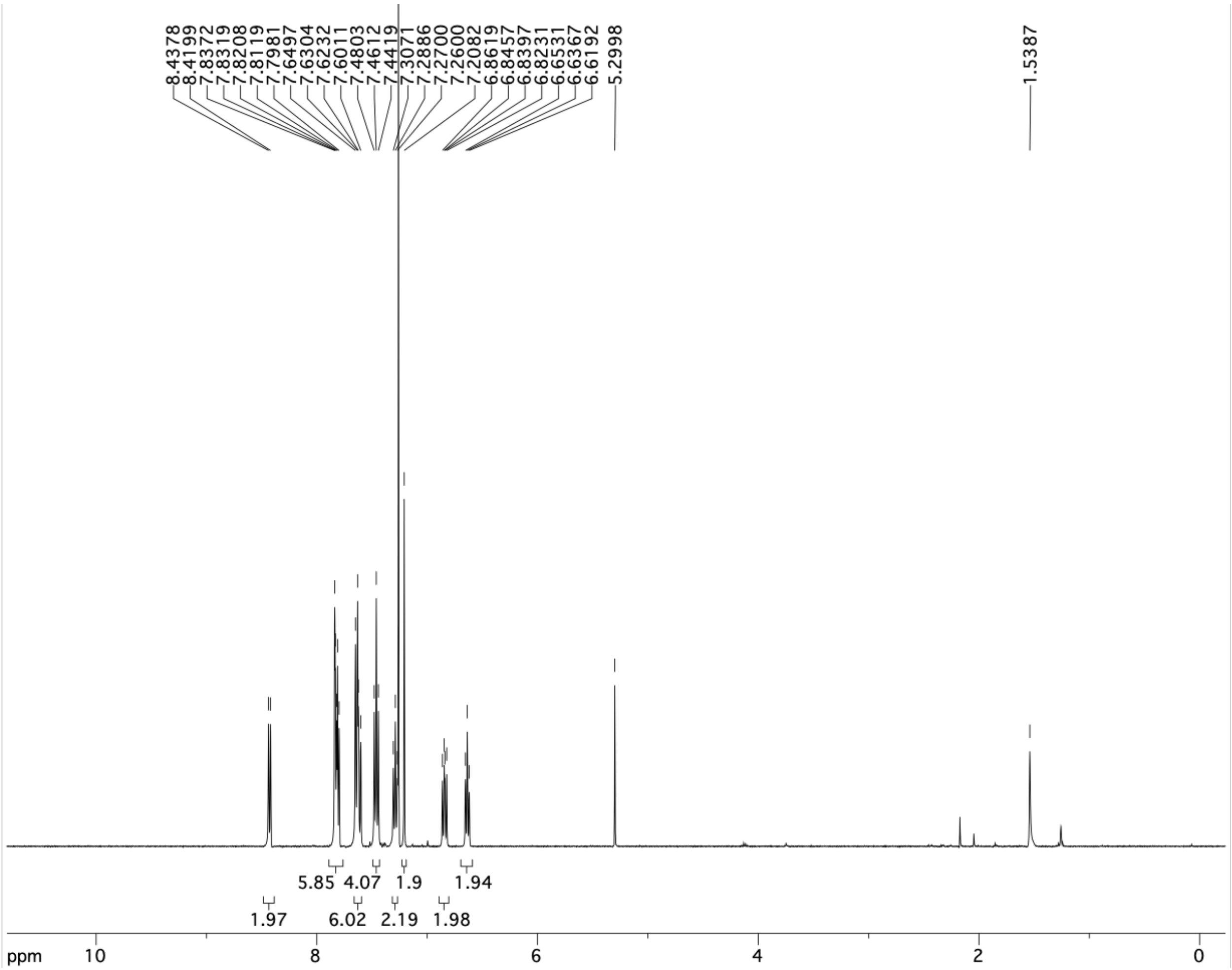

Figure S42. ^{1}H NMR (400 MHz) of **7** in $CDCl_3$.

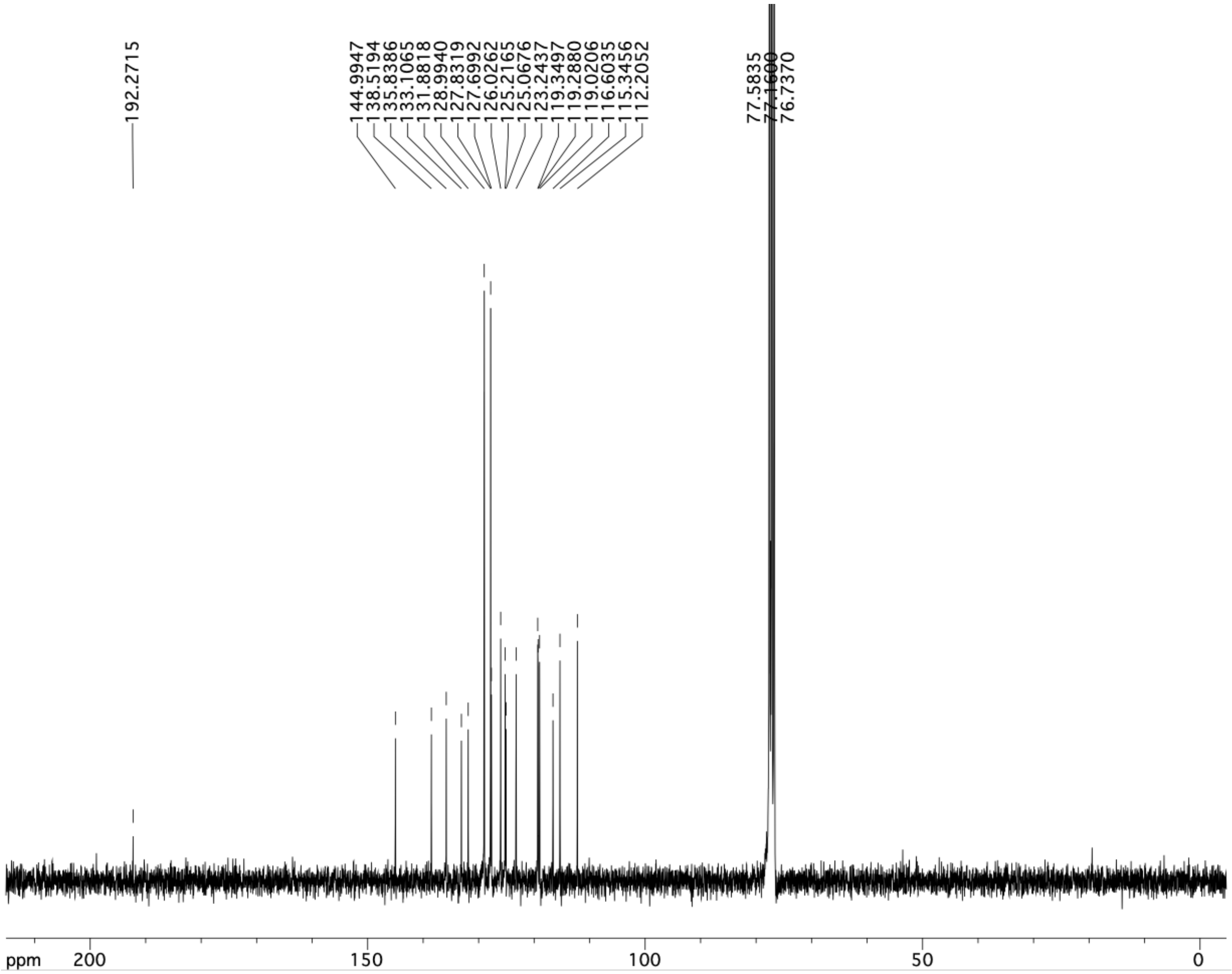

Figure S43. ^{13}C NMR (101 MHz) of **7** in $CDCl_3$.

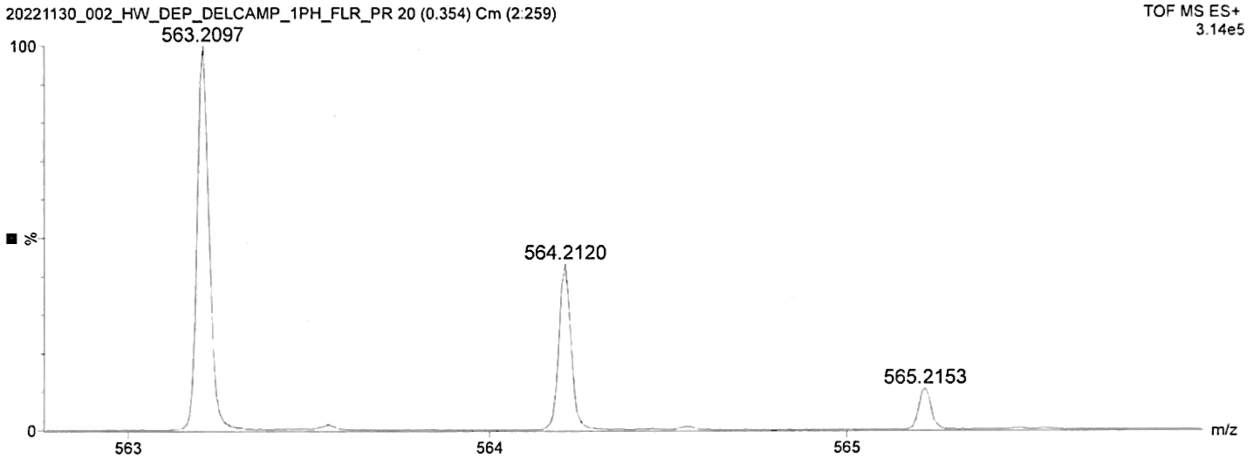

Figure S44. HRMS of **7**.

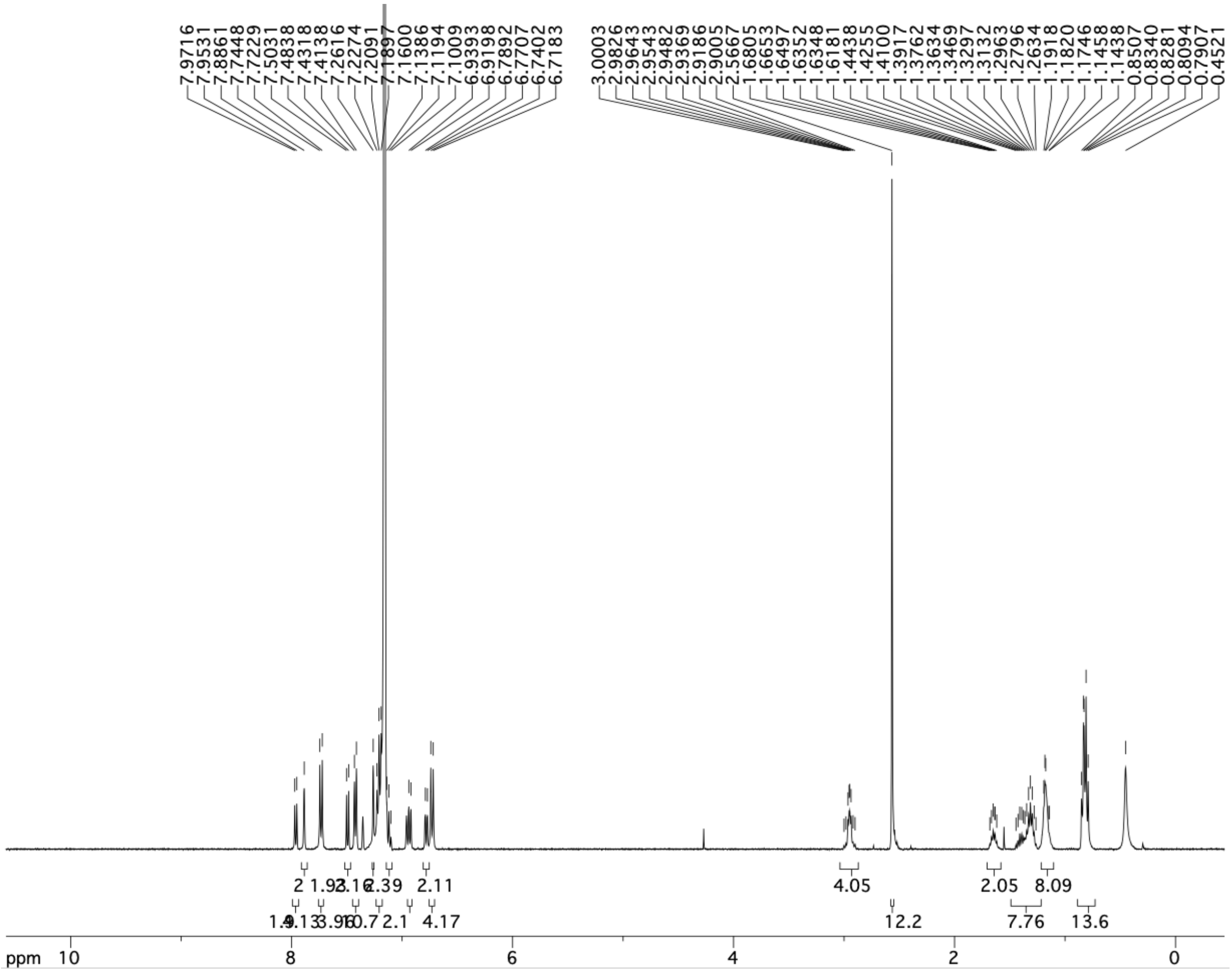

Figure S45. ^{1}H NMR (400 MHz) of **8** in C_6D_6.

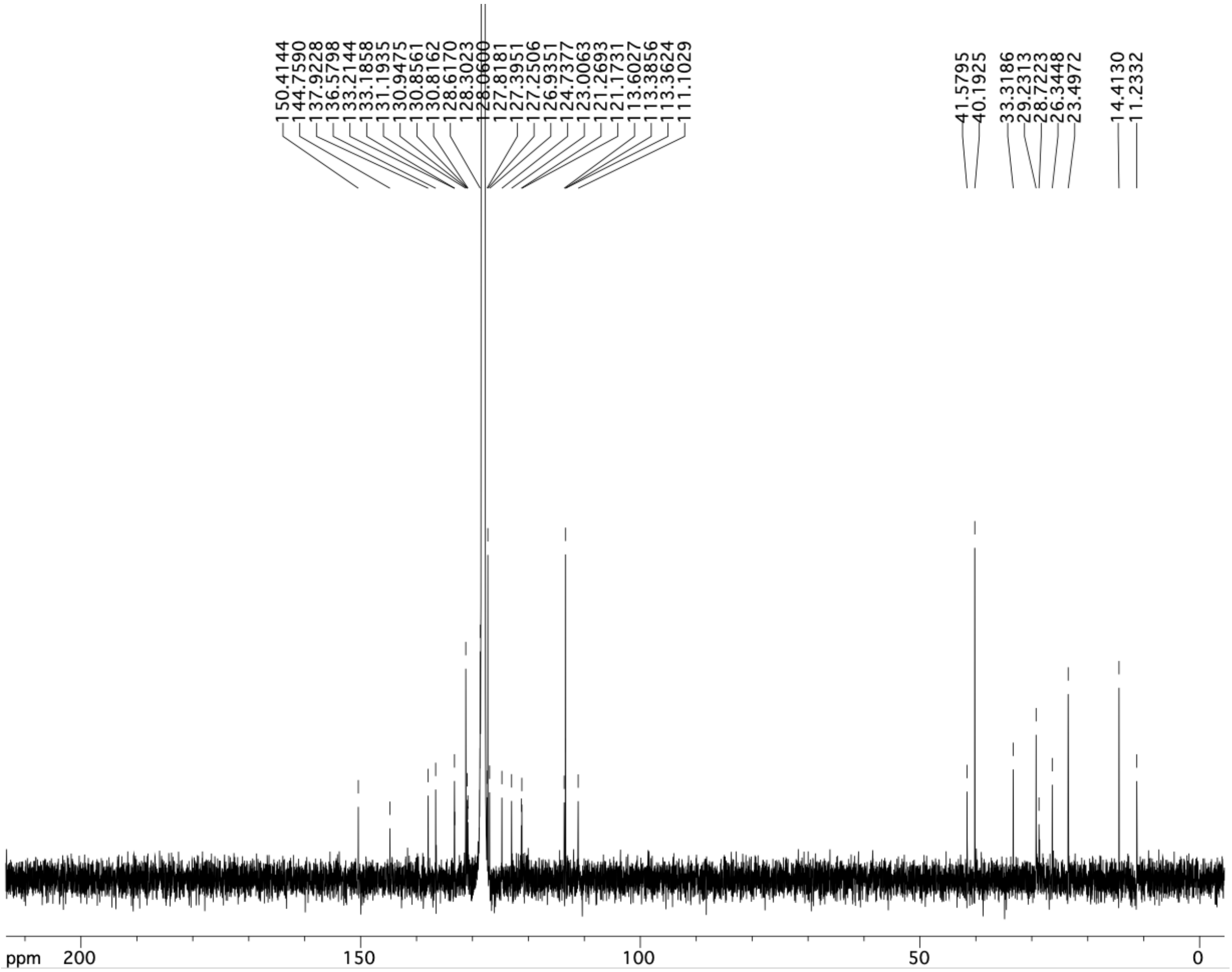

Figure S46. ^{13}C NMR (101 MHz) of **8** in C_6D_6.

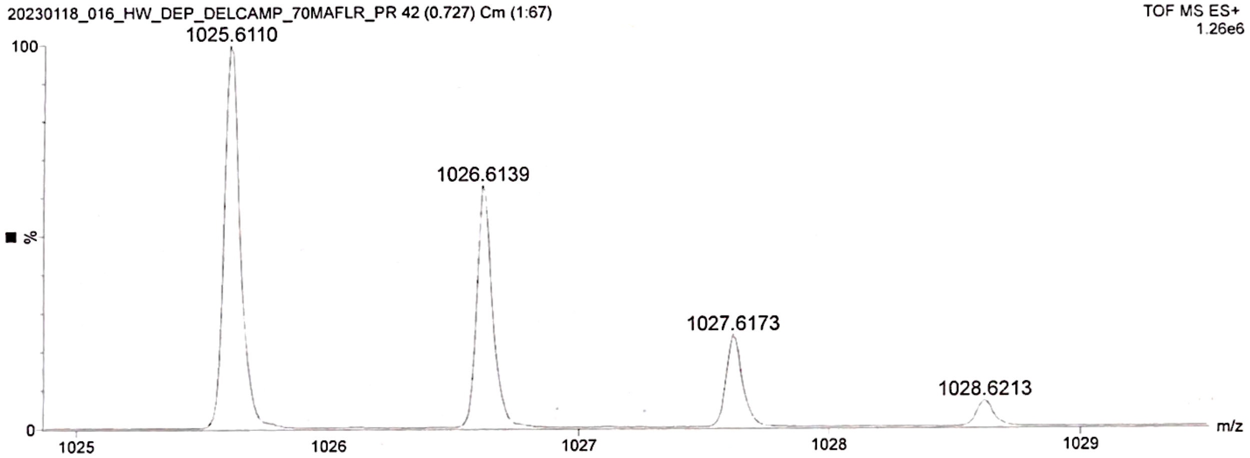

Figure S47. HRMS of **8**.

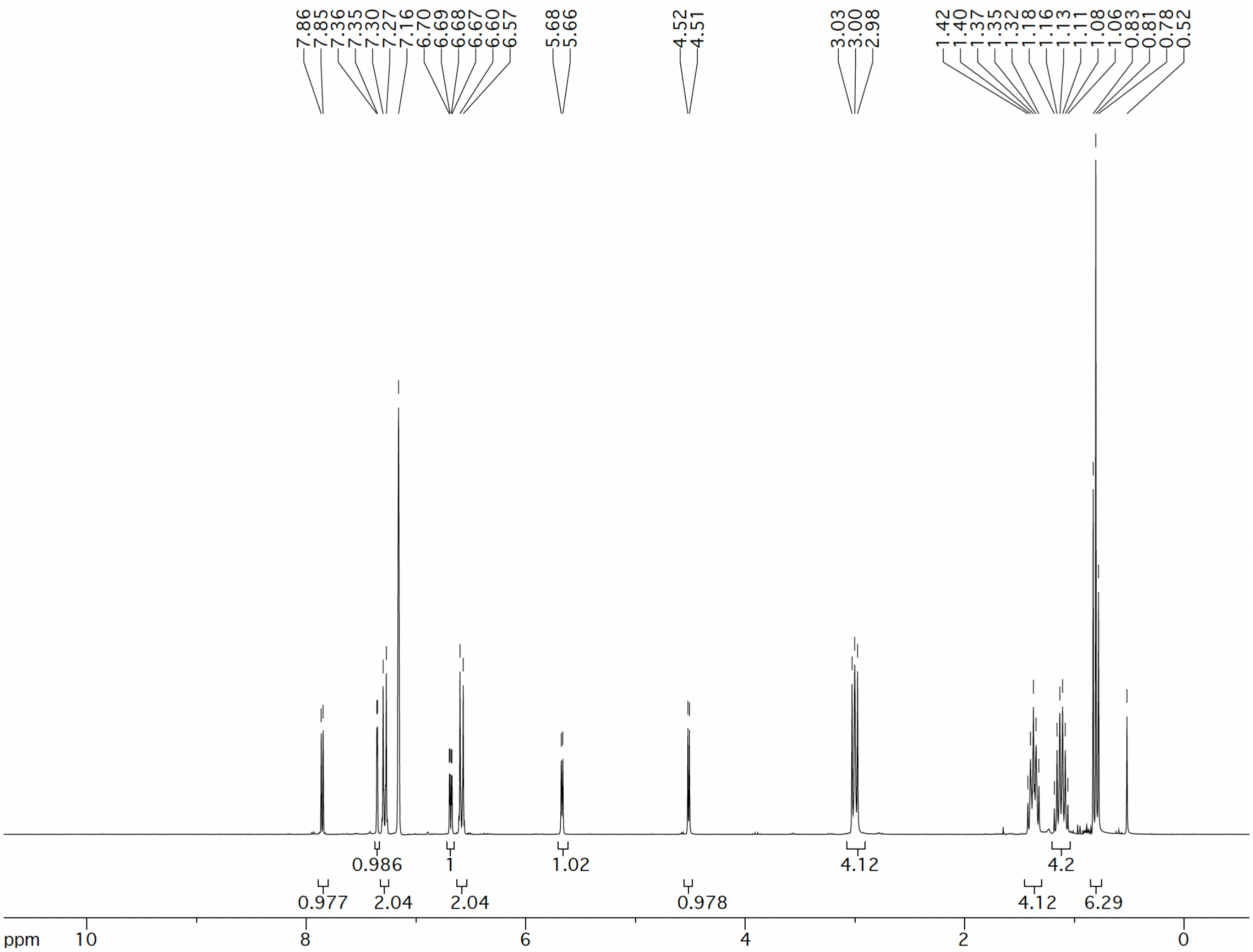

Figure S48. ^{1}H NMR (300 MHz) of **12** in C_6D_6.

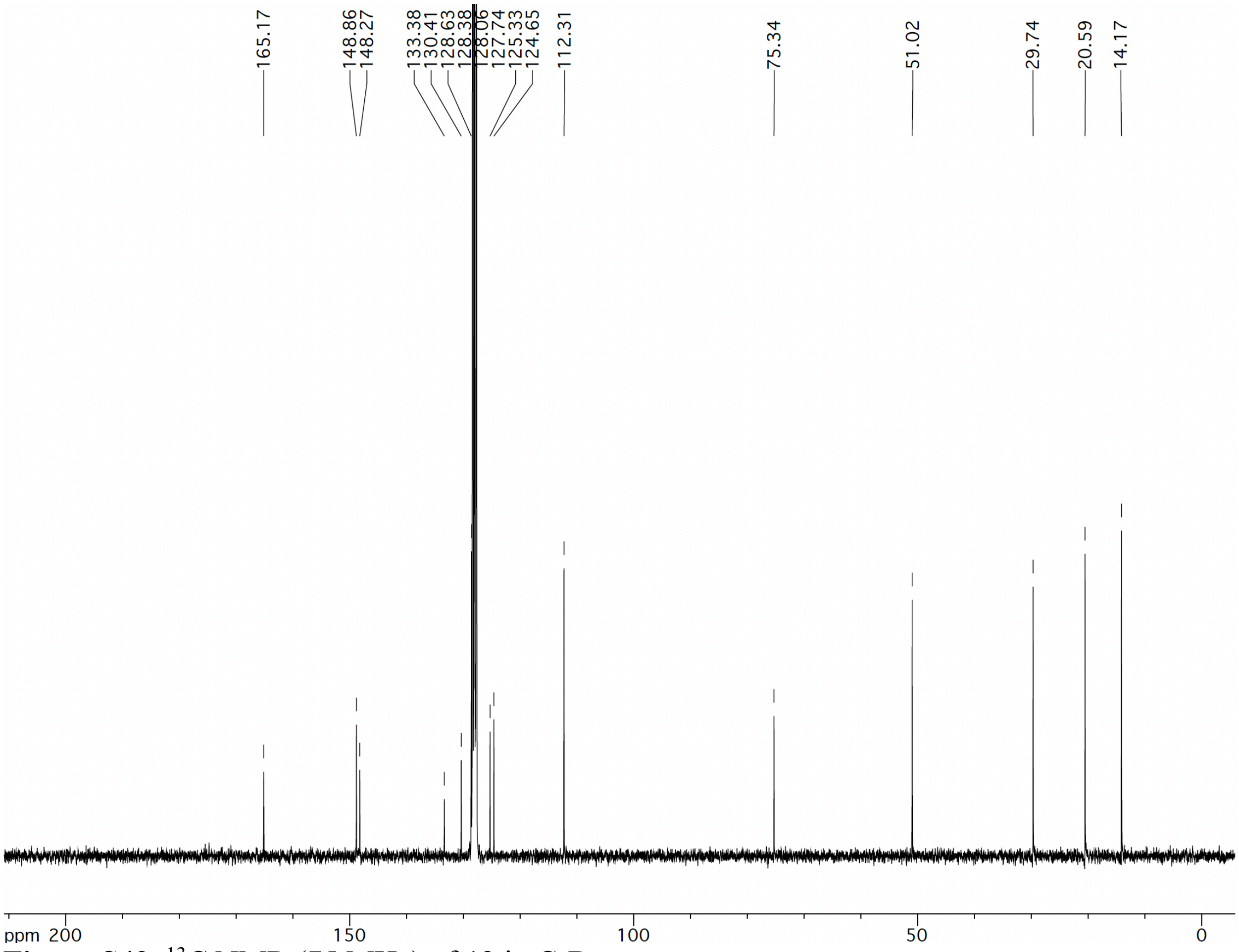

Figure S49. ^{13}C NMR (75 MHz) of **12** in C_6D_6.

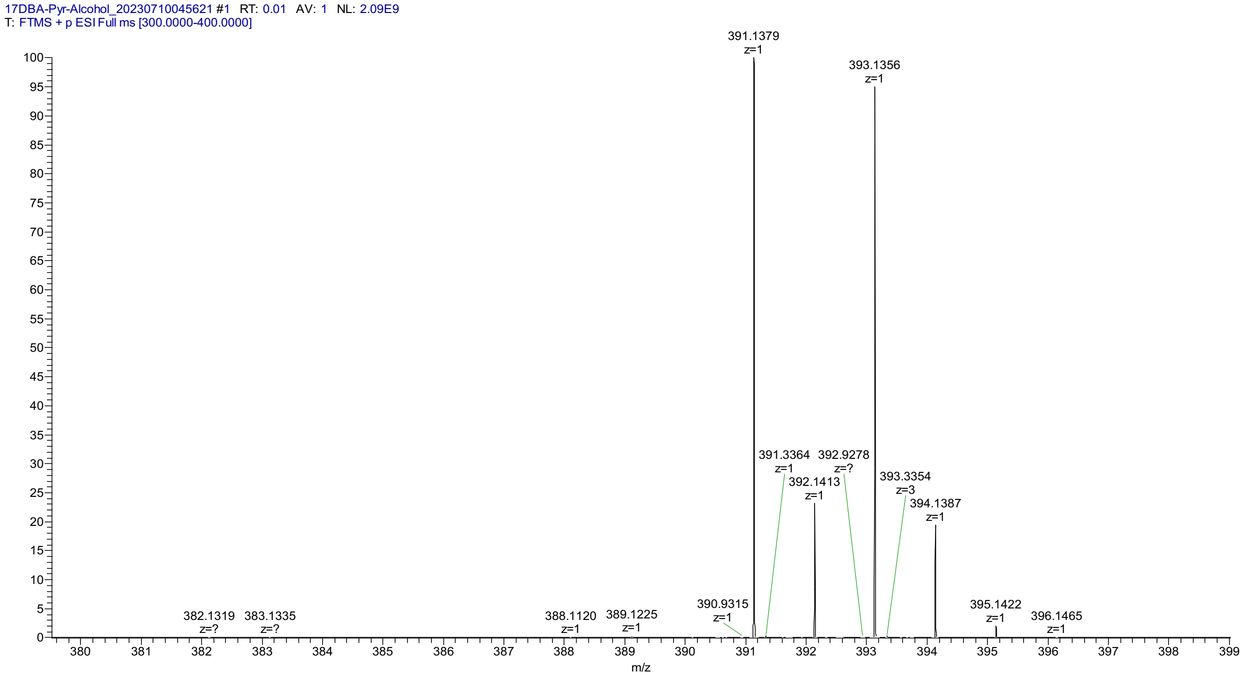

Figure S50. HRMS of **12**.

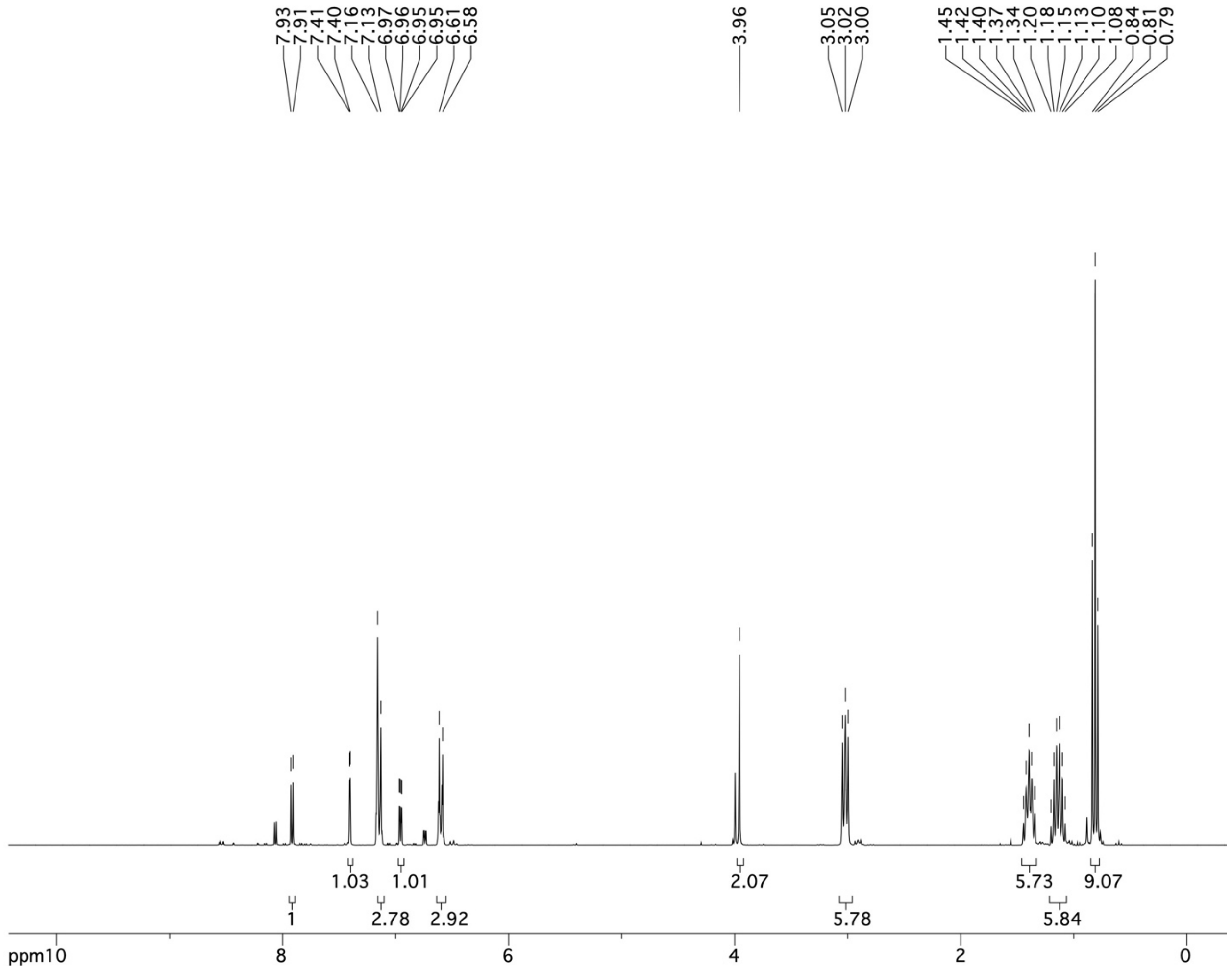

Figure S51. ^{1}H NMR (300 MHz) of **13** in C_6D_6.

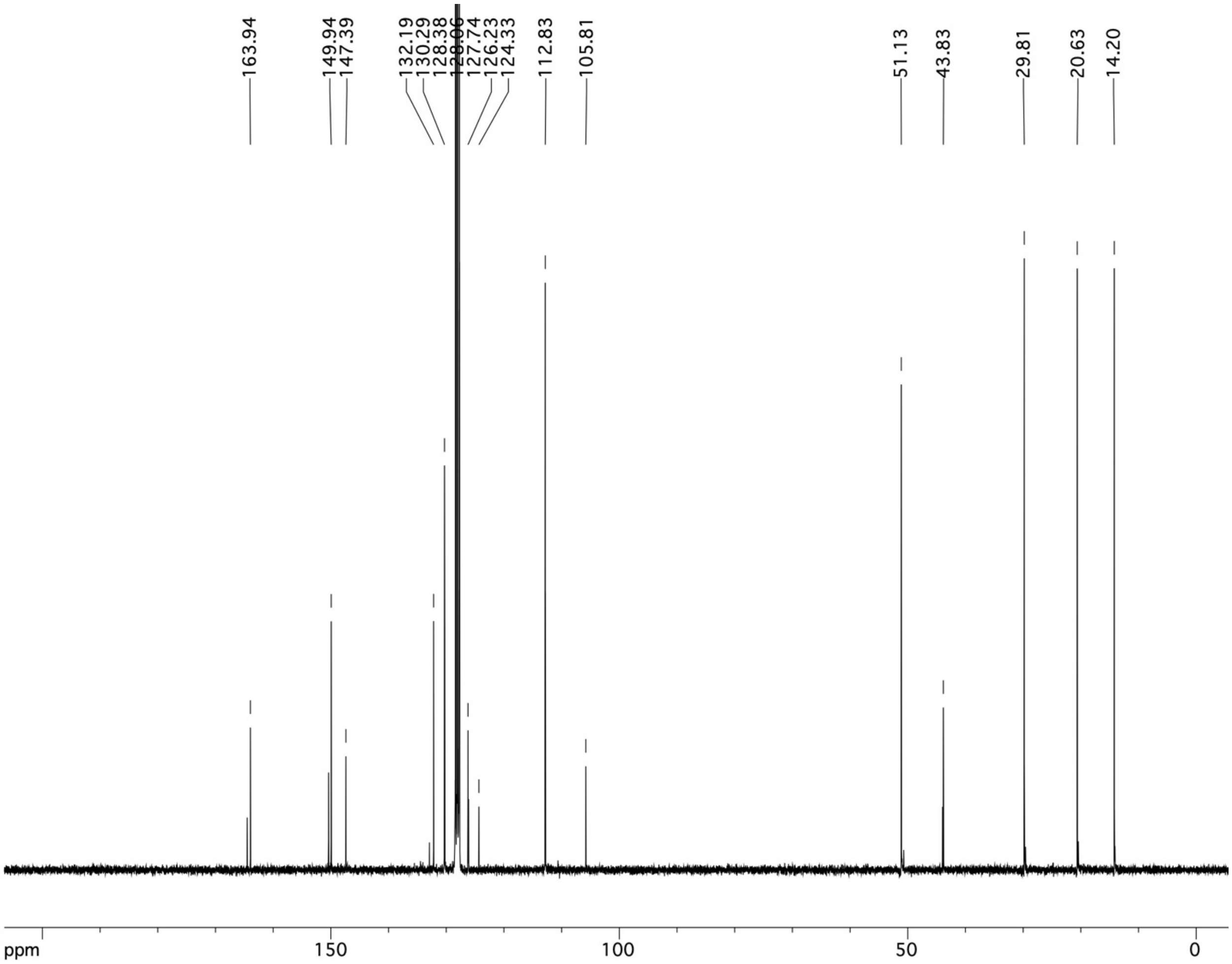

Figure S52. ^{13}C NMR (75 MHz) of **13** in C_6D_6.

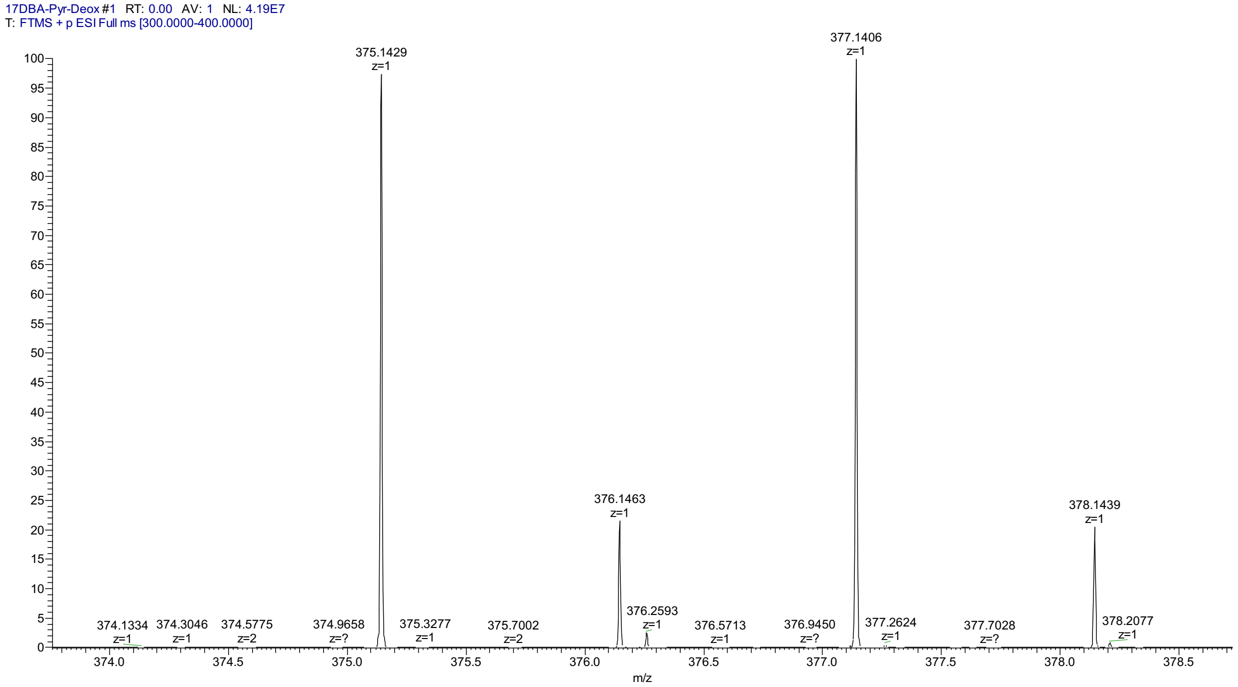

Figure S53. HRMS of **13**.

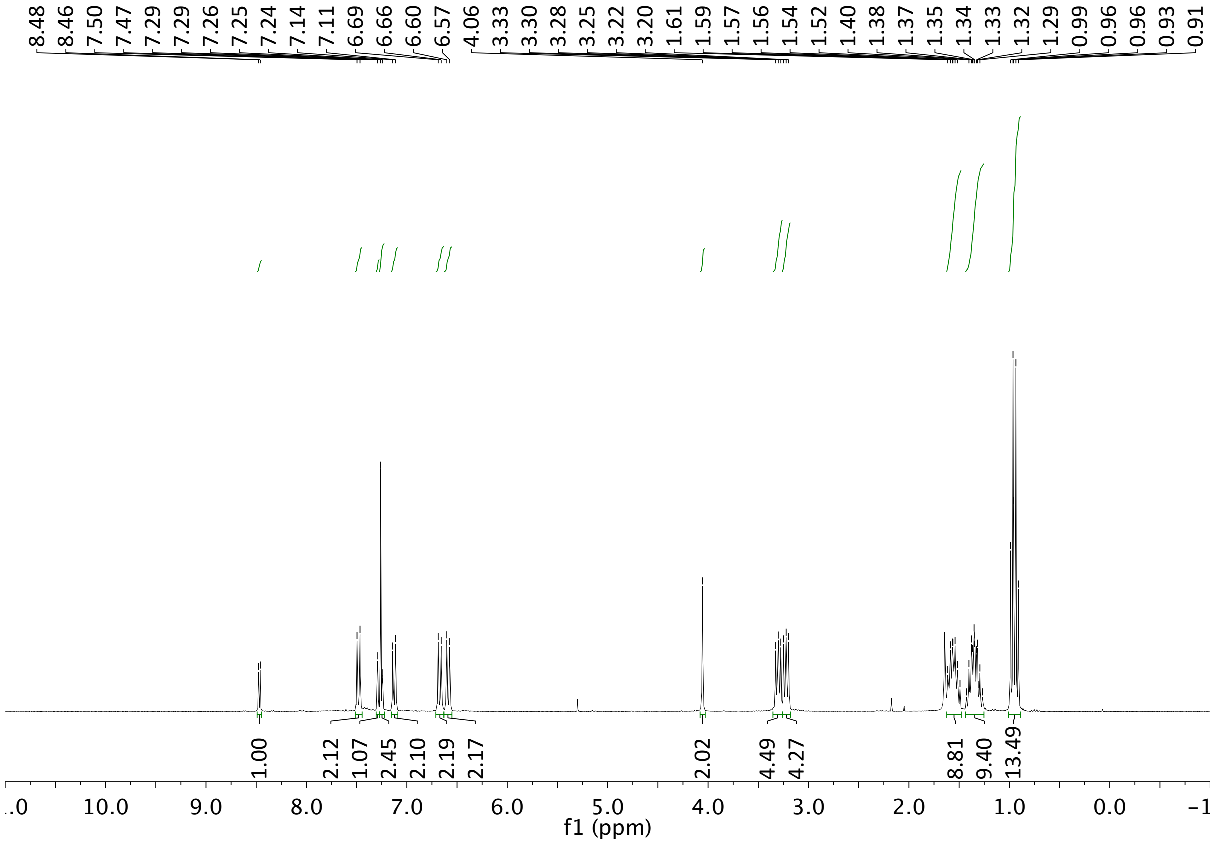

Figure S54. ^{1}H NMR (300 MHz) of **15** in $CDCl_3$.

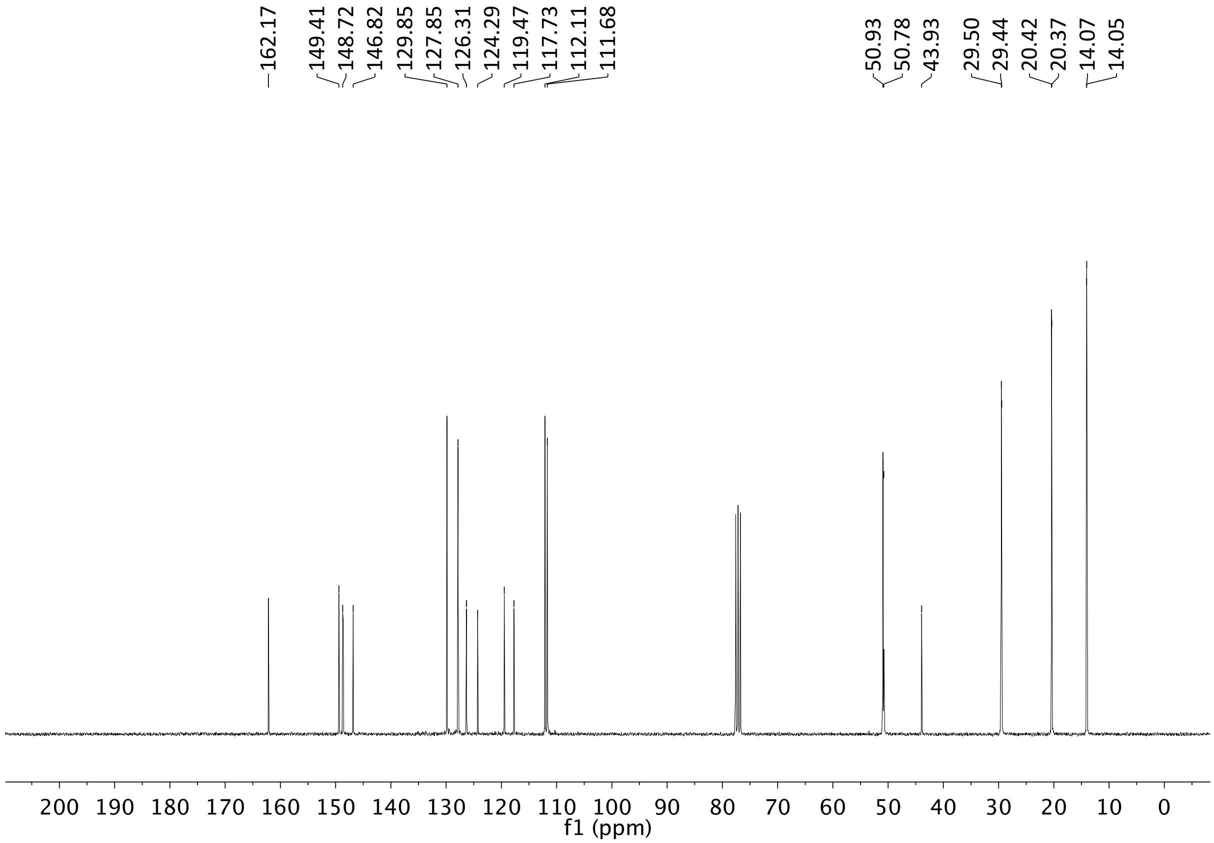

Figure S55. ^{13}C NMR (75 MHz) of **15** in $CDCl_3$.

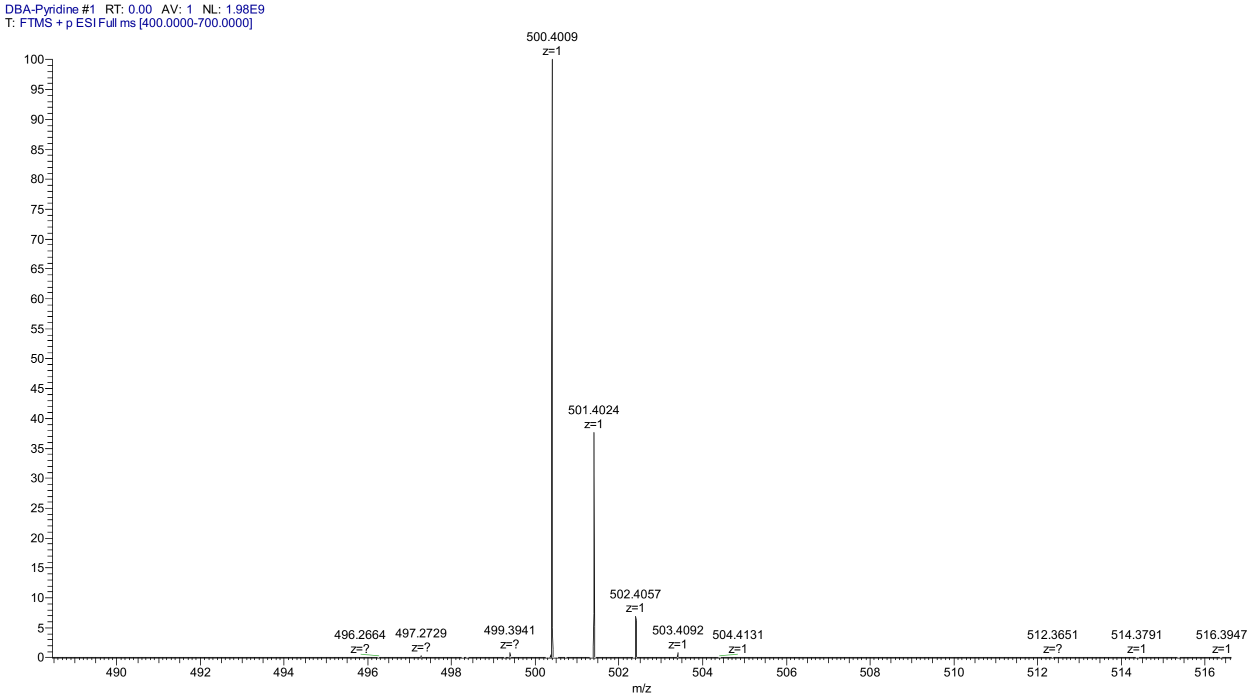

Figure S56. HRMS of **15**.

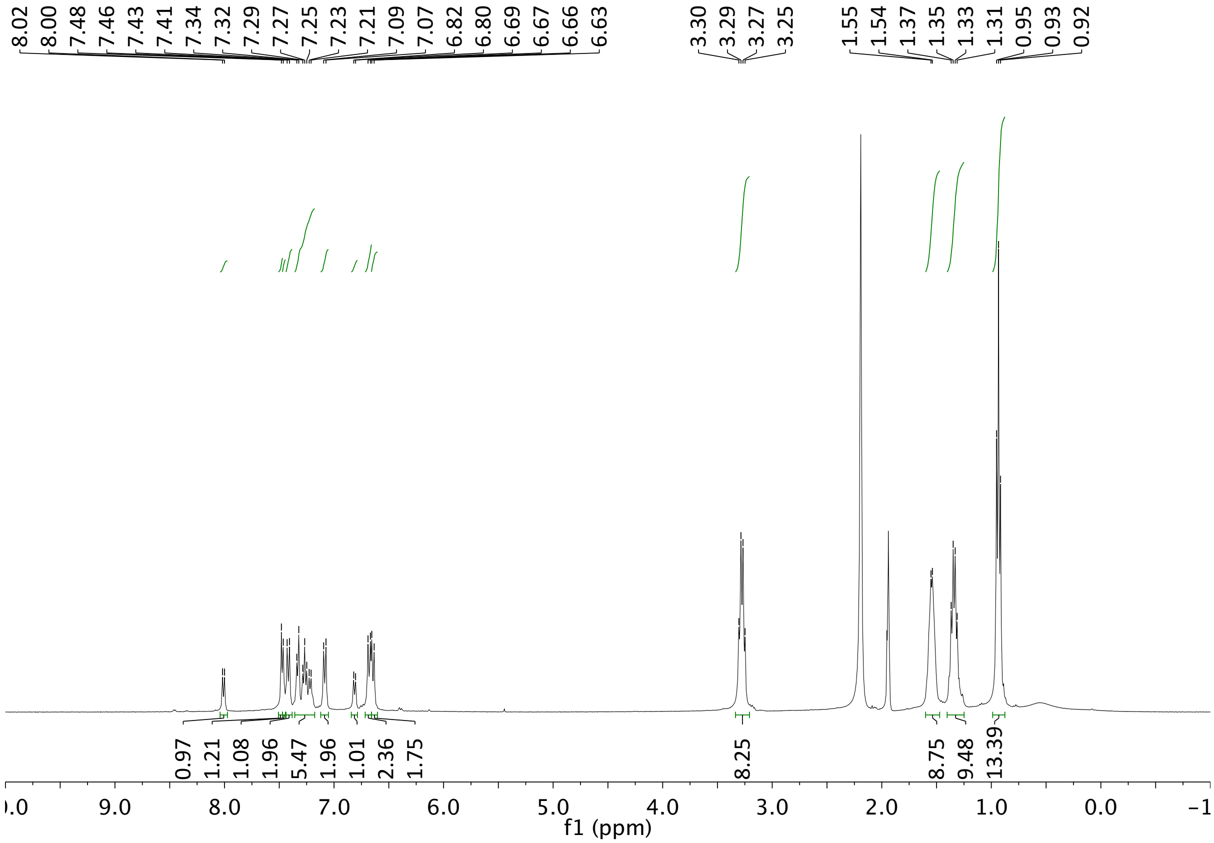

Figure S57. ^{1}H NMR (400 MHz) of **5** in CD_3CN.

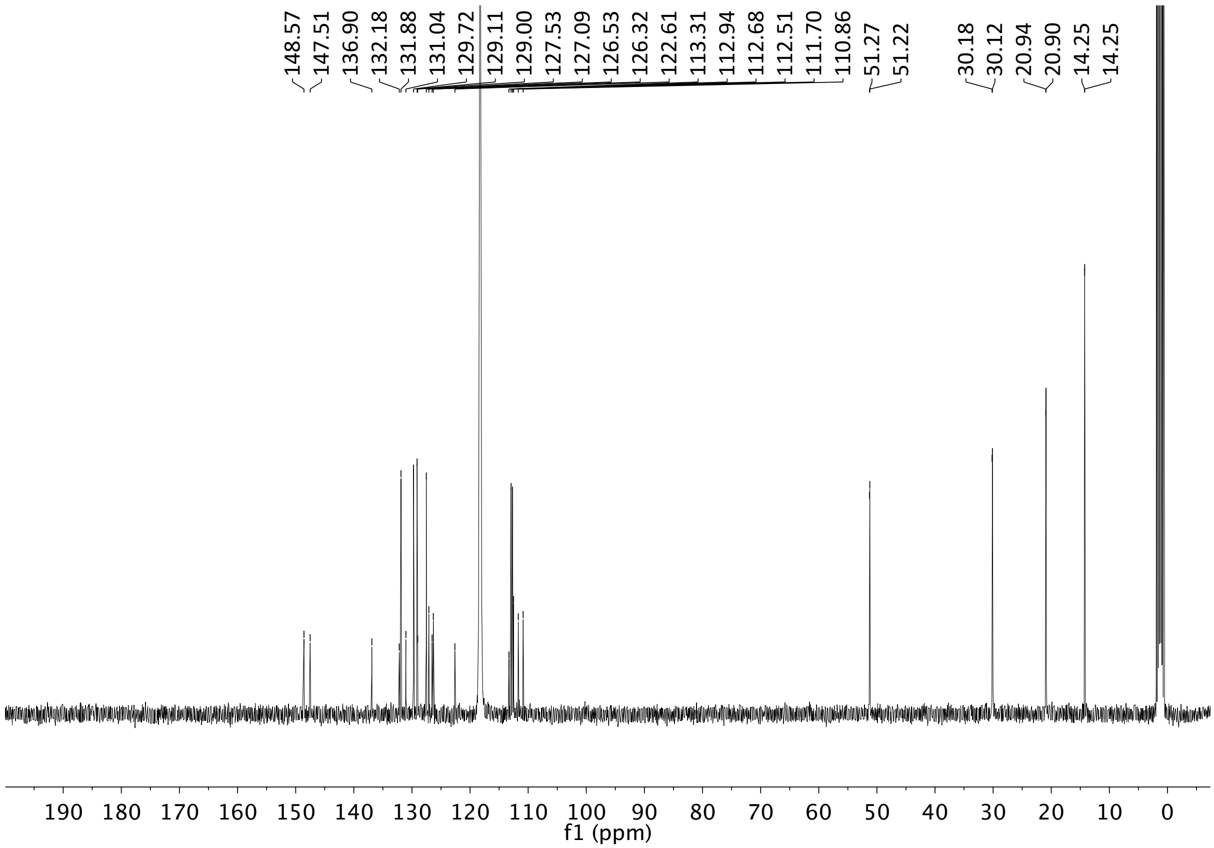

Figure S58. ^{13}C NMR (101 MHz) of **5** in CD_3CN.

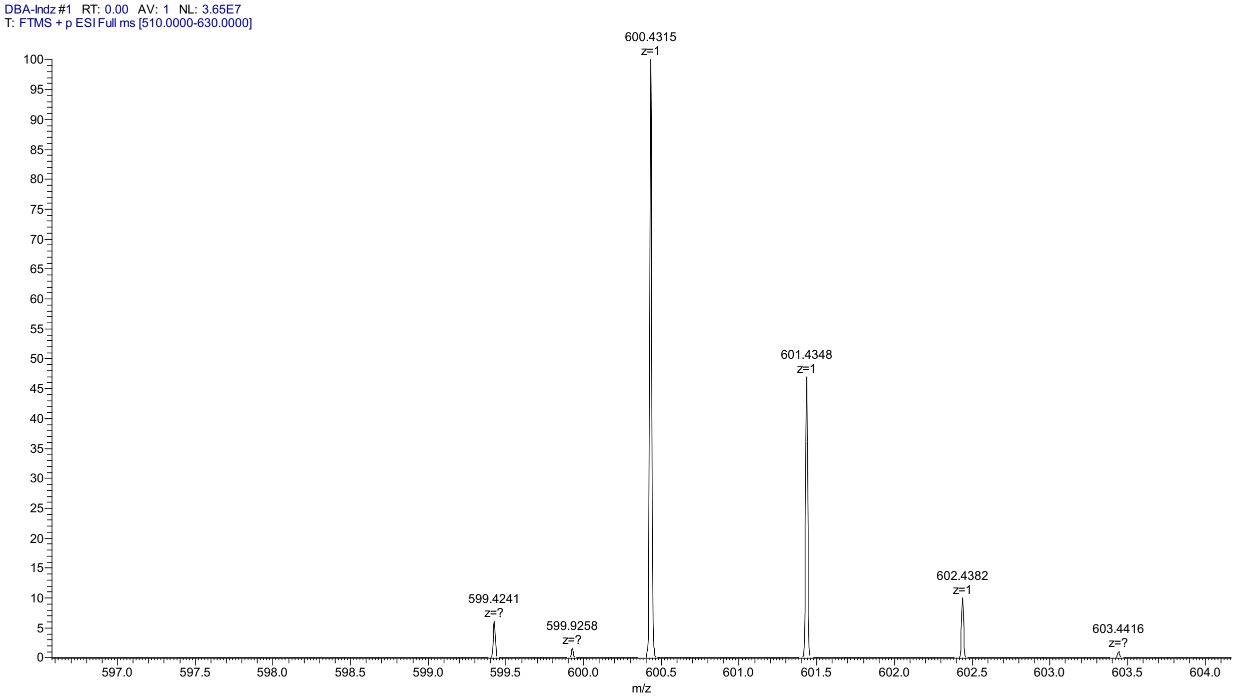

Figure S59. HRMS of **5**.

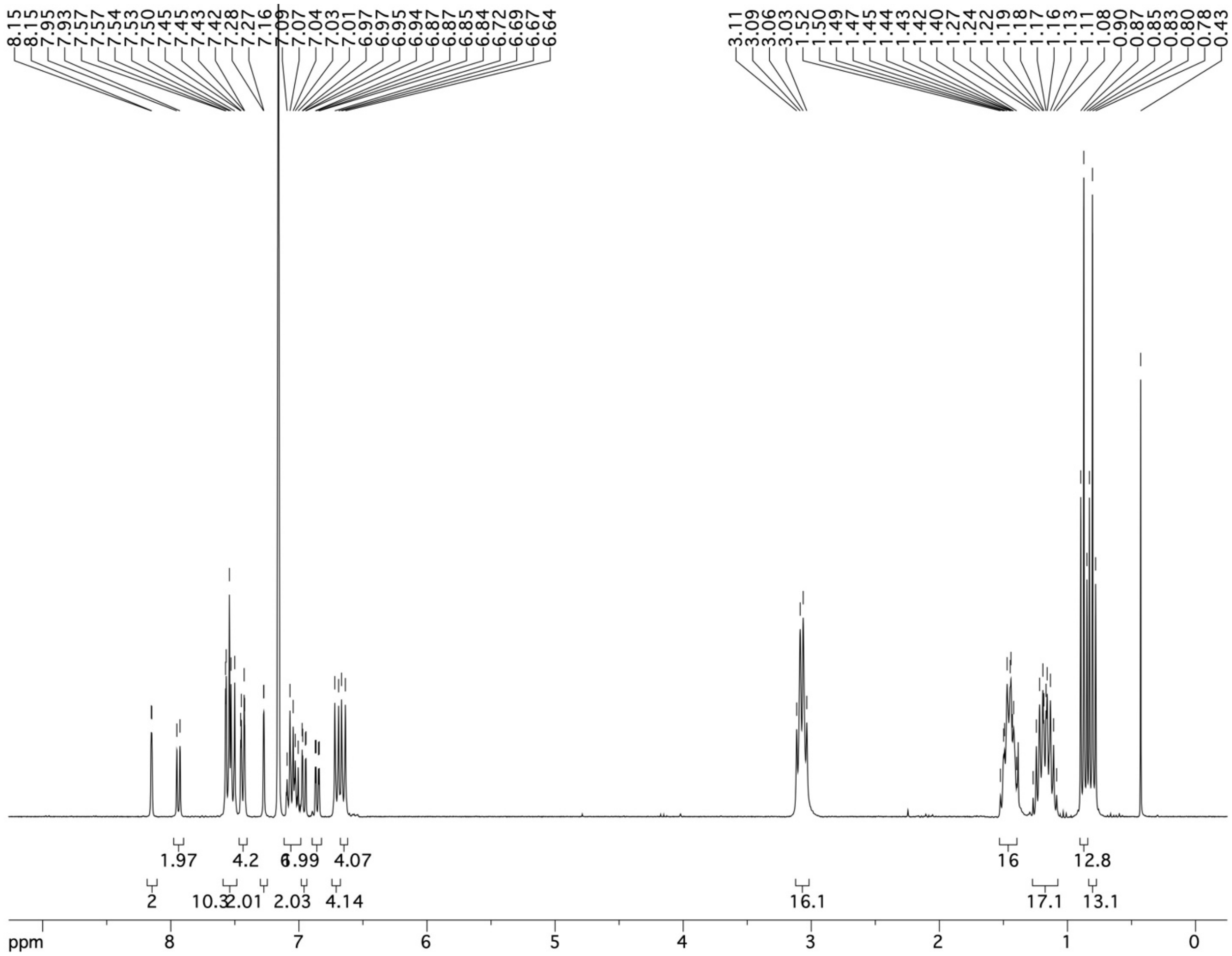

Figure S60. ^{1}H NMR (300 MHz) of **9** in C_6D_6.

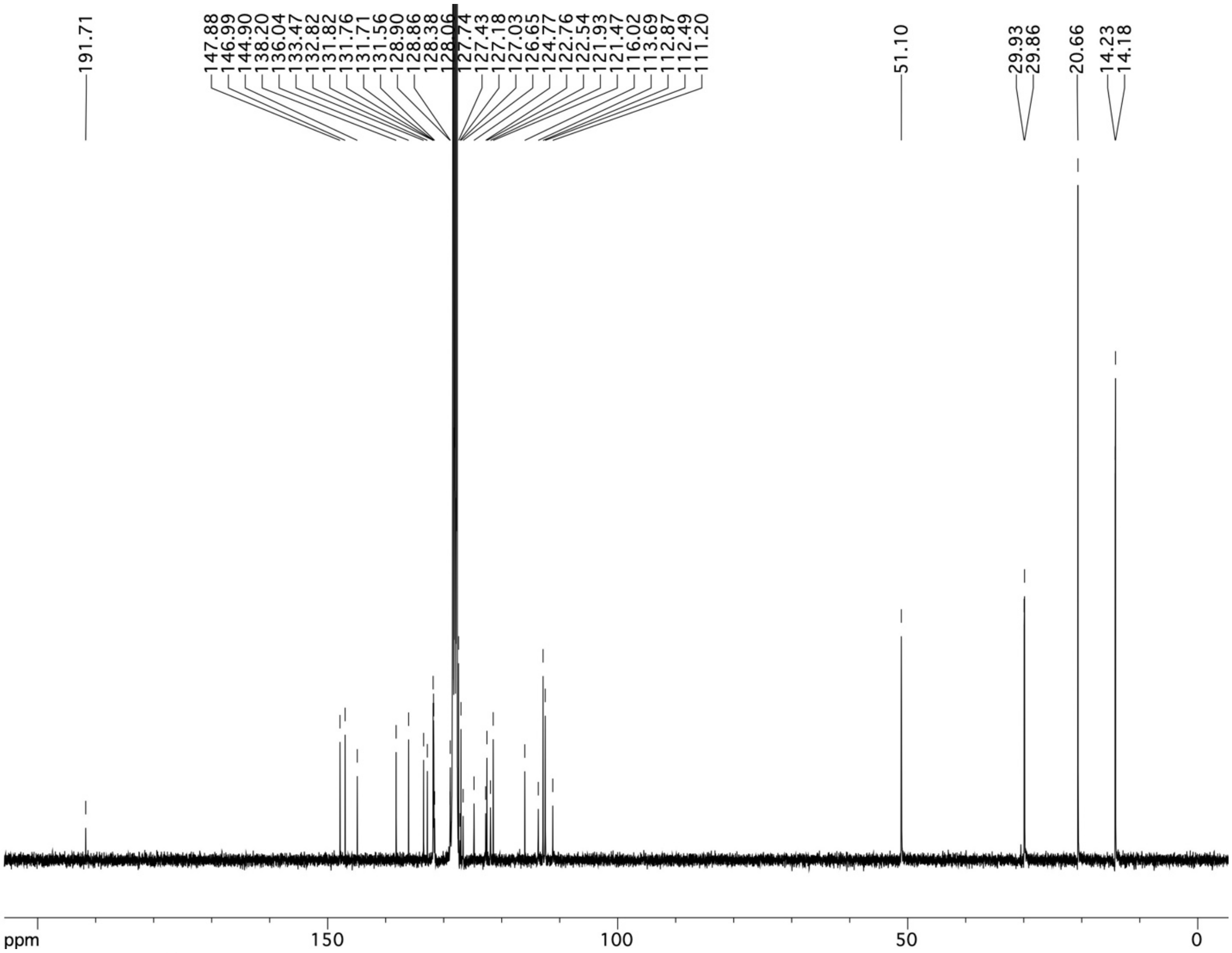

Figure S61. ^{13}C NMR (75 MHz) of **9** in C_6D_6.

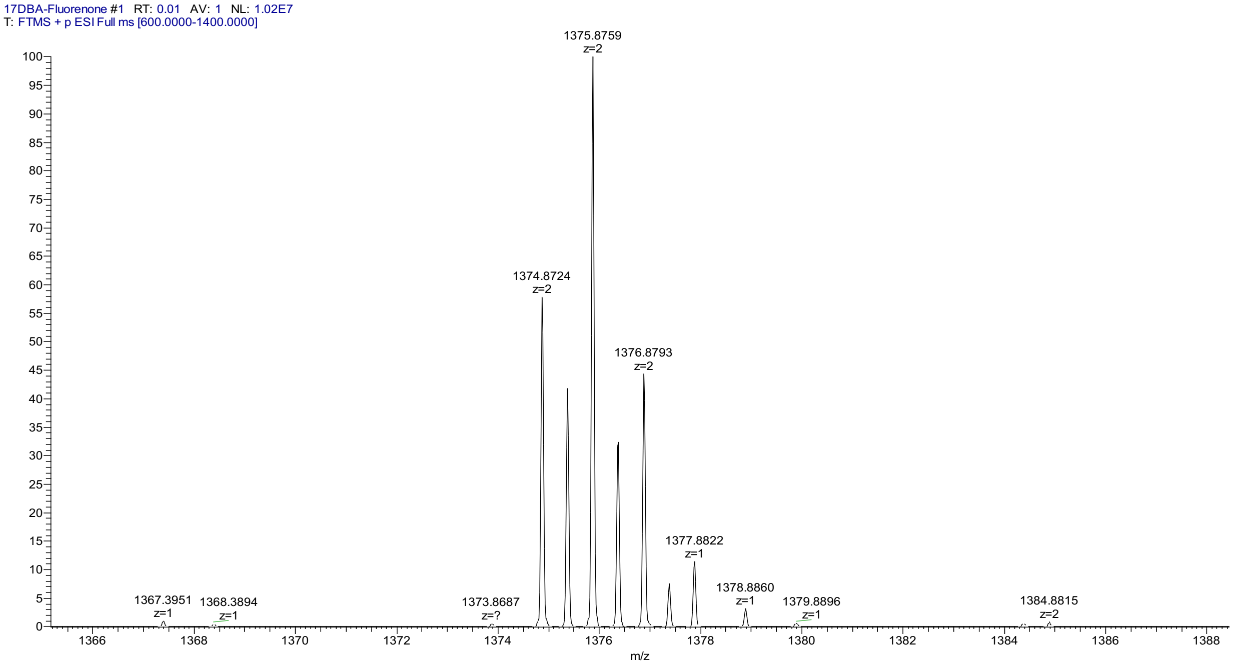

Figure S62. HRMS of **9**.

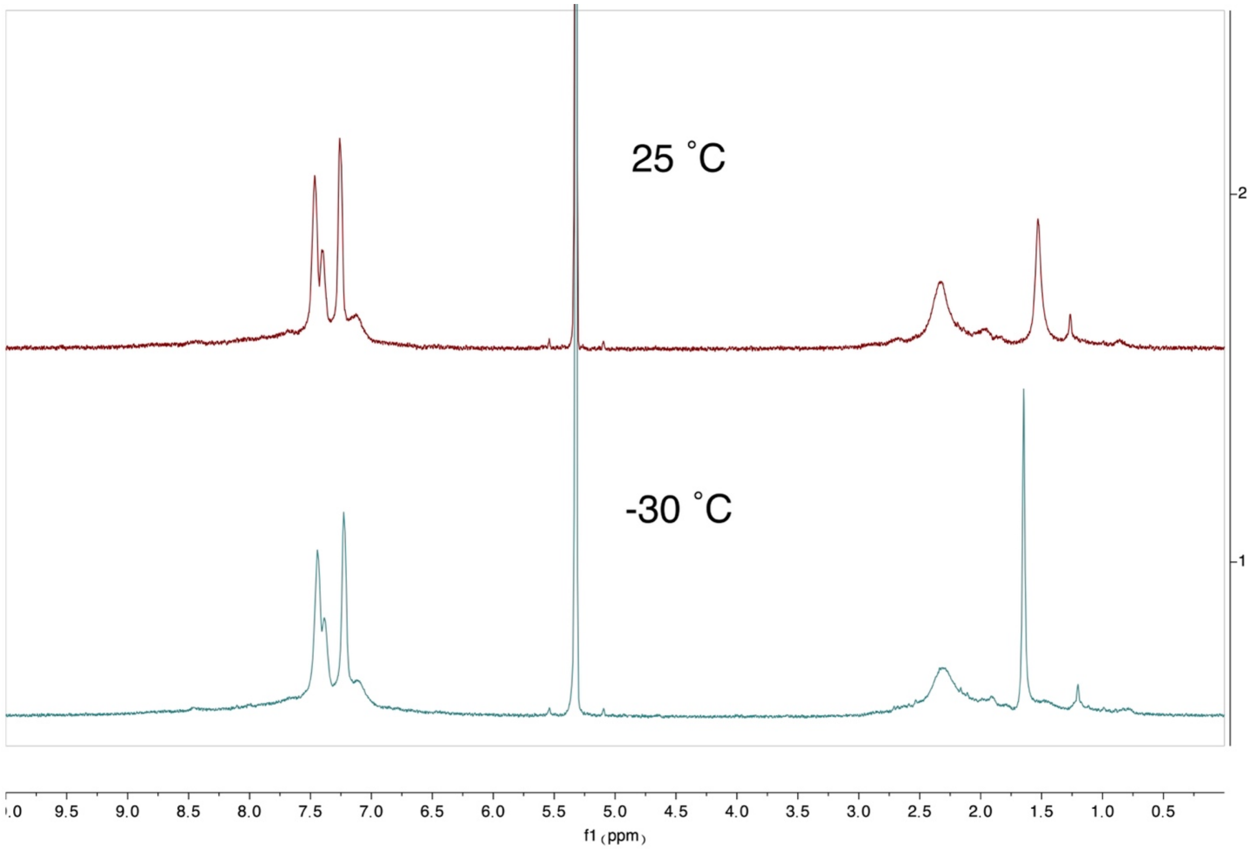

Figure S63. ^{1}H NMR (400 MHz) of **^{2Ph}FluIndz** in CD_2Cl_2 at room temperature (25 °C, top) and at -30 °C (bottom) showing no discernable improvement in resolution at the lower limit of our instrumentation in terms of temperature.

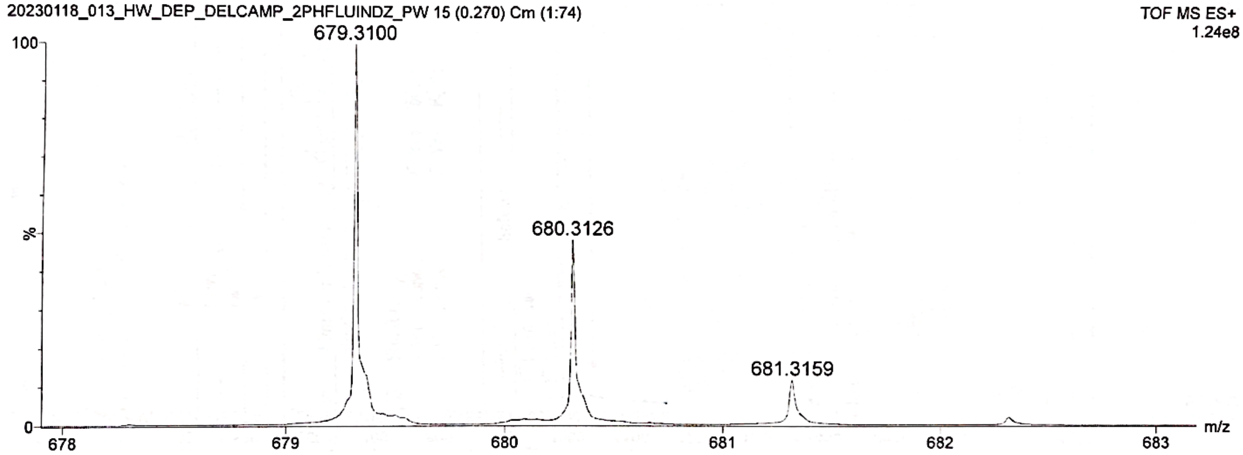

Figure S64. HRMS of **^{2Ph}FluIndz**.

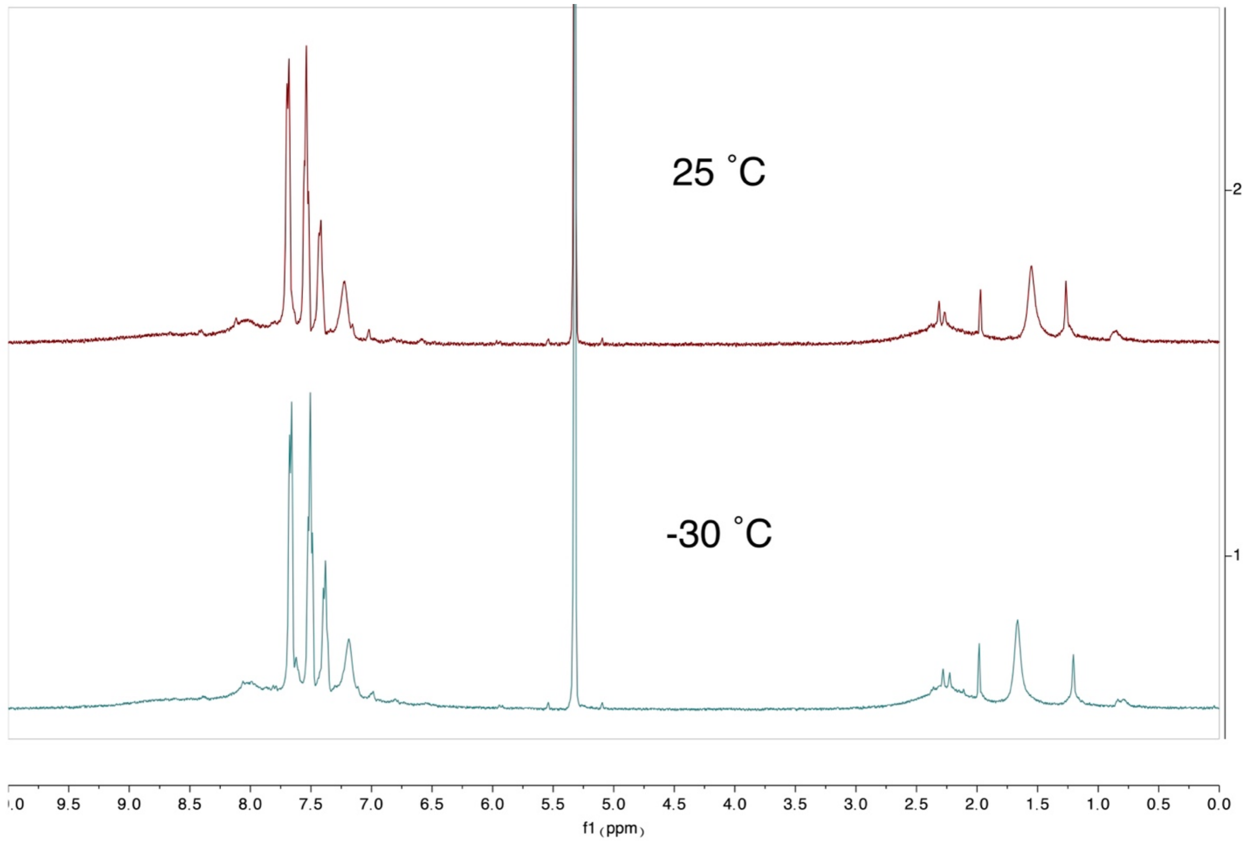

Figure S65. ^{1}H NMR (400 MHz) of **^{1Ph}FluIndz** in CD_2Cl_2 at room temperature (25 °C, top) and at -30 °C (bottom) showing no discernable improvement in resolution at the lower limit of our instrumentation in terms of temperature.

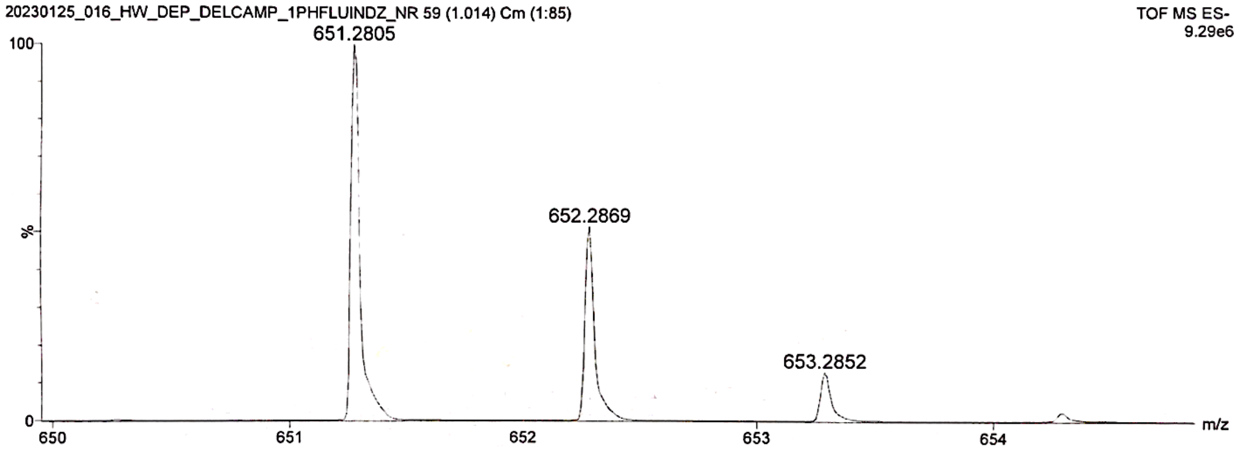

Figure S66. HRMS of **[1Ph]FluIndz**.

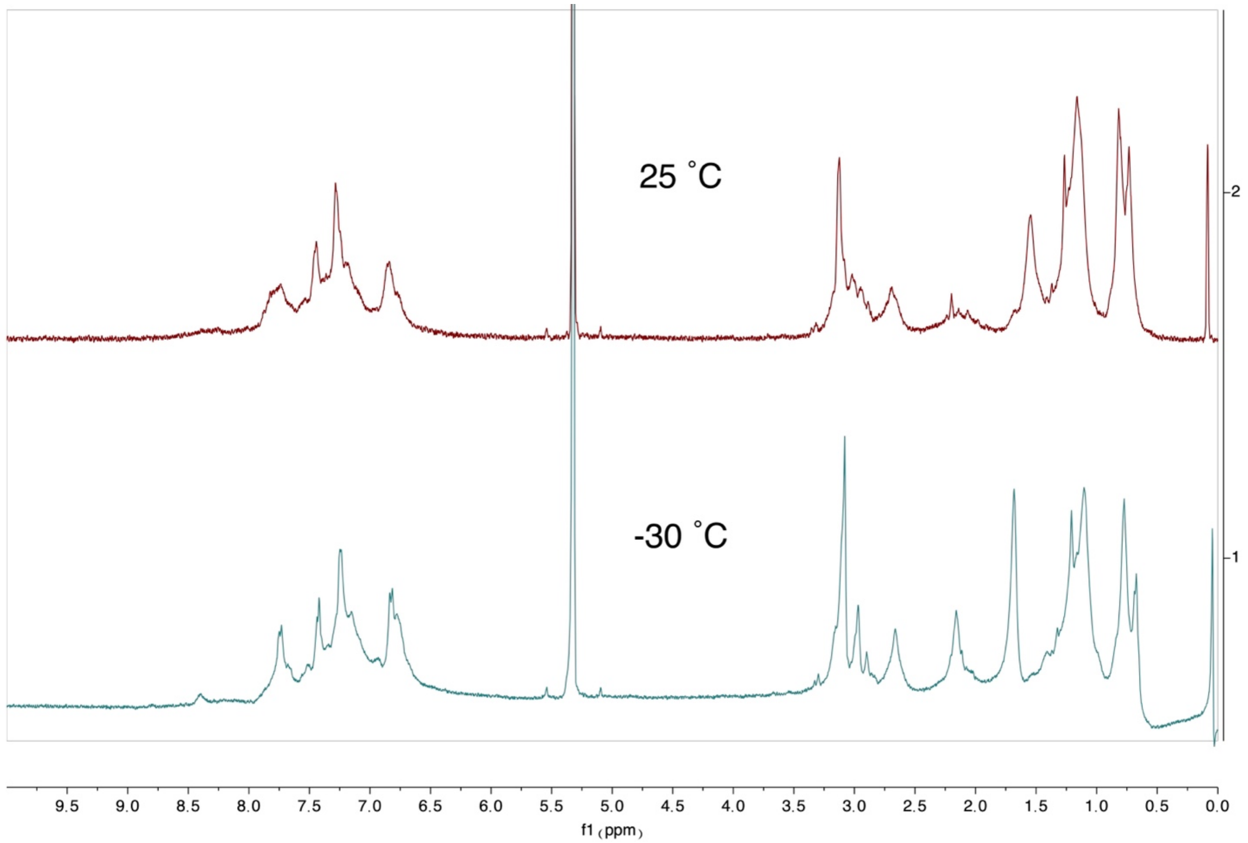

Figure S67. ^{1}H NMR (400 MHz) of $^{\mathbf{7DMA}}$**FluIndz** in CD_2Cl_2 at room temperature (25 °C, top) and at -30 °C (bottom) showing no discernable improvement in resolution at the lower limit of our instrumentation in terms of temperature.

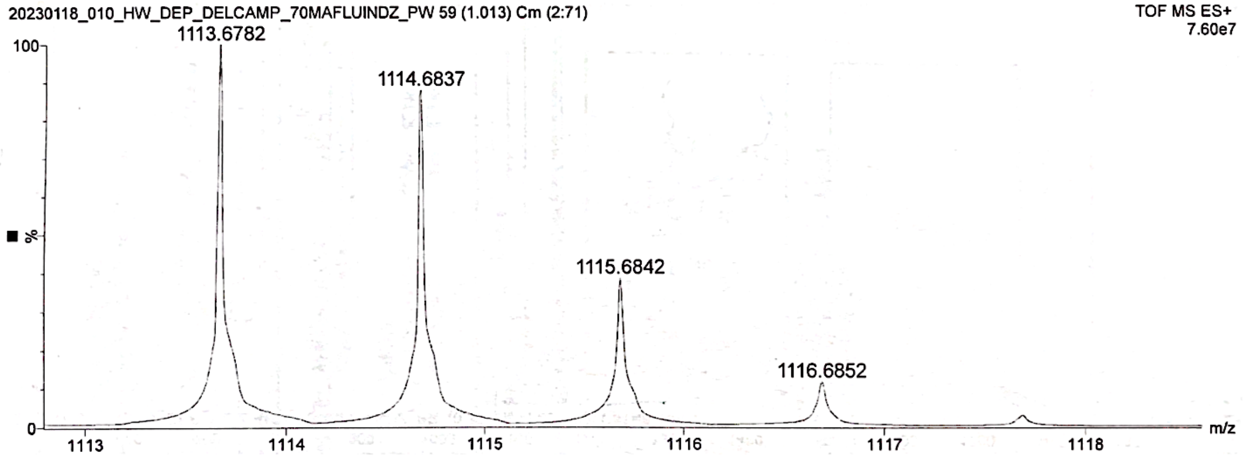

Figure S68. HRMS of **^{7DMA}FluIndz**.

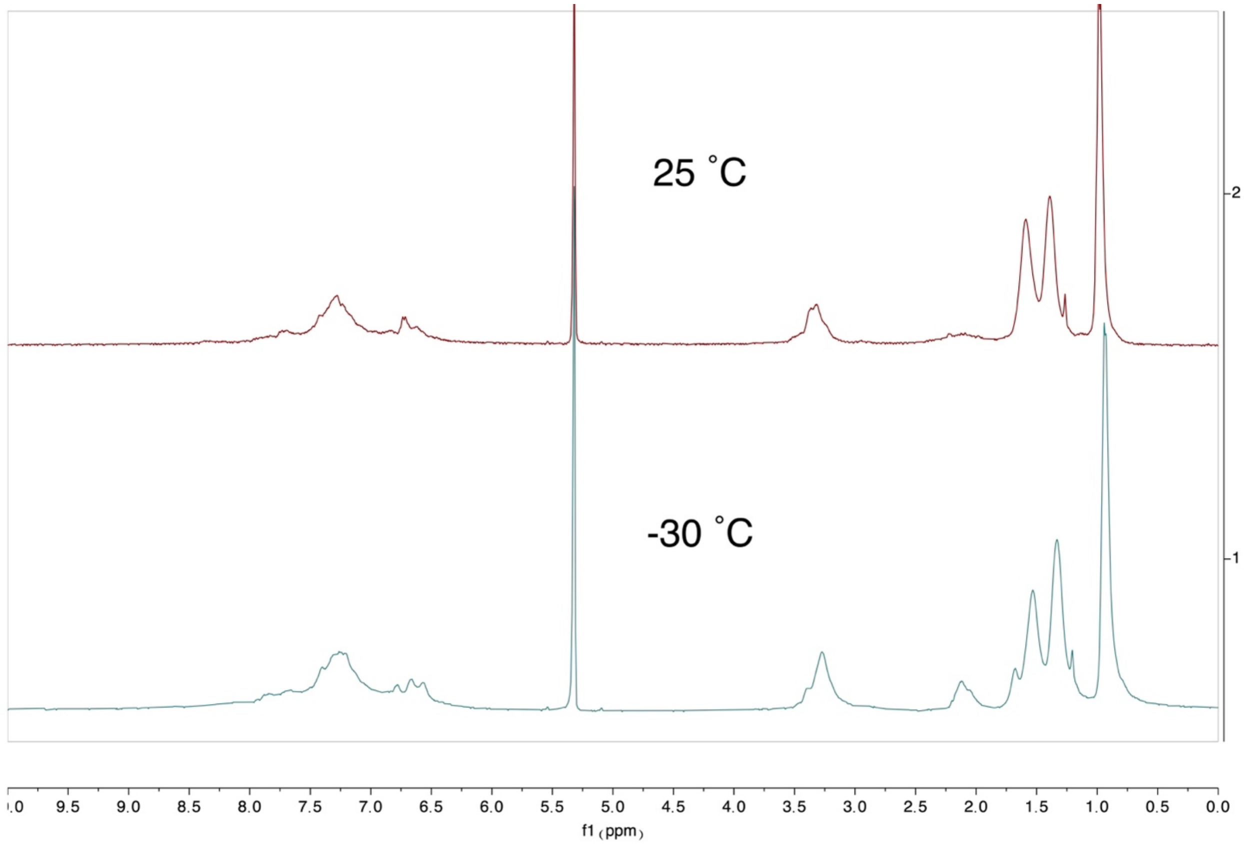

Figure S69. ^{1}H NMR (400 MHz) of **^{1,7DBA}FluIndz** in CD_2Cl_2 at room temperature (25 °C, top) and at -30 °C (bottom) showing no discernable improvement in resolution at the lower limit of our instrumentation in terms of temperature.

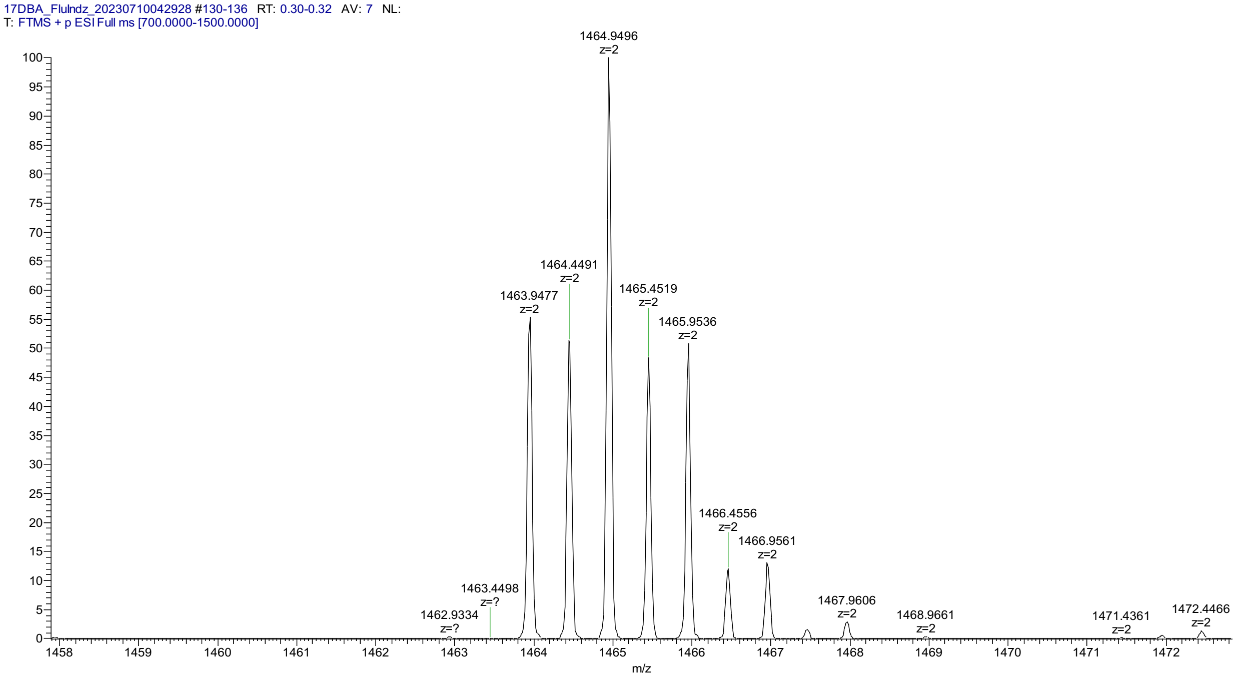

Figure S70. HRMS of **[1,7DBA]FluIndz**.

REFERENCES

[1.] N. G. Connelly and W. E. Geiger, *Chem. Rev.*, 1996, **96**, 877–910.
[2.] R. D. Foltz, *Handbook of Physics and Chemistry*, CRC Press, Boca Raton, FL, 63rd edn., 1982.
[3.] N. Ree, C. L. Andersen, M. D. Kilde, O. Hammerich, M. B. Nielsen and K. V. Mikkelsen, *Phys. Chem. Chem. Phys.*, 2018, **20**, 7438–7446.
[4.] H. Li, X. Wang, X. Li, S. Zeng and G. Chen, *Chem. Mater.*, 2020, **32**, 3365–3375.
[5.] J. B. Barton, J. C. Demro, R. Amber, G. Gasparian and M. Lange, *Defense Public Release: Technical Report*, 1998, ADA399438.
[6.] B. Chen, Y. Chen and Z. Deng, *Photonics*, 2021, **8**, 14.
[7.] H. Wen and E. Bellotti, *Journal of Applied Physics*, 2016, **119**, 205702.
[8.] Y. Yang, Y. H. Zhang, W. Z. Shen and H. C. Liu, *Progress in Quantum Electronics*, 2011, **35**, 77–108.
[9.] S. Chinnathambi and N. Shirahata, *Science and Technology of Advanced Materials*, 2019, **20**, 337–355.
[10.] V. Pejovic, E. Georgitzikis, J. Lee, I. Lieberman, D. Cheyns, P. Heremans and P. E. Malinowski, *IEEE Trans. Electron Devices*, 2022, **69**, 2840–2850.
[11.] K. Ba and J. Wang, *Materials Today*, 2022, **58**, 119–134.
[12.] M. Grzybowski, O. Morawski, K. Nowak and P. Garbacz, *Chem. Commun.*, 2022, **58**, 5455–5458.
[13.] W. E. Meador, E. Y. Lin, I. Lim, H. C. Friedman, D. Ndaleh, A. K. Shaik, N. I. Hammer, B. Yang, J. R. Caram, E. M. Sletten and J. H. Delcamp, *Nat. Chem.*, 2024, 1–9.
[14.] M.-H. Liu, Z. Zhang, Y.-C. Yang and Y.-H. Chan, *Angewandte Chemie International Edition*, 2021, **60**, 983–989.
[15.] Y. Yang, C. Sun, S. Wang, K. Yan, M. Zhao, B. Wu and F. Zhang, *Angew. Chem. Int. Ed.*, 2022, **61**, e202117436.
[16.] Y. Han, J. Zhu, S. Dong, Y. Eng, T. Tao, T. Y. Gopalakrishna and C. Chi, *Org. Lett.*, 2023, **25**, 3380–3385.
[17.] M. Imran, C. M. Wehrmann and M. S. Chen, *J. Am. Chem. Soc.*, 2020, **142**, 38–43.
[18.] C. M. Wehrmann, R. T. Charlton and M. S. Chen, *J. Am. Chem. Soc.*, 2019, **141**, 3240–3248.
[19.] Z. Wu, Y. Zhai, H. Kim, J. D. Azoulay and T. N. Ng, *Acc. Chem. Res.*, 2018, **51**, 3144–3153.
[20.] N. Li, Z. Lan, Y. S. Lau, J. Xie, D. Zhao and F. Zhu, *Adv. Sci.*, 2020, **7**, 2000444.
[21.] A. E. London, H. Chen, M. A. Sabuj, J. Tropp, M. Saghayezhian, N. Eedugurala, B. A. Zhang, Y. Liu, X. Gu, B. M. Wong, N. Rai, M. K. Bowman and J. D. Azoulay, *Science Advances*, 2019, **5**, eaav2336.
[22.] K. S. Mayer, D. J. Adams, N. Eedugurala, M. M. Lockart, P. Mahalingavelar, L. Huang, L. A. Galuska, E. R. King, X. Gu, M. K. Bowman and J. D. Azoulay, *Cell Reports Physical Science*, 2021, **2**, 100467.
